# Imputation of ancient canid genomes reveals inbreeding history over the past 10,000 years

**DOI:** 10.1073/pnas.2416980122

**Published:** 2025-11-24

**Authors:** Katia Bougiouri, Sabhrina Gita Aninta, Sophy Charlton, Alexander C. Harris, Martin Petr, Alberto Carmagnini, Giedrė Piličiauskienė, Tatiana R. Feuerborn, Lachie Scarsbrook, Kristina Tabbada, Povilas Blaževičius, Heidi G. Parker, Shyam Gopalakrishnan, Greger Larson, Elaine A. Ostrander, Evan K. Irving-Pease, Laurent A. F. Frantz, Fernando Racimo

**Affiliations:** ^a^Section for Molecular Ecology and Evolution, Globe Institute, University of Copenhagen, Copenhagen 1350, Denmark; ^b^School of Biological and Behavioural Sciences, Queen Mary University of London, London E1 4NS, United Kingdom; ^c^Department of Biology, University of Copenhagen, Copenhagen 2200, Denmark; ^d^BioArCh, Department of Archaeology, University of York, York YO10 5DD, United Kingdom; ^e^Cancer Genetics and Comparative Genomics Branch, National Human Genome Research Institute, NIH, Bethesda, MD 20892; ^f^Palaeogenomics Group, Department of Veterinary Sciences, Ludwig Maximilian University, Munich 80539, Germany; ^g^Department of Archeology, Faculty of History, Vilnius University, Vilnius 01513, Lithuania; ^h^The Palaeogenomics and Bio-Archaeology Research Network, School of Archaeology, University of Oxford, Oxford OX1 3TG, United Kingdom; ^i^National Museum of Lithuania, Vilnius 01143, Lithuania; ^j^Center for Evolutionary Hologenomics, Globe Institute, University of Copenhagen, Copenhagen 1350, Denmark

**Keywords:** ancient DNA, dog evolution, genotype imputation, runs of homozygosity, inbreeding

## Abstract

The multi-millennia-long history between dogs and humans has placed them at the forefront of archaeological and genomic research. Despite ongoing efforts including the analysis of ancient dog and wolf genomes, many questions remain regarding the evolutionary processes that led to the diversity of breeds today. Although ancient genome sequences provide valuable information about these processes, their utility is hindered by low depths of coverage and postmortem damage, which inhibits confident genotype calling. In the present study, we assess how genotype imputation of ancient dog and wolf genomes, using a large reference panel, can increase the amount of information provided by ancient datasets. We evaluated imputation accuracy by down-sampling high-coverage dog and wolf genomes to 0.05 to 2× coverage and compared concordance between imputed and high-coverage genotypes. We measured the impact of imputation on principal component analyses and runs of homozygosity (ROH). Our findings show high (R^2^ > 0.9) imputation accuracy for dogs with coverage as low as 0.5× and for wolves as low as 1.0×. We then imputed a dataset of 90 ancient dog and wolf genomes to assess changes in inbreeding during the last 10,000 y of dog evolution. Ancient dog and wolf populations generally exhibit lower inbreeding levels than present-day individuals. Regions with low ROH density maintained across ancient and present-day dogs were significantly associated with genes related to immunity and chemosensory receptors. Our study indicates that imputing ancient canine genomes is a viable strategy that allows for the use of analytical methods previously limited to high-quality genetic data.

## Significance

Ancient DNA is reshaping our understanding of the evolutionary history of dogs. Technical constraints, however, limit its applicability, such as inferring changes in inbreeding through time. Genotype imputation, a statistical methodology for inferring missing genotypes, presents a promising, yet unexplored, solution. We show that the imputation of ancient dogs and wolves is highly accurate. By imputing ancient dogs, we study inbreeding patterns over the past 10,000 y and find that a significant increase has occurred only in recent times and uncover that genomic regions resistant to homozygosity are significantly enriched for immunity and chemosensory receptor genes. We demonstrate the accuracy of imputation for ancient canids, paving the way for future studies to leverage the greater information content of phased haplotypes.

Among all domesticated species, dogs (*Canis familiaris*) are of unique public and scientific interest due to their extensive history with humans. Analyses of ancient dog and wolf genomes have advanced our understanding of their evolutionary history (1–13). However, these insights have been limited by the typically low coverage and degraded nature of ancient DNA (aDNA), which leads to elevated uncertainty in genotype calling and restricts the type of questions that can be confidently addressed with it (14–17). Common approaches to dealing with low-coverage aDNA data include “pseudohaploidization”—the random sampling of an allele at a given site, and genotype likelihoods, which incorporate genotype uncertainty due to read depth and base quality. However, both these approaches have substantial limitations, as many common methods used in population genomics were designed for high-confidence diploid genotypes with low error rates and low missing data.

A method that remains largely unused in canine aDNA studies is imputation—i.e., the statistical reconstruction of missing genetic variants based on haplotype similarity, using high-quality samples available from a large reference database (18). Unlike pseudohaploidization, which reduces the information content of modern genomes to match the low coverage of aDNA, imputation allows for improving the quality of ancient genomes by leveraging information from other genomes. Imputation is widely used in several types of genomic analyses, including genome-wide association studies using single nucleotide polymorphism (SNP) arrays (19–22) and population studies based on low-depth genome sequences (23–27).

Imputation of nonhuman animals has largely focused on model organisms and livestock, for which large reference panels are most abundant (28). Recent advances in computational algorithms have substantially improved imputation quality from low-coverage shotgun genomes (25, 27, 29). Such methods have produced highly accurate results in ancient samples from species for which large reference panels exist—e.g., humans (30) and cattle (31). However, species with reference panels lacking ancestral diversity show reduced accuracy (e.g., pigs) (32). Imputation of modern dogs has shown promising results as a method to increase SNP density (33–38), but the accuracy of imputation has not been previously investigated for ancient canids, nor have results from such been applied to questions of canine migration or domestication.

In this study, we developed an imputation pipeline for ancient dog and wolf genomes using a large reference panel consisting of 1,519 modern canids. We benchmarked its accuracy using 10 high-coverage (>10×) ancient and present-day dog and wolf samples representing different ancestries from Europe, Asia, Africa, and North America, which we downsampled to lower coverages. We further assessed the impact of imputation on principal component analysis (PCA) and runs of homozygosity (ROH). Our results demonstrate that high accuracy is achieved for coverages as low as 0.5× for ancient dogs and 1.0× for Pleistocene wolves. Based on these results, we imputed a worldwide dataset of 50 ancient dogs and 40 ancient wolves, spanning the last 100,000 y of canine evolutionary history. The imputed panel allowed us to estimate patterns of inbreeding over time in great detail. We observed generally stable levels of inbreeding in dogs over the course of the last 10,000 y, which were notably lower compared to the levels seen in present-day samples. We also assessed genomic regions with low ROH density (“ROH deserts”) across ancient and present-day samples and observed a significant enrichment for gene ontology terms related to immunity and chemosensory receptors.

## Results

### A Pipeline for Ancient Dog Genome Imputation.

We implemented a fully reproducible imputation pipeline using the GLIMPSE software and a reference panel of over 1,500 modern canids, available at https://github.com/katiabou/dog_imputation_pipeline. We applied this pipeline to the largest dataset of ancient dog and wolf genomes analyzed to date, including nine new dog genome sequences. We tested the accuracy of our imputation pipeline by downsampling seven high-coverage ancient and present-day dog samples and three Pleistocene wolf samples and assessed the concordance of the imputed genotypes against the original high-coverage genotypes. Based on the benchmarking results, we subsequently imputed 50 ancient dog and 40 ancient wolf genomes (total = 90).

### Imputation Accuracy Assessment.

**Fig. 1.**
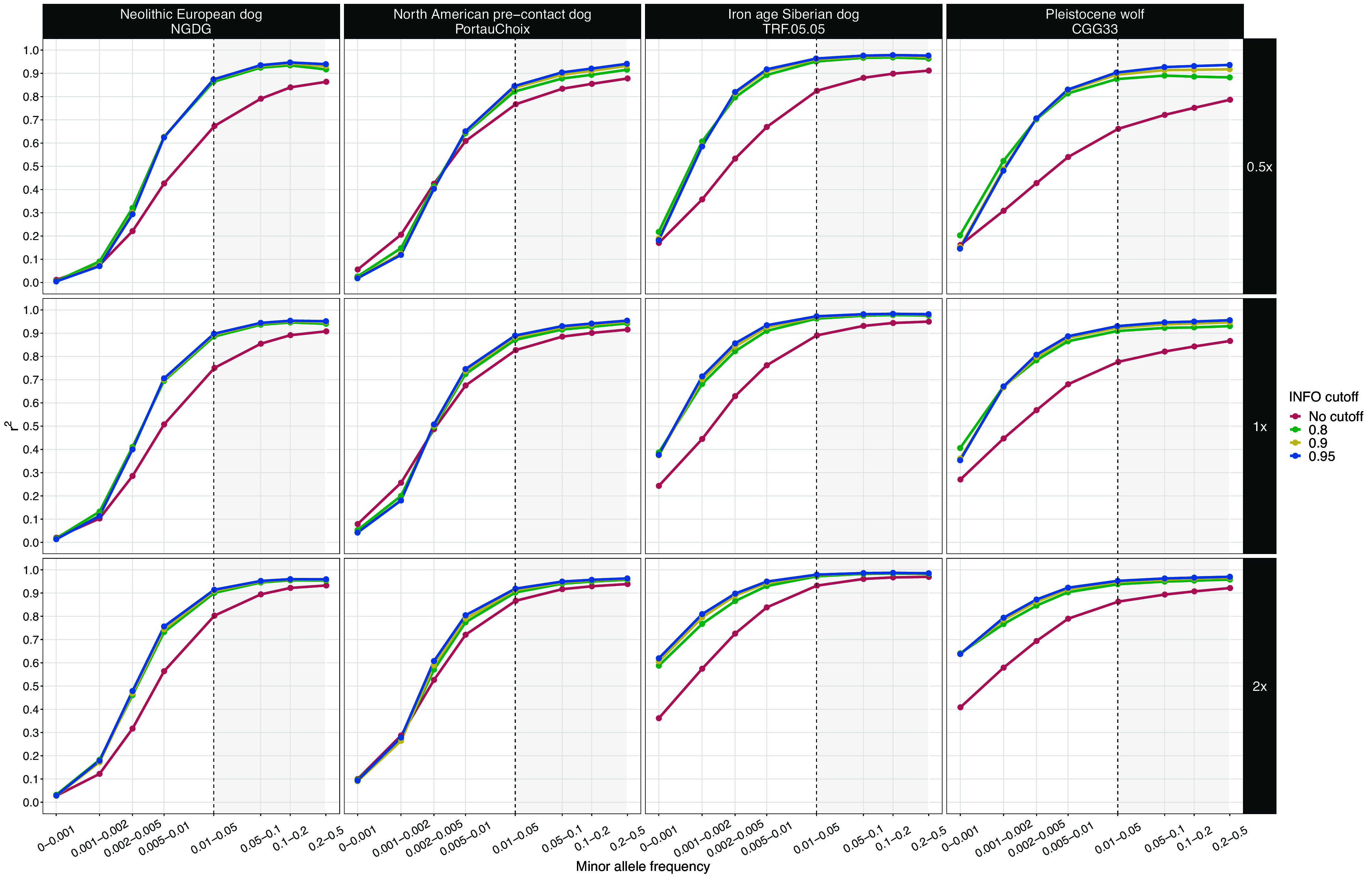
Squared correlation (r^2^) between imputed genotypes by GLIMPSE and highly confident called genotypes for four high-coverage samples (three ancient dogs and one Pleistocene wolf), at three downsampled coverage values (0.5×, 1×, and 2×) and across different MAF bins. Each color depicts the accuracy for a given INFO score cutoff. Red: no cutoff, Green: 0.8, Yellow: 0.9, and Blue: 0.95. Sites belonging to the MAF bins within the gray shaded area were retained after postimputation filtering.

Our analysis showed high concordance when imputing dogs as low as 0.5× (r^2^ > 0.9) and wolves as low as 1× coverage (r^2^ > 0.8) (Fig. 1 and *SI Appendix*, Figs. S4–S13). As expected, reduced accuracy (r^2^ < 0.8) was observed at lower levels of coverage and at sites with a lower minor allele frequency (MAF) (<0.01) in the reference panel. Applying an INFO score cutoff of 0.8, which removes low confidence imputed sites, increased concordance, although no further improvement was noticed under higher INFO score thresholds (0.9 and 0.95). All dog samples with ≥0.5× coverage and all wolf samples with ≥1× coverage reached an r^2^ plateau for sites with a MAF of ≥0.05, and in many cases as low as 0.01, demonstrating the accuracy with which GLIMPSE can impute common variants (*SI Appendix*, Figs. S4–S13). Among the various dog ancestries tested, ancient and historical Siberian and modern African and Asian village dogs showed high accuracy levels (r^2^ > 0.9), even at 0.2× coverage with a MAF cutoff of 0.01 (r^2^ > 0.9). One of the late Pleistocene wolves from Siberia (CGG33; ~17,000 y ago) showed similar accuracy levels (r^2^ > 0.9) for coverages from 1× and above. Results from imputation accuracy assessments in 1× ancient humans and 0.5× ancient cattle in previous studies yielded comparable accuracy levels to what we observed for the best performing ancestries, reaching a plateau of r^2^ > 0.9 at sites with MAF ≥0.02 (30, 31).

We observed low genotyping error rates (<10%) for most dog samples with coverage ≥0.5× when applying an INFO score cutoff of ≥0.8 (*SI Appendix*, Fig. S14). Overall, the error rate for homozygous reference and heterozygous genotypes was lower than 5% in both 0.5× dogs and 1× Pleistocene wolves. The 0.5× Port au Choix individual, a North American precontact dog, possessed the highest level of errors among heterozygous genotypes (12.1%). Genotyping errors for homozygous alternative genotypes were higher than for homozygous reference and heterozygous genotypes in all samples, with estimates ranging from 5.9% in a 0.5× dog downsampled genome from Iron Age Siberia to 12.4% in the 0.5× downsampled Port au Choix individual. Genotyping error rates for homozygous alternative genotypes were higher in Pleistocene wolves than in dogs (*SI Appendix*, Fig. S15).

We also looked at another measure of error, the nonreference discordance (NRD) rate, which gives weight to the incorrectly imputed alternative allele sites (homozygous or heterozygous) and not the homozygous reference sites, which represent the majority of sites. When applying an INFO score cutoff of 0.8, all 0.5× imputed dog samples showed NRD rates <10%, apart from the Port au Choix individual (NRD = 18.3%). The NRD rates for 1× Pleistocene wolves ranged from 7.9 to 15.9%. This is comparable to a recently imputed panel of ancient humans (30), where heterozygous and homozygous genotyping errors estimated from 1× ancient humans varied between <5 to 18% and <5 to 25% respectively, whereas NRD rates ranged between <5 to 29%.

For the majority of ancestries tested, dogs with ≥0.5× coverage and wolves with ≥1× coverage exhibited less than a 1% difference in genotyping errors associated with transversions only vs. all sites (*SI Appendix*, Figs. S16–S20), with the exception of the Port au Choix North American precontact dog (*SI Appendix*, Figs. S17 and S19). Specifically, at 0.5× coverage and 0.8 INFO score cutoff, genotyping rates decreased from 12.1 to 0.8% for heterozygous alternative sites, 12.4 to 11.6% for homozygous alternative sites and from 18.3 to 12.3% for the NRD rate. These results suggest that above our coverage cutoffs for all tested ancestries, apart from North America precontact dogs, there is no benefit to restricting imputed sites to transversions only.

In order to test whether the diversity of canid species within the reference panel influences imputation accuracy, we also ran our pipeline using a reference panel consisting only of dogs. Imputation accuracy of the 1× Pleistocene wolves decreased from r^2^ > 0.8 to r^2^ < 0.74 for sites within a MAF bin of 0.01 to 0.05, and minor changes were observed for 0.5× dogs (*SI Appendix*, Fig. S21). The overall number of sites retained after applying INFO score and MAF cutoffs was lower compared to using the all-canid reference panel (e.g., from 8,065,091 to 7,006,226 for a 0.5× Neolithic European dog, and from 6,215,645 to 5,184,364 for a 1× Pleistocene wolf (*SI Appendix*, Fig. S22). Error rates for homozygous reference and heterozygous sites were similar to the results obtained using the full reference panel (*SI Appendix*, Figs. S23 and S24). A large increase in genotyping error rates for homozygous alternative sites was observed for wolves, reaching as high as 56%, whereas NRD rates increased up to 30%.

Based on our results, we decided to include imputed samples with at least 0.5× coverage for ancient dogs and 1× for ancient wolves in subsequent analyses, using all canid genomes available in the reference panel, and filtering for sites with INFO scores of at least 0.8 and MAF above 0.01. Considering the potential loss of informative sites when filtering only for transversions (30.85% of all sites), we chose to keep all sites within the imputed dataset. Finally, we did not impute the North American precontact dog, due to the elevated genotyping errors that it showed (Dataset S1).

### PCA of Downsampled Imputed and Nonimputed Samples.

**Fig. 2.**
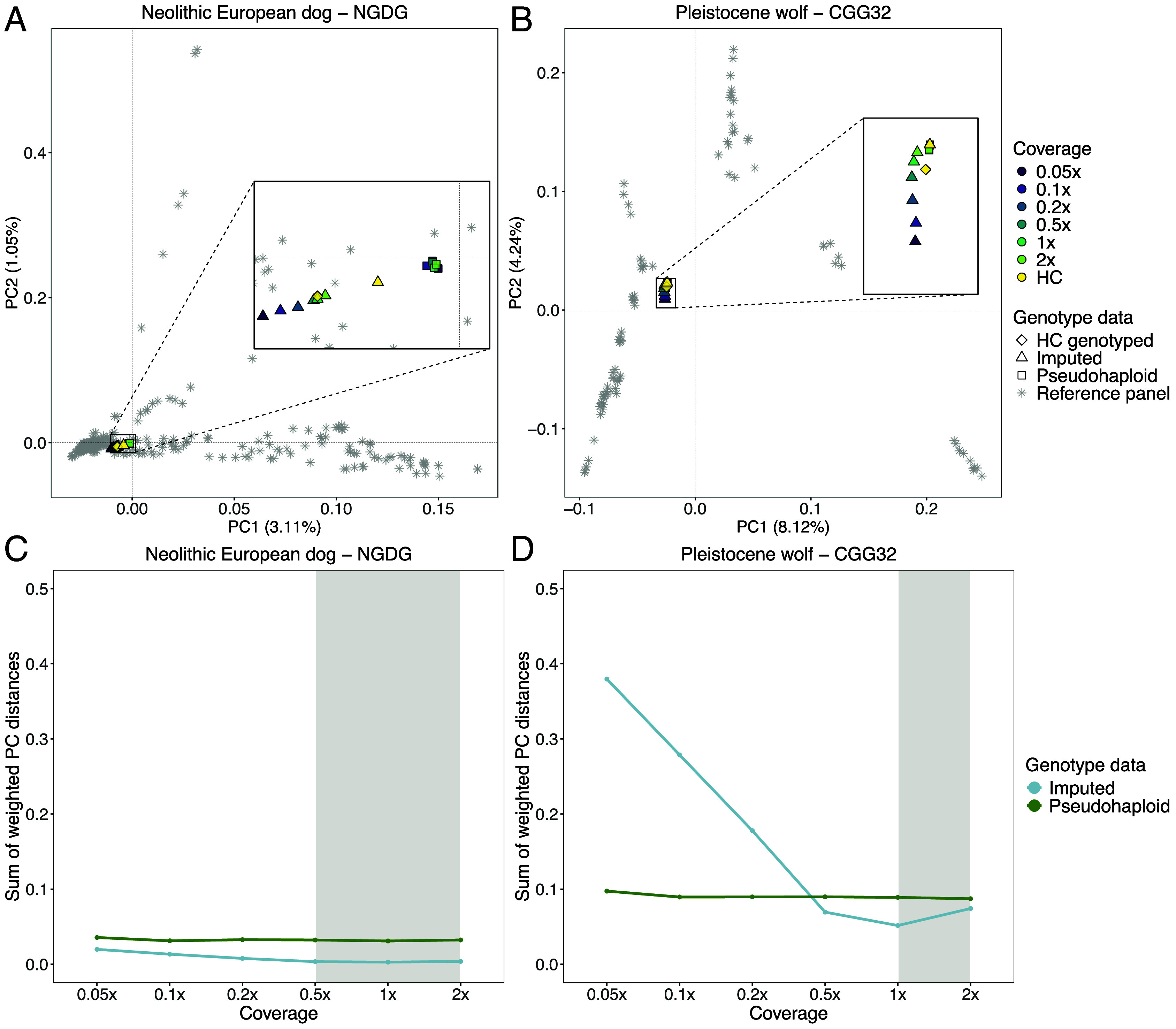
PCA demonstrating the placement of the nonfiltered imputed (*A*) Newgrange Neolithic European dog and (*B*) CGG32 Pleistocene wolf against their corresponding pseudohaploid counterpart in PCA space across all tested downsampled coverages. PCs were created using modern dog or wolf samples from the reference panel, and all versions of the ancient target sample were projected onto them. (*C* and *D*) Sum of weighted PC distances for each imputed (blue line) and pseudohaploid (green line) sample relative to the high-coverage ground truth sample across all tested coverages. The gray shaded area corresponds to the coverage cutoffs for dogs (0.5×) and wolves (1×) HC: high coverage.

To further assess the accuracy of the imputed genotypes, we carried out a PCA using each high-coverage sample as the ground truth. We then calculated the sum of weighted PC distances between each projected sample and their corresponding high-coverage samples across 10 PCs (Fig. 2 and *SI Appendix*, Figs. S25–S34). We tested this on the pseudohaploid samples, the imputed filtered (MAF ≥0.01 and INFO score ≥0.8), and imputed nonfiltered samples. For dogs, we noticed a better placement of the imputed samples (both filtered and unfiltered) in PCA space, compared to the pseudohaploid versions, for the majority of samples with coverages ≥0.5×. The PC distance between the 0.5× pseudohaploid dog samples and the ground truth ranged from being 1.2 times greater (for Iron Age Siberia, TRF.05.05) to 4.7 times greater (for Neolithic Europe, NGDG) compared to the distance between the 0.5× filtered imputed samples and the ground truth, and three imputed ancient samples showed better placement than the pseudohaploid genotypes for all tested coverages (Fig. 2 and *SI Appendix*, Figs. S25–S27). When applying postimputation filters, the placement of the imputed Pleistocene wolves performed worse across all coverages compared to their pseudohaploid counterparts. Imputed 1× and 2× Pleistocene wolf samples without any postimputation filtering, however, were on average 2 and 1.2 times closer to the ground truth compared to their corresponding pseudohaploid calls (Fig. 2 and *SI Appendix*, Figs. S32–S34).

### ROH in Downsampled Imputed and Nonimputed Samples.

**Fig. 3.**
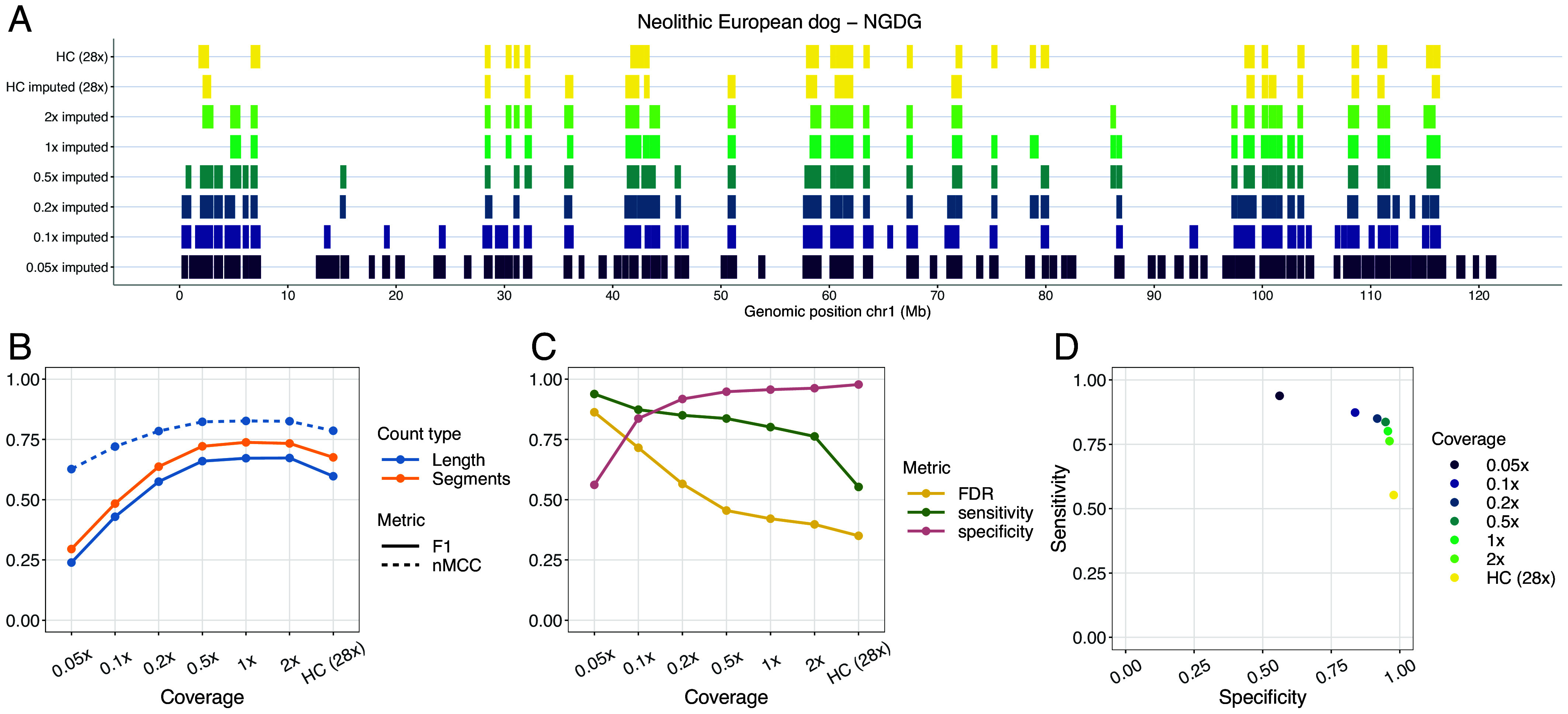
Overlap of ROH called from the Newgrange Neolithic European dog for each imputed downsampled replicate using ROH estimates from the ground truth. (*A*) ROH called across the six tested coverages and the high-coverage imputed and genotyped (ground truth) sample on chromosome one, including transversions and transitions. (*B*) Accuracy of recovering ROH across all tested coverages based on total length in bp (blue lines) and total number of segments (orange line) using the F1-score (solid line) and nMCC (dotted line). (*C*) FDR, sensitivity, and specificity measurements based on the total length of recovered ROH per coverage. (*D*) Sensitivity plotted against specificity estimated based on the total length of recovered ROH across all tested coverages. HC: High coverage.

We compared estimated ROH between imputed downsampled and high-coverage genotypes for all autosomes. Overall, overlapping ROH estimates varied among samples depending on the metric used [F1-score or normalized Matthew correlation coefficient (nMCC)], the reference used to estimate overlap (segment based or total length) and the sites included (transversions or transversions+transitions) (Fig. 3 and *SI Appendix*, Figs. S35–S44). Higher nMCC and F1-scores were observed when restricting the analysis to transversions only, with the highest difference observed for Port au Choix (*SI Appendix*, Figs. S35–S44).

Specificity scores (true negative rates) showed consistently high values (>0.8) across all tested individuals at coverages >0.2×. Sensitivity scores (true positive rates) typically showed a decreasing pattern with increasing coverage, starting from >0.8 at 0.05× coverage and decreasing to <0.1 at 2× coverage in the most severe scenario (Fig. 3 *C* and *D* and *SI Appendix*, Figs. S35–S44). Increased sensitivity at lower coverages appeared to be due to decreased false negatives at the cost of increased false positives (therefore lower specificity).

When compared to results from ROHan on nonimputed data, the ROH inferred from the imputed samples presented consistently higher nMCC scores, specificity estimates, and lower false discovery rates (*SI Appendix*, Figs. S45–S54). For cases below the recommended coverage (7×) for ROHan, ROHan would highly overestimate the total length of ROH, as reflected by the high sensitivity and false discovery rates and low specificity estimates observed when looking at samples between 0.5× and 2× (*SI Appendix*, Figs. S45–S49 and S52–S54). An increase in nMCC scores was observed in the high-coverage samples (11.2× to 28×). However, this was due to an underestimation of ROH as shown in the high specificity and low sensitivity rates. Even though the high-coverage samples were above the recommended coverage threshold for ROHan, ROH inferred from the high-coverage imputed samples consistently showed higher nMCC scores and sensitivity estimates.

### Imputed Ancient Dog and Wolf Dataset.

Based on our assessment of imputation accuracy, we imputed 50 ancient dog genomes with at least 0.5× coverage and 40 ancient wolves with at least 1× coverage (*SI Appendix*, Fig. S1). After merging all samples, we recalibrated the INFO scores and filtered for sites with an INFO score above 0.8 and for sites with a MAF above 0.01 in the reference panel, leading to a dataset of 10,518,332 SNPs. We subsequently merged the imputed dataset with a subset of samples from the reference panel (n = 502 dogs and n = 95 wolves) for downstream analyses. A PCA recapitulated known ancestry groups based on geography: European, African-Near East-India, Arctic, and East Asian (*SI Appendix*, Fig. S55).

### ROH in Ancient Dogs and Wolves.

**Fig. 4.**
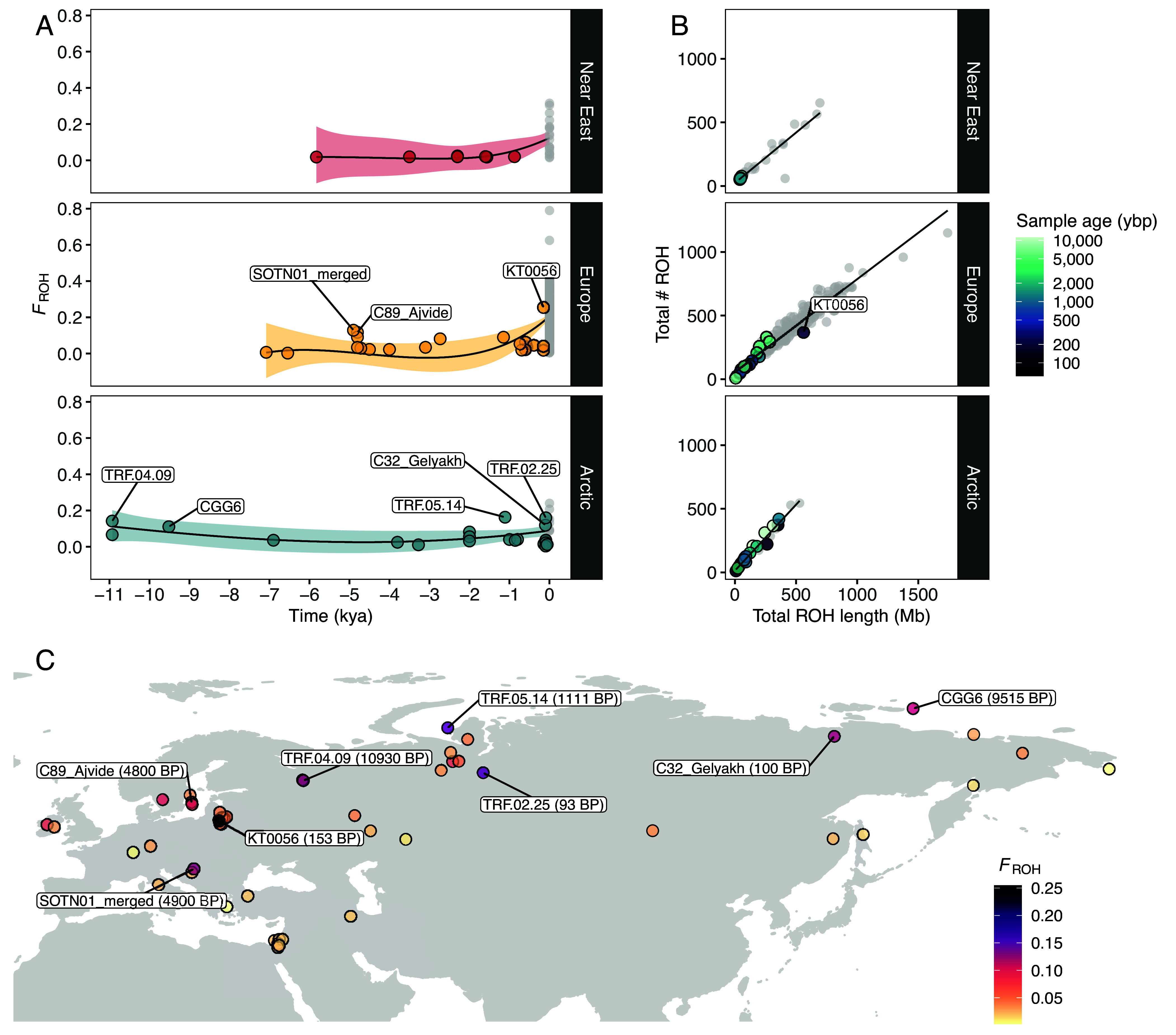
(*A*) Genomic inbreeding coefficient (F_ROH_) of imputed and modern dogs plotted as a function of time, calculated based on ROH. Imputed samples are colored based on their geographic grouping, while modern samples are colored in gray. A loess regression was applied with each colored shaded area depicting the SE. (*B*) Total number of ROH segments plotted against total ROH length for the imputed dogs. Colors correspond to age of imputed samples in years before present (ybp)., while modern samples belonging to each dog group are colored in gray. (*C*) Map of imputed dog samples colored by their inbreeding coefficient (F_ROH_). Samples with F_ROH_ values above 0.1 are indicated.

ROH were estimated for the ancient imputed and present-day samples using the same parameters in PLINK. Both transition and transversion sites were included. We estimated the total number and total length of ROH, as well as the ROH-based inbreeding coefficient for i) all ROH, ii) short ROH (<1.6 Mb), and iii) long ROH (≥1.6 Mb) (Fig. 4 and *SI Appendix*, Figs. S56–S62).

Overall, we observed remarkable stability in inbreeding for dogs during the past 10,000 y, until the beginnings of modern breed formation, which led to a substantial increase in the total number and length of ROH segments (Fig. 4, *SI Appendix*, Figs. S58, S59, and S63, and Dataset S5). Among ancient dogs, the highest inbreeding coefficients were calculated for Arctic and European individuals. Eight ancient dog samples from these regions had >10% of their genome located within an ROH (*F_ROH_* > 0.1) (Fig. 4): an early modern period Lithuanian dog (153 BP, *F_ROH_* = 0.26), an Iron Age and a historical dog from the Iamal-Nenets region (1,111 BP, *F_ROH_* = 0.17 & 93 BP, *F_ROH_* = 0.16), a Mesolithic dog from the Veretye site in Western Siberia (10,930 BP, *F_ROH_* = 0.14), a Neolithic dog from Croatia (4,900 BP, *F_ROH_* = 0.13), a historical dog from the Bulgunnyakhtakh site in Eastern Siberia (100 BP, *F_ROH_* = 0.12), a Swedish Pitted Ware sample from the island of Gotland (4,800 BP, *F_ROH_* = 0.12), and a Mesolithic dog from Zhokhov island in Eastern Siberia (9,515 BP, *F_ROH_* = 0.11).

In Europe, we observe a set of highly inbred individuals around 5,000 BP, with three dogs showing increased inbreeding coefficients, primarily due to a higher presence of short ROH segments (Fig. 4 and *SI Appendix*, Fig. S58): two individuals from the island of Gotland in Sweden dated to 4,800 BP and a Croatian dog dated to 4,900 BP. A Lithuanian dog from 153 BP showed the highest *F_ROH_* among ancient dogs (0.26), with an inbreeding coefficient substantially higher compared to eight other dogs from the same region and time period. The high *F_ROH_* of this sample was driven predominantly by the presence of long ROH (≥1.6 Mb), with four ROHs reaching more than 7 Mb in length (*SI Appendix*, Fig. S58). The ancient Arctic dogs showing highest *F_ROH_* coefficients did not appear to follow a specific temporal or geographic pattern. Ancient Near Eastern dogs showed the lowest *F_ROH_* coefficients with minimal fluctuations in inbreeding levels until the emergence of modern breeds.

Ancient Near Eastern *F_ROH_* estimates were significantly lower than ancient European (Mann–Whitney W = 29, *P* = 0.006) and Arctic (Mann–Whitney W = 33, *P* = 0.011), whereas ancient Arctic and ancient European did not differ statistically from each other (Mann–Whitney W = 217, *P* = 0.94) (Dataset S4). Present-day European dog *F_ROH_* levels were significantly higher than present-day Near Eastern (Mann–Whitney W = 2,750, *P* < 0.001) but not from present-day Arctic (Mann–Whitney W = 2,610, *P* = 0.06). Present-day Near Eastern and Arctic dogs did not show significant differences (Mann–Whitney W = 121, *P* = 0.48). All present-day breed dogs are significantly more inbred than the ancient individuals from their region of origin (Dataset S4). This is apparent when comparing maximum ROH lengths between present-day breed dogs and ancient European dogs, with the longest ROH recorded in our present-day dataset reaching ~37.5 Mb (Bouvier Des Flandres), while the longest ROH in our ancient dataset, identified in a 19th-century Lithuanian dog, was ~7.5 Mb.

The imputed wolves showed substantially low *F_ROH_* (<0.05) compared to present-day wolf populations (>0.5), with minimal fluctuations until modern times (*SI Appendix*, Figs. S60–S62 and S64 and Dataset S5). The *F_ROH_* levels in Pleistocene wolves remained low (<0.02), with only three samples showing *F_ROH_* > 0.01, which is driven by short *F_ROH_* (*SI Appendix*, Figs. S61 and S62). It is worth noting that some present-day wolves (from Sweden, Norway, and Mexico) showed higher *F_ROH_* levels than present-day breed dogs (*SI Appendix*, Figs. S59 and S62 and Dataset S5).

### Frequency of ROH across the Genome of Ancient and Present-Day Dogs.

**Fig. 5.**
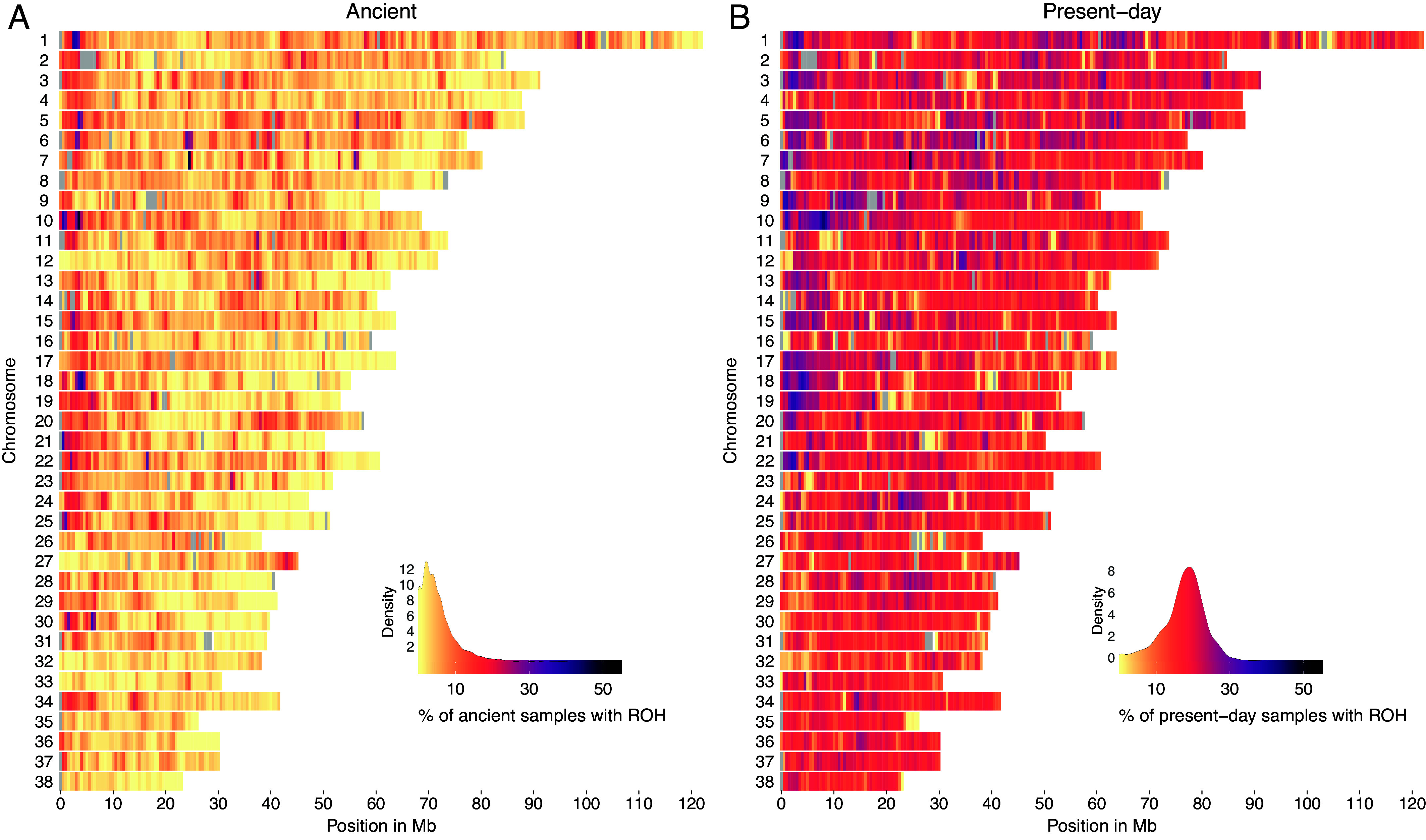
ROH across all chromosomes of (*A*) ancient dogs and (*B*) present-day dogs. The color legend represents the % of samples which have an ROH at each genomic position, with more yellow regions representing ROH deserts and more purple regions representing ROH islands. Gray colored regions indicate windows with an average depth of coverage estimated from all ancient dog samples above or below the mean ± 2* SD.

We estimated the prevalence of ROH across the genome of ancient and present-day dogs and wolves in 500 Kb windows. Fig. 5 and *SI Appendix*, Fig. S65 show the percentage of ancient and present-day dogs and wolves with an ROH in each window throughout all autosomes. Windows with average depth of coverage above or below the mean ± 2* SD were filtered out (*SI Appendix*, Figs. S66 and S67), and not included in the ROH frequency estimation. Ancient dogs showed a substantially lower prevalence of ROH across the genome than modern dog breeds. The majority of windows containing ROH were shared with less than 10% of all ancient dogs and approximately 20% of present-day dogs.

Given our ability to detect ROH at high resolution in a species that has undergone strong inbreeding in the very recent past, we aimed to investigate whether—even under strong inbreeding—certain regions would still be unlikely to harbor ROHs in dogs, due to evolutionary processes. We therefore examined ROH deserts, which we define as windows for which both less than 5% of the ancient and less than 5% of the present-day samples shared an ROH segment. To determine how surprising the number of desert windows we observe is—under our definition of a desert, and assuming ROH windows are randomly placed along the genome, and independently distributed among ancient and present-day samples—we used a randomization approach (see supplementary information and *SI Appendix*, Fig. S70). We find that the number of ROH desert windows we observe is highly significant under this model (*P* < 0.001, *SI Appendix*, Fig. S70), suggesting that the large number of ROH deserts we observed is likely driven by a biological (demographic and/or functional) process, including e.g., spatial correlation due to shared patterns of descent, or selection (dis)favoring ROH at particular regions of the genome. After removing windows with high frequency of copy number variants (CNVs) (*SI Appendix*, Figs. S68 and S69), we found 87 ROH deserts for dogs (2% of total windows included), for which the highest signals from the gene enrichment analysis showed an overrepresentation of genes related to immunity and chemosensory receptors, specifically for taste (FWER ≤ 0.2) (Dataset S6). In contrast, when examining ancient wolves using the same procedure, few samples shared an ROH in the same window (*SI Appendix*, Fig. S65), whereas 171 windows were identified as ROH deserts (3.9% of total windows included). Gene enrichment analysis also supported an overrepresentation of immunity genes (FWER ≤ 0.2) (Dataset S7). Even though the top gene ontology (GO) terms were related to the DLA region, we still retrieved significant hits related to immune functions in dogs and wolves after masking the DLA region from our GO enrichment analysis (Datasets S6 and S7).

## Discussion

### Imputed Ancient Dog and Wolf Genomes.

Our results show that it is highly beneficial to impute ancient dogs and ancient wolves based on present-day canid haplotypes. We can confidently impute data from ancient dogs with coverage as low as 0.5×, and ancient wolves as low as 1×, when applying the appropriate postimputation MAF (≥0.01) and INFO score (≥0.8) filters. These results were consistent across the major dog lineages tested: Arctic, European, African/Near Eastern, and Asian, for which the reference panel contained a sufficient number of present-day representatives such as modern breeds and village dogs.

An imputed North American precontact dog (Port au Choix) showed lower accuracy than other dogs. This lineage originated from Siberia and spread into the Americas 20,000 y ago (5, 10, 39). The isolation of this lineage and its near disappearance after the arrival of Europeans means that today this ancestry is not well represented in our panel. Therefore, we suggest that our reference panel does not contain sufficient haplotypes to impute low-coverage North American precontact dogs with high accuracy. This may also explain the relatively greater improvement in accuracy observed when restricting our analysis to transversion sites only for this sample, as transition sites, which are affected by aDNA damage, cannot be properly corrected by imputation if there is poor haplotypic representation of the relevant ancestries. Previous studies imputing ancient humans (30), horses (40), and pigs (32) have shown how imputation accuracy can change depending on the ancestral composition of the reference panel. We suggest that ancestry-specific coverage cutoffs should be applied prior to imputation.

The inclusion of non-dog canid haplotype donors in the reference panel substantially improved the imputation accuracy in ancient wolves and ancient dogs, albeit to a lesser extent in dogs. Non-dog canids such as wolves and coyotes may have maintained ancestral variation which was also present in ancient dog lineages, and which has now been depleted from modern dog breeds due to multiple bottlenecks and human artificial selection. Thus, including closely related canid species in the reference panel can assist in imputing sites in other ancient canids such as dogs and wolves. To our knowledge, including high-coverage ancient samples in reference panels has not yet been tested. Doing so may improve the accuracy of imputation in cases where that ancestry is poorly represented in the reference panel. However, care must be taken to avoid introducing bias from aDNA damage. Future work benchmarking this approach may provide further opportunities to impute ancestries which have scarce representation in present-day populations.

We note that additional post- or preimputation filtering approaches as tested in ref. 25 could potentially further improve the imputation of samples at lower coverages or with limited ancestral representation in the reference panel. This could be a focus of subsequent studies.

### Imputed vs. Pseudohaploid Genotypes in PCA Space.

In most cases, the projection of imputed genotypes outperformed the projection of pseudohaploid genotypes in PCA space, particularly for dog genomes above 0.5× and ancient wolf genomes above 1×. Three imputed target ancient dogs (Port au Choix, Newgrange, SOTN01) performed better across all coverages compared to their pseudohaploid versions. These results suggest that imputing diploid genotype data from each sample retains more information and can correct potential biases introduced when calling one allele per site, as done during pseudohaploidization.

For some samples with lower coverages (<0.2×), pseudohaploidization surpassed imputation performance (e.g., for Historical Siberia dog, Chinese Village dog, Nigerian Village dog). We attribute this to the larger proportion of sites filtered out in low-coverage samples after imputation. In those cases, higher uncertainty is expected at imputed sites with lower depth of coverage and/or among sites with less shared variation with the reference panel, which in turn means that more sites get filtered out when applying the INFO score cutoff. Effectively, this means that the pseudohaploid version of the sample ends up containing more sites than the imputed version, and so tends to be better placed in PCA space.

A notable difference was observed when comparing the placement of the imputed Pleistocene wolves. When applying postimputation filters, imputation performed worse than pseudohaploidization across all coverages, whereas applying no postimputation filters led to better performance for coverages above and equal to 1×. This suggests a trade-off between retaining fewer but more accurately imputed sites, vs. retaining more sites with higher uncertainty. Furthermore, the PCA made using present-day genetic variation may not provide the ideal space onto which to project ancestral genetic variation. In fact, previous studies have shown that most Pleistocene wolves belonged to now extinct lineages that are more genetically differentiated than present-day wolves and dogs (8, 13). Therefore, projecting these ancestries onto a PCA space determined by modern variation may produce misleading placements. All Pleistocene wolves formed a distinct cluster close to present-day East Eurasian Wolves. These Pleistocene wolves are distributed across Eurasia (Germany, Russia, and Belgium) and North America (United States and Canada), thus supporting the notion of a panmictic population without strong population structure throughout the Pleistocene (8). The Holocene Eastern Eurasian sample clustered with present-day Eastern Eurasian wolves, whereas the Holocene Western Eurasian wolf samples clustered with present-day Western Eurasian wolves (*SI Appendix*, Fig. S55).

Notably, we observed that the PCA placement of the HC imputed samples differed from that of the HC genotyped samples in all dog samples. This may be due to residual genotype errors in the HC genotyped samples which are corrected by imputation, or it may be due to imputation bias from the modern haplotypes in the reference panel.

Overall, the downsampled imputed samples were projected further from the HC genotyped in PCA space compared to the pseudohaploid ones, when the PCs were constructed using genetic variation that is distantly related to the target sample (e.g., Pleistocene vs. present-day wolves). For samples which belong to ancestries that are not well represented in the PCA space, we observed better performance when retaining more sites with higher genotype uncertainty (i.e., not applying any postimputation filters). Considering that imputation corrects for genotyping errors, we suggest that the imputed samples should be incorporated in the construction of the PCA space, rather than simply projected onto a preexisting PCA space, in order to capture the full patterns of genetic variation.

### Accuracy of ROH Estimation in Imputed Samples.

The number and length of ROH across the genome can reveal past demographic processes such as recent or past bottlenecks (41–44). However, estimating ROH in ancient samples presents challenges, due to low coverage and postmortem damage resulting in false heterozygous calls. The imputation of ancient samples can correct for genotyping errors and increase the density of diploid genotypes, thus facilitating more accurate ROH estimation.

We retrieved a high overall concordance of ROH segments that overlapped in the validation and imputed target samples. We found two specific scenarios where there were inconsistencies. First, we observed overestimation of ROH segments in the low-coverage (<0.2×) samples, likely due to lower imputation accuracy and to fewer SNPs retained following postimputation filtering, including many heterozygous sites. This leads to an overestimation of ROH throughout the genome, as shown in the high false discovery rates. Second, we observed underestimation of ROH segments in the higher coverage samples. We speculate there are two possible explanations for this. First, imputation may be correcting heterozygous sites, which were incorrectly called homozygous in the ground truth sample. Second, sites which were called heterozygous in the imputed samples may have been removed during the initial filtering of the ground truth samples (*SI Appendix, Methods, Validation Dataset Filtering*). We note that decreasing false discovery rates (FDR) were observed with increasing coverage across all target samples (Fig. 3*C* and *SI Appendix*, Figs. S35–S44).

Our ROH estimates from the imputed samples resulted in higher accuracy scores than those from ROHan based on the nonimputed version of the data. Given that ROHan is intended to detect ROH in ancient genomes with coverage no lower than 7× and with moderate DNA damage levels, such results are not surprising. This highlights the importance of our approach, which now permits the estimation of ROH in ancient dog samples at coverages as low as 0.5× and for wolves as low as 1×, as long as an appropriate imputation reference panel is available.

Echoing our benchmarking results, a lack of ancestral populations in the reference panel that are good representatives of the target samples led to reductions in imputation accuracy, which subsequently led to less accurate inference of ROH segments. This was the case for a North American precontact sample (Port au Choix). Even though accuracy for this sample improved somewhat when restricting to transversions, we note that while transversion-based ROH inference can be valuable for comparing relative inbreeding levels between individuals, the absence of transitions may hinder our ability to infer the total length and number of ROHs, potentially affecting downstream inferences about the time or extent of past population bottlenecks.

### Assessing Inbreeding Levels of Dogs and Wolves through Time.

The evolutionary history of dogs has been tightly linked with human movements, leading to founder events, bottlenecks, and admixture between populations (4–6, 11, 12, 45–47). This, in combination with intensive human-driven selective breeding to develop and maintain specific breed traits in more recent periods, has shaped dog genetic diversity through time. Previous studies on wolves have also found past and recent demographic events, including bottlenecks and within and between-species admixture (8, 9, 48–52). Even though wolves have not undergone the same human selective breeding as dogs, they have been subjected to a high degree of human-induced pressures via habitat loss and systematic persecution (48, 53–56). Subsequently, inbreeding levels in dog and wolf populations may have changed through time as the result of different factors.

We assessed inbreeding patterns in ancient dogs and ancient wolves using phased and imputed genomes. We found that inbreeding in dogs has predominantly occurred in recent times, with modern breeds containing significantly more ROH than ancient individuals across Eurasia. Despite the overall low levels of inbreeding in ancient samples, some individuals showed relatively high inbreeding coefficients with no clear temporal pattern. These include two Neolithic dogs from Croatia and the island of Gotland in Sweden (~4,800 BP), a Mesolithic dog from the Veretye site in Western Siberia (~11,000 BP), a Mesolithic dog from Zhokhov island in Eastern Siberia (9,515 BP), a 1,111 BP dog and a ~100 BP dog from the Iamal-Nenets region in Western Siberia, a 100 BP dog from the Bulgunnyakhtakh site in Eastern Siberia, and a ~150 BP dog from Lithuania. These sporadic increases in inbreeding were largely driven by samples from remote, and/or geographically isolated locations like Gotland and Northern Siberia. We hypothesize that small effective population sizes and limited gene flow were the primary causes of this pattern. The Lithuanian sample from the 19th century (KT0056), characterized by high levels of inbreeding, aligns with historical records that describe how noble owners of estates engaged in the selective breeding of unique hunting dog varieties. Frequently, these dogs were named after their noble breeders (e.g., Bialozar pointer, Kociol hound) (57).

Differences were also observed between present-day populations. Ancient European and Arctic dogs did not differ significantly in estimated ROH levels through most of their history. However, present-day European breeds display significantly higher inbreeding levels compared to Near Eastern breeds, likely due to specific and targeted breeding practices since the Victorian era, which led to the formation of European breeds.

High inbreeding levels were also observed for some present-day wolf populations, likely reflecting bottlenecks related to habitat fragmentation and recent population declines (58–60). The generally low levels of ROH observed in ancient wolves confirm previous findings supporting high connectivity and low differentiation of wolf populations throughout the Pleistocene (8). In their paper (8), found that despite the higher levels of differentiation in samples from the last 10,000 y, suggestive of population bottlenecks due to habitat fragmentation and human hunting, levels of individual heterozygosity remained the same. They attributed this to limited gene flow rather than a species-wide population decline. This would match our results, with low F*_ROH_* estimates maintained in the Holocene wolves.

Finally, our assessment of ROH frequency in present-day and ancient dog samples showed an enrichment for genes related to immunity and taste receptors among regions depleted of ROH. Intriguingly, both of these biological processes are deemed crucial for dogs, which strongly depend on chemosensory receptors for survival (61–63) and which have been subject to multiple pathogenic pressures throughout their history of cohabitation and migrations with humans (5, 64). This enrichment may be due to balancing selection for multiple variants associated with these functions, or to the presence of recessive deleterious variation within these regions, leading homozygous individuals to be at a disadvantage. We note, however, that a significant absence of ROH in a region essentially reflects selection occurring in the very last generation, relative to the sampled individuals. Though, given that our stringent criterion for what is considered an ROH desert applies to consistent absences of ROHs in both present and ancient individuals, we might also be capturing selection patterns that are due to more ancient events as well (e.g., repeated episodes of balancing selection). It is also possible that some of these signals may be driven by copy number variation, though we attempted to correct for this using strict coverage cutoffs, a strict filtering scheme for CNV regions, and a gene length correction in the GO enrichment test. We note that the resolution with which we can accurately resolve complex CNV regions in canids is currently limited by short-read sequencing data. Future studies incorporating long-read sequencing will be able to decipher in more detail the structural complexity and biological consequences of regions depleted of ROH. Finally, further investigation into these regions via formal tests of long-term selection, or via detailed functional characterization of the variants within them may shed more light on the causes for these patterns.

Imputed diploid genotypes of ancient samples can grant access to genomic tools mainly tailored for analyzing high-quality genomic data. This, in turn, can enable researchers to address problems that often require high-quality phased haplotypes, such as detecting natural selection, inferring and dating past admixture events, and estimating local ancestry tracts. The increase of sequenced ancient samples will inevitably fill spatiotemporal gaps in the evolutionary history of multiple species, including dogs and wolves. Testing and applying imputation methods on ancient genomes of sequenced species is a promising approach to maximize the genomic information retrieved from each sample, so as to better understand the evolutionary processes that shaped their past and present diversity.

## Materials and Methods

### Dataset.

82 publicly available ancient dog and wolf samples and nine newly sequenced historic dog samples were compiled for this study. Details of the archaeological context, laboratory protocols, and sequencing used for the newly generated data are described in *SI Appendix*.

### Imputation Pipeline.

We implemented an imputation pipeline on the ancient canid samples using GLIMPSE v1.1.1 (27) and a reference panel of 1,519 high-quality canids containing 29,813,378 biallelic sites (Dataset S2). In order to assess the imputation accuracy, we downsampled 10 high-coverage (>10×) dogs and wolves to six lower coverage levels (0.05×, 0.1×, 0.2×, 0.5×, 1×, 2×), imputed, and then compared the concordance between the downsampled imputed and high-coverage validation genotypes across MAF bins and INFO score cutoffs. We further evaluated genotype errors of homozygous reference, homozygous alternative, and heterozygous sites, as well as the NRD metric. Finally, we also tested how the imputation accuracy would change when i) restricting to transversions and ii) using a dog-only reference panel. Details of how the reference panel was compiled and filtered are available in *SI Appendix*.

### PCA.

PCA was carried out on the downsampled imputed and pseudohaploid samples to assess how imputation would affect placement in PC space. Pseudohaploid genotypes were called in angsd v0.94 (65). PCA was carried out with smartpca eigensoft v8.0 (66) using a subset of either dogs (n = 502) or wolves (n = 95) from the reference panel. The imputed and pseudohaploid replicates of each sample as well as its high-coverage genotyped version were projected onto the PC space using lsproject. For the PCA of the full imputed canid dataset, both imputed and present-day reference panel samples were used to create the PCs.

### ROH.

ROH were estimated on the downsampled imputed samples using PLINK v1.9 (67) and on the downsampled nonimputed samples using ROHan v1.0 (68). Using the GenomicRanges v1.50.2 R package (69) we estimated the ROH overlap between the downsampled and the high-coverage samples. Accuracy was assessed using the F1-score metric and nMCC. We inferred inbreeding levels on the full imputed canid dataset across space and time using PLINK v1.9, by estimating the total number and length of ROH along with the inbreeding coefficient *F_ROH_*. Using the windowscanr v0.1 R package (https://github.com/tavareshugo/WindowScanR), we estimated ROH prevalence across the genomes of ancient and modern dogs and wolves. We identified windows where <5% of ancient and <5% of present-day samples contained an ROH, and refer to them as ROH deserts. The statistical significance of the observed ROH desert regions was assessed using a custom-built randomization procedure (https://github.com/bodkan/dogs-randomization) (*SI Appendix, Randomization Procedure to Assess the Statistical Significance of ROH Desert Sharing*). Gene enrichment analysis was carried out on ROH deserts, after removing regions with high frequency of CNVs (*SI Appendix, Prevalence of ROH in Ancient and Modern Samples*), using the GOfuncR package (70). A full detailed description of the methods used in all analyses is found in the *SI Appendix*.

## Supplementary Material

Appendix 01 (PDF)

Dataset S01 (XLSX)

Dataset S02 (XLSX)

Dataset S03 (XLSX)

Dataset S04 (XLSX)

Dataset S05 (XLSX)

Dataset S06 (XLSX)

Dataset S07 (XLSX)

Dataset S08 (XLSX)

Dataset S09 (XLSX)

## Data Availability

Raw reads generated in this study have been deposited to the European Nucleotide Archive (ENA) under project number PRJEB73844 (71). The reference panel is available at https://sid.erda.dk/sharelink/d1p5Gd2PaB (72). Code used for all analyses shown is available at https://github.com/katiabou/ancient_dog_imputation_paper (73) and https://github.com/bodkan/dogs-randomization (74). The imputation pipeline can be found at https://github.com/katiabou/dog_imputation_pipeline (75).
