# Supplementary material for: Imputation of ancient canid genomes reveals inbreeding history over the past 10,000 years
Source: Proc Natl Acad Sci U S A. 2025 Nov 24;122(48):e2416980122. doi: 10.1073/pnas.2416980122 (PMC12684900; doi:10.1073/pnas.2416980122)
Supplement: Supplementary file 1 — Appendix 01 (PDF) [file pnas.2416980122.sapp.pdf]

Supporting Information for

Imputation of ancient canid genomes reveals inbreeding history over the past 10,000 years

Katia Bougiouri[a,1], Sabhrina Gita Aninta[b,c], Sophy Charlton[d], Alexander C. Harris[e], Martin Petr[a], Alberto Carmagnini[b,f], Giedrė Piličiauskienė[g], Tatiana R. Feuerborn[e], Lachie Scarsbrook[h], Kristina Tabbada[h], Povilas Blaževičius[g,i], Heidi G. Parker[e], Shyam Gopalakrishnan[j], Greger Larson[h], Elaine A. Ostrander[e], Evan K. Irving-Pease[a,1,2], Laurent A.F. Frantz1[b,f,1,2], Fernando Racimo[a,1,2]

Katia Bougiouri, Evan K. Irving-Pease, Laurent A.F. Frantz, Fernando Racimo

Email: katia.bougiouri@gmail.com, evan.irvingpease@gmail.com, laurent.frantz@lmu.de, fracimo@sund.ku.dk

This PDF file includes:

Supporting information text (Materials and Methods)
Figures S1 to S70
Legends for Datasets S1 to S9
SI References

Other supporting materials for this manuscript include the following:

Dataset S1 to S9

Supporting Information Text

Materials

Ancient data curation and assembly

We compiled a set of 82 publicly available ancient dog and wolf genomes from across Eurasia (1–9), along with nine newly sequenced medieval and early modern period dog genomes from Lithuania and Latvia (Dataset S1) (total ancient samples=91). The ancient dog samples (n=50) range in date from 100 years BP to more than 10,000 years BP, and the ancient wolf samples (n=40) date from 3,000 BP to more than 100,000 BP (Fig. S1). All genomes had a depth of coverage of at least 0.5x for ancient dogs and 1.0x for ancient wolves, following the results of imputation benchmarking (see section "Imputation Benchmarking"). The median depth of coverage for the ancient dogs was 3.7x (min 0.57x, max 33.3x) and for the ancient wolves it was 2.34x (min 1.0x, max 15.9x) (Fig. S1).

Archeological samples and context

Vilnius Lower castle, Lithuania (KT0033, KT0037, KT0039, KT0041, KT0043, KT0049, KT0052, KT0056)

Vilnius Lower castle was the central residence of the Grand Duke in the capital of the Grand Duchy of Lithuania from the early 14th to the middle of the 17th C AD. The zooarchaeological finds dating back from the 13th to the middle of the 14th C AD reflected the construction stages of the castle, and those of the late 14th to the 15th C AD represent the period of its prosperity. In the early 16th C AD, on the site of the castle, a new palace of the Grand Dukes of Lithuania was built, and this complex survived until the late 17th C AD. The castle was abandoned after a Muscovian attack in the middle of the 17th C AD, and completely demolished in the beginning of the 19th C AD. Canines analysed in this study were found during the archaeological excavations of 1988–2014, in the cultural layers dated to the 13th to 17th C AD. In Vilnius Lower Castle, an abundant zooarchaeological collection (NISP ca 80 000) with numerous dog remains (NISP 590, MNI 51) was collected and analysed. As historical records indicate, hunting was the main function of elite dogs in the Middle Ages and the early Modern Period. Therefore, dogs found in Vilnius Lower Castle and other elite residential environments were most likely used for hunting (10, 11).

Riga city, Latvia (KT0094)

An almost complete dog skeleton was found in 2006, during archeological excavations (12) at the site of a 14th-17th century AD cemetery near the St. Gertruda church at Brivibas Street 42/4 in Riga. Nonetheless, it appears

that the dog is not associated with the cemetery. It exhibits a notable pathology - knuckling, also known as carpal laxity syndrome.

Methods

Ancient DNA extraction, library preparation and sequencing

All aDNA laboratory work for the medieval and early modern period dog genomes from Lithuania and Latvia was undertaken in the dedicated ancient DNA laboratory within the PalePalaeogenomics & Bio-Archaeology Research Network (PalaeoBARN), School of Archaeology, University of Oxford. Between 47.7-68.5mg of bone powder was finely drilled from each specimen using a rotary dental drill at low speed or pulverised using a Retsch MM400 dismembrator at low speed. DNA was extracted using a modified version of the (13) protocol, designed specifically for short DNA fragments, but replaced the Zymo-Spin V column binding apparatus with a high pure extender assembly from the High Pure Viral Nucleic Acid Large Volume Kit (Roche 05114403001). Double-stranded Illumina libraries were prepared using the Blunt-End Single Tube (BEST) protocol outlined in (14), and quantitative PCR (qPCR) was used to assess the number of cycles necessary to amplify libraries to the concentration needed for sequencing by amplifying 1 uL of library with LabTAQ Green Hi Rox master mix (Labtech) and adapter-targeted primers on a StepOnePlus Real-Time PCR system (Thermofisher Applied Biosystems). Indexing PCR involved double indexing (15) and used AccuPrime I supermix (ThermoFisher) and the primers described by (14). PCR reactions were purified using AMPure XP beads (Beckman Coulter); fragment distribution was checked on a TapeStation 2200 (Agilent) with D1000 High Sensitivity screentapes and concentration was measured using a Qubit 3.0 (Thermofisher) fluorometer.

Initial screening was performed at the LMU Genzentrum, Munich, Germany on a NextSeq 1000 P2 flowcell (100 bp Single End run). Deeper sequencing was then undertaken at the National Institutes of Health USA on the NovaSeq 6000 Sequencing System with paired end sequencing and 150 bp reads. The data generated for this study have been deposited to the European Nucleotide Archive (ENA) under project number PRJEB73844.

Ancient genome data preparation

Paired-end data reads were trimmed of adaptors and collapsed using adapterRemoval v2 (16) and mapped with BWA aln v0.7.17 (17, 18) to the CanFam3.1 dog reference genome (19) using the following parameters: -l 16500

-n 0.01 -o 2. We used FilterUniqueSAMCons (20) to remove duplicate reads with the same orientation and same start and end coordinates.

Imputation pipeline

To account for the genotype uncertainty in low-coverage ancient sequences, we phased and imputed the ancient dog and wolf dataset using GLIMPSE v1.1.1 (21), which has been shown to produce highly accurate phased haplotypes from ancient DNA, when used with a large and representative reference panel (22). The imputation pipeline can be found at https://github.com/katiabou/dog_imputation_pipeline.

Reference panel

We compiled a large and globally diverse canine reference panel, consisting of 139,268,526 variants and 1,697 whole-genome samples, including modern breed dogs (n=1,393) representing 237 dog breeds, village and indigenous dogs (n=111), New Guinea singing dogs (n=15), dingoes (n=32) and wild canids (n=146). These included grey wolves (n=116), African golden wolves (n=6), African wild dogs (n=3), jackals (n=5), coyotes (n=9), a dhole (n=1), an Ethiopian wolf (n=1), a grey fox (n=1) and red wolves (n=4) (Dataset S2).

We used BWA mem v0.7.17 (18) to perform the FASTQ alignment, which was then sorted using samtools v1.12 (23). The GATK v4.1.8.0 MarkDuplicates tool (24) was then used to tag duplicate reads. GATK BaseRecalibrator was used to generate BQSR recalibration tables using CF31_dbSNP_v151.vcf as the known sites, followed by the GATK ApplyBQSR tool to apply the recalibrations to the samples. The GATK Haplotypecaller (25) was used to emit all active sites in GVCF mode and generated GVCF files from the BQSR bam file in preparation for cohort calling. The GATK GenomicsDBImport tool was used to collate the GVCFs together. For parallelising purposes, the importation was done in approximately 5 MB intervals using natural gaps in the CanFam3.1 genome. GATK GenotyopeGVCFs was then used on these shards to generate region based VCFs which were then merged using the GATK GatherVcfsCloud tool. The resulting VCF had VQSR recalibration as described in (26).

We filtered for samples with a minimum depth of coverage (DoC) of 8x (Fig. S2), and excluded all boxer breed samples (to avoid reference bias from alignment to the CanFam3.1 assembly). If duplicates of the same sample were present, the lowest coverage member of the pair was removed. This resulted in a final dataset of 1,519 high-quality samples which was used as the imputation reference panel. This included 1,277 breed dogs represented by 228 breeds, 80 village dogs and indigenous dogs, 29 dingoes, 14 New Guinea singing dogs, and 119 wild canids which

included 101 grey wolves, 1 dhole, 3 jackals, 1 grey fox, 6 coyotes, 4 African golden wolves, 2 African wild dogs, 1 red wolf and 1 Ethiopian wolf.

We filtered sites using bcftools v1.15.1 (23) to retain only biallelic SNPs which passed variant quality score recalibration with GATK, and removed sites with a fraction of missing genotypes greater than 5%; resulting in 29,480,023 sites in the autosomes. We subsequently phased the reference panel using shapeit v5.0.1(27).

Imputation of ancient dog and wolf dataset

We imputed the ancient dog and wolf dataset per chromosome following the recommended GLIMPSE workflow (Fig. S3) by: i) computing genotype likelihoods for each sample, restricting to the sites and alleles ascertained in the filtered reference panel, using bcftools v1.15.1 (23) 'mpileup' function with the flags '-I -E -a "FORMAT/DP"' and the 'call' function with the flags '-Aim -C alleles'; ii) splitting each chromosome into chunks using a window size of 2 Mb and a buffer size of 200 Kb using GLIMPSE_chunk; iii) imputing each chunk using the genotype likelihoods of each sample, the reference panel haplotypes and the CanFam3.1 genetic map (28) using GLIMPSE_phase; and iv) ligating the chunks of each chromosome using GLIMPSE_ligate. We also carried out phasing of haplotypes with the '–solve' flag using GLIMPSE_sample. We subsequently applied post-imputation filtering based on the imputation accuracy assessment results (see below), removing sites below an INFO score of 0.8 and a minor allele frequency (MAF) cutoff of 0.01 in the reference panel.

Imputation benchmarking

We benchmarked GLIMPSE to test how accurately it can impute low coverage ancient dog and wolf samples using our reference panel, and to determine the best empirical cutoffs for post-imputation filtering. We chose 10 high coverage (>10x) targets representing different ancestries and time periods; including two late Neolithic European dogs (4,800 BP and 4,900 BP), one North American pre-contact dog (4,157 BP), one historical (60 BP) and one Iron Age (2,000 BP) Siberian dog as well as two present-day village dogs from Nigeria and China (since no ancient representatives of African and Asian ancestry are currently available), and three Pleistocene wolves (16,800 BP, 32,000 BP and 50,000 BP) (Dataset S3).

We downsampled each high-coverage genome to six lower coverage levels (0.05x, 0.1x, 0.2x, 0.5x, 1x and 2x) using samtools v1.15.1 (23). We then followed the same GLIMPSE workflow as above, imputing each

downsampled target individual separately. Modern samples (i.e. those included in the original reference panel) were removed from the reference panel for the benchmarking.

We subsequently used the GLIMPSE concordance tool (GLIMPSE_concordance) to test for concordance between the downsampled imputed genotypes and the high coverage validation genotypes (see validation dataset section below). We assessed how MAF and INFO cutoff scores (0.8, 0.9, 0.95) affected concordance values. INFO scores indicate the level of uncertainty in the posterior genotypes probabilities of each imputed site. We computed concordance across MAF and INFO scores using both all sites and transversions only. We ran the GLIMPSE_concordance tool using the following flags '-minDP 8 -minPROB 0.9 –af-tag AF –bins 0.00000 0.00100 0.00200 0.00500 0.01000 0.05000 0.10000 0.20000 0.50000', as suggested in the GLIMPSE manual (https://odelaneau.github.io/GLIMPSE/glimpse1/).

The GLIMPSE concordance tool also provides metrics of genotyping errors for homozygous alternative, heterozygous and homozygous reference alleles. It also outputs the non-reference discordance (NRD) metric, which only takes into consideration imputation errors at alternative alleles by excluding confidently imputed homozygous reference alleles. This is equal to:

$$NRD = (e_{RR}+e_{RA}+e_{AA}) / (e_{RR}+e_{RA}+e_{AA}+m_{RA}+m_{AA})$$

where e_{RR}, e_{RA}, e_{RR} are the mismatches at homozygous reference, heterozygous and homozygous alternative alleles respectively, whereas m_{RA} and m_{AA} are the matches at heterozygous and homozygous alternative alleles. We also tested how the amount of canid haplotype diversity present in the reference panel influenced imputation accuracy by using a dog-only reference panel in a separate imputation analysis (n=1,399 and 18,497,052 sites).

Validation dataset filtering

To limit the impact of genotyping errors in our benchmarking pipeline, we applied the following filters on the 10 high coverage samples used for benchmarking while using the bcftools 'mpileup' and 'call' functions, following (22): i) reads with mapping and base quality below 30 (-q 30, -Q 30) were removed and the '-C 50' option was used to downgrade mapping quality for reads containing excessive mismatches; ii) sites with QUAL lower than 30 were excluded; iii) sites with extreme values of depth of coverage (i.e., sites with a depth of coverage greater than twice the mean genome-wide depth, and sites with a depth below either 8x or one third of the mean depth of coverage (i.e., max(DoC/3, 8)), whichever is greater were also excluded; and iv) heterozygous sites at which the one of the two

alleles was found in less than 15% or more than 85% of the reads using bcftools v1.15.1 'view' and the flags '–exclude 'GT="het" && ((INFO/AD[1] / INFO/DP < 0.15) || (INFO/AD[1] / INFO/DP > 0.85))'.

PCA of imputed samples

Downsampled target samples

We next assessed how imputation of low coverage samples would affect their placement in PCA space in comparison to pseudohaploid data. To do this, we called pseudohaploid genotypes in the downsampled target samples using the -doHaploCall function in angsd v0.94 (29), the -doCount 1 option, filtering for a minimum base and map quality of 30 (-minMapQ 30, -minQ 30), trimming five base pairs at the beginning and end of each read (-trim 5) and restricting to transversion sites (-noTrans 1). We applied a minimum MAF (0.01) and INFO score (0.8) cutoffs in the imputed samples (based on the benchmarking, see results) to assess how this compares to unfiltered imputed genotypes. After filtering the pseudo-haploid dataset for sites present in the filtered reference panel, we merged it with the high-coverage genotyped validation samples, the imputed samples (high-coverage and downsampled) and the filtered reference panel.

We subsequently carried out PCA using smartpca eigensoft v8.0 (30). For our ancient dog PCA we used a reference panel of 502 present-day dogs, and for our ancient wolf PCA we used a reference panel of 95 present-day wolves (Dataset S2). We projected the imputed and pseudohaploid replicate of each target sample along with its genotyped high coverage version onto the PCA using the lsproject option. We subsequently estimated the sum of weighted PC distances between each downsampled target (imputed and pseudohaploid) and the genotyped high-coverage counterpart (used as the ground truth) across the first 10 principal components.

Imputed ancient dog and wolf dataset

Prior to the PCA of the full imputed ancient dataset, we merged all imputed ancient samples into the same VCF and re-calibrated the INFO scores in order to maintain a consistent filtering of sites across individuals. We applied MAF≥0.01 and INFO≥0.8 cutoffs based on the benchmarking results. We again applied the smartpca tool of eigensoft, this time using the imputed and present-day reference panel samples to create the first 10 principal components. The present-day reference panel was filtered only for sites present in the merged imputed dataset. We ran a PCA for dog and wolf samples separately, using either present-day dogs or present-day wolves respectively.

Runs of homozygosity of imputed samples

Prior to estimating ROH, we applied MAF (0.01) and INFO score (0.8) cutoffs on each of the imputed samples (i.e., prior to INFO score recalibration in the merged callset). We used the PLINK v1.9 (31) (www.cog-genomics.org/plink/1.9/) --homozyg tool to estimate ROH, carrying out two runs: i) only including transversions and ii) including both transversions and transitions. In both runs, the following parameters were set: --homozyg-density 50, --homozyg_gap 500, --homozyg_kb 500, --homozyg_snp 50, --homozyg_window_het 1, --homozyg_window_missing 5, --homozyg_window_snp 50, --homozyg_window_threshold 0.05. We chose these parameters following published recommendations for ancient samples (22, 32). We chose the PLINK parameter --homozyg-window-het 1, consistent with the ancient DNA literature (22, 32, 33) and with some present-day studies (34–36). However, we note that this configuration allows an unlimited number of heterozygous SNPs across a putative ROH block, as long as no more than one heterozygous SNP appears in a sliding window of size --homozyg-window-snp 50. As such, the biological interpretation of these loci should be that they are regions of low diversity, rather than strictly uninterrupted runs of homozygosity. This applies to all published literature where no upper bound is specifically set with the flag --homozyg-het. The same set of parameters was used for the downsampled imputed target samples, the high coverage genotyped target samples, the full imputed ancient dataset and the reference panel. For the ROH analysis, a MAF 0.01 filter was applied to the reference panel.

ROH estimates using ROHan

As part of our benchmarking approach, we compared our results to ROHan v1.0 (37) - a method designed to infer ROHs on ancient medium-coverage data (at least 7X) that has not been imputed. We used ROHan to infer ROHs on the non-imputed downsampled and HC targets to compare against the inferred ROH on the imputed ones from our pipeline. For the ancient genomes, we first ran the `bam2prof` utility of ROHan to obtain the deamination pattern from the first 5 base pairs of the 5' and 3' prime end at each downsampled coverage and consider a minimum base quality of 20 (-minq 20 -minl 5). The resulting deamination profile of each sample at each coverage was then run along with the BAM file in ROHan (via option —deam5p and --deam3p) using the default parameters, except for the number of heterozygous sites (--rohmu) which was set to 4 x 10^{-5} and the sliding window (--size) which we ran on the default 1Mbp (we also tried a smaller window size to match the window size of 500Kbp of our imputation pipeline, but this resulted in lower accuracy estimates). The modern genomes were run similarly without the deamination profile option.

ROH accuracy assessment

To assess the accuracy of inferred ROH blocks in the downsampled imputed samples, we estimated the ROH overlap with the high-coverage samples with respect to the total number of segments and total length of overlapping bases, using the GenomicRanges v1.50.2 R package (38). For both approaches, we calculated true positives (TP), false positives (FP), and false negatives (FN). Additionally, for the length-based approach, we calculated true negatives (TN). For the segment-based approach, we used the F1-score metric (F1= 2*(precision*sensitivity)/(precision+sensitivity)) which is calculated based on sensitivity (correct positive predictions relative to total actual positives - TP/(TP+FN)) and precision (correct positive predictions relative to total positive predictions - TP/(TP+FP)), where 0 indicates no ROH overlap and 1 shows perfect overlap.

For the length-based approach we used the F1-score and the Mathews correlation coefficient (MCC), which takes into consideration all four confusion matrix categories (FN, FP, TN, TP), allowing equal contribution of positives and negatives. This is considered to be more reliable than the F1-score (39), as a high score is obtained when all four confusion matrix categories obtained good results (TP, TN, FP, FN), in comparison to the F1-score which primarily weighs the correct positive predictions. To calculate the MCC, we used the mcc function of mltools (40) in R. We then estimated the normalised Mathews correlation coefficient (nMCC=(MCC+1)/2), where a value equal to 0.5 indicates a random prediction and a value closer to 1 represents complete overlap. Finally, using the length-based overlap estimates, we calculated specificity (TN/(TN+FP)), sensitivity (TP/(TP+FN)) and false discovery rate (FDR=FP/(TP+FP)) on the total outcome.

ROH estimates across space and time

We estimated the total number and total length of ROH for each imputed individual, as well as the inbreeding coefficient (F_{ROH}), which is equal to the total length of ROH, divided by the total genome length. We estimated these metrics for both long (>=1.6Mb) and short (<1.6Mb) ROH blocks separately, since they can be indicative of different demographic events (41). We chose these cutoffs based on the distribution of ROH lengths calculated for ancient and modern dogs.

In order to visualise fluctuations in inbreeding patterns through space and time, we grouped the ancient dog samples into three geographic regions: Europe, the Arctic and the Near East (Dataset S1). ROH estimates of present-day dogs (breed dogs and village dogs) from these three regions were included for comparison. Within the Near Eastern cluster, we also included African and Indian village dogs, as well as African modern breeds. We also grouped

the imputed ancient wolves into three populations: Pleistocene, Holocene Eastern Eurasia and Holocene Western Eurasia. This grouping was based on previous work showing that Pleistocene wolves were a panmictic population and that population structure and differentiation increased during the Holocene (6). Present-day wolf samples from east and west Eurasia as well as from North America were included for comparison.

We subsequently carried out per population Mann–Whitney U tests using the wilcox.test function in R to test for significant differences between dog populations and time periods. For this we used the three geographic groupings (Europe, Arctic and Near East) and then carried out tests between each pairwise combination of: i) ancient dog populations, ii) present-day and ancient dog populations and iii) present-day dog populations. We carried out these tests on all, short and long ROH.

Prevalence of ROH in ancient and modern samples

To further characterise patterns of ROH presence and absence in ancient and present-day populations, we used the windowscanr v0.1 R package (https://github.com/tavareshugo/WindowScanR) to estimate the prevalence of ROH across the genome, following the published approach (42) (https://github.com/mastoffel/sheep_ID). We split the genome into 500 Kb windows and estimated the percentage of samples which contained a ROH in each window. This was carried out separately for the 50 ancient dogs, the 40 ancient wolves, a subset of present-day dogs (n=502) and a subset of present-day wolves (n=95) from the reference panel. We excluded windows with extremely high or low read depth, as they may be enriched for structural variants (e.g., copy number variants or segmental duplications) or mapping errors. We identified these outlier windows by estimating the average depth of coverage per window (n=4,385 total windows) using all ancient dog or wolf samples, and excluded all windows with a depth of coverage outside of two standard deviations from the mean. We also excluded windows which did not have any sites present in the imputed dataset. Based on these cutoffs, we retained 98% of the total windows for dogs (n=4,300) and 98.5% for wolves (n=4,323).

We defined ROH deserts as windows in which <5% of the ancient samples and <5% of the present-day samples had an ROH. This resulted in 124 ROH deserts for dogs and 209 for wolves. In order to control for the presence of CNVs, we applied the following filtering approach. We utilised a dataset of per sample read depth of 1,824 dogs and 55 wolves over 2,040,588 autosomal windows from the Dog10K consortium (43). We extracted each autosomal window that had a copy number deviating from the expected diploid state (>2 or <2) in a sample. We refer to these windows as CNV windows. We used liftover to convert the coordinates of the CNV windows from CanFam4

to CanFam3.1 using the liftover chain available at https://hgdownload.soe.ucsc.edu/goldenPath/canFam4/liftOver/canFam4ToCanFam3.over.chain.gz, only keeping regions which were assigned to autosomes. We then estimated the frequency of each CNV window in the 1,824 dogs and 55 wolves. We then intersected the CNV windows with our 4,385 500Kb autosomal windows used in the ROH analysis, using the bedtools intersect function. From the intersect output, we estimated the weighted mean CNV frequency for each 500Kb window:

*Weighted mean CNV frequency = sum(CNV frequency * (CNV overlap / total CNV overlap))*

The distribution of this metric across dogs and wolves is shown in Fig. S68. We then looked at the correlation between mean ROH prevalence and the weighted mean frequency of CNV windows across our ROH deserts, which we show in Fig. S69 (Dogs: p=0.0282, r^2=0.039; Wolves: p=0.00017, r^2=0.07). We estimated the CNV frequency cutoff under which the correlation between the two measures was no longer statistically significant, 0.13 for modern dogs and 0.19 for wolves, and removed any of our ROH deserts surpassing those thresholds (Fig. S69) (Dogs: p=0.06, r^2=0.038; Wolves: p=0.09, r^2=0.017). This resulted in removing 31 out of the 124 ROH deserts for dogs and 30 out of the 209 ROH deserts for wolves. We additionally removed any ROH deserts located in telomeric regions by excluding the first or last 500Kb window of each chromosome. This resulted in a final set of 87 ROH desert windows for dogs and 171 for wolves.

We subsequently carried out GO enrichment analysis on the filtered ROH deserts using the GOfuncR package (44) to test for an over-representation of genes related to specific biological categories among the genes that fell within ROH deserts. To this end, we applied the hypergeometric test for GO enrichment, correcting for gene length. From the background, we removed windows with extremely high or low read depth as well as windows located in the first or last 500Kb of each chromosome We used the 'org.Cf.eg.db' OrgDb package for GO-annotations and the 'TxDb.Cfamiliaris.UCSC.canFam3.refGene' TxDb package for gene-coordinates. To correct for multiple testing and test interdependency, we computed the family-wise error rate (FWER) for each GO-category, using 1000 randomised sets of the data. In each randomised set, the background and candidate genes are permuted, and new p-values are computed. For a given GO-category, the FWER is then the fraction of the randomised sets whose lowest p-value is lower than or equal to the original p-value of the GO-category. For example, a FWER of 0.1 for a GO-category “X”

means that, in 10 out of 1000 randomised sets of the data, the set's minimum p-value is smaller than or equal to the original p-value of "X" (see GOfuncR's online manual for an extended explanation). We also reran the GO enrichment analysis masking the DLA regions as previously identified in (45): chr12:307,171-2,872,051, chr18:41,142,496-41,145,658, and chr35:25,514,060-26,406,861, to test whether the immune signal persists or if it's driven solely by the DLA region.

Randomization procedure to assess the statistical significance of ROH desert sharing

To assess the statistical significance of the sharing of ROH deserts between ancient and present-day dogs, relative to a null ROH placement model, we implemented a custom randomization procedure, which is available as a fully reproducible R project in this GitHub repository: https://github.com/bodkan/dogs-randomization.

The procedure begins with a data set of the ROH overlap status for each locus in every individual, i.e. if a site in the genome of an individual overlaps an ROH segment, the site is scored as 1 (`TRUE`), otherwise it carries the value of 0 (`FALSE`). Additionally, each site is assigned the index of one of the 4,300 genomic windows (each window 500 kb long) whose range it falls into. In each iteration of the randomization, we reshuffled the windows in every individual so that the sites (and, specifically, their `TRUE` or `FALSE` ROH status in that particular individual) appeared in random positions in the genome, yet still remained together with other sites within their original window. Having reshuffled genomic windows in all individuals in a given iteration, we then determined the ROH desert status of every window across all reshuffled genomes according to the procedure described in the previous section, and counted the number of ROH desert windows shared between ancient and present-day dogs. The entire randomization loop was repeated for 1,000 iterations. In the final step, we computed a one-sided p-value for the observed number of shared deserts (n = 124) by comparing it directly to the distribution of counts obtained from 1,000 randomized replicates. Specifically, the p-value was defined as the proportion of replicates in which the number of shared deserts was greater than or equal to the observed count. None of the replicates reached or exceeded the observed value (Fig. S70).

Supplementary Figures

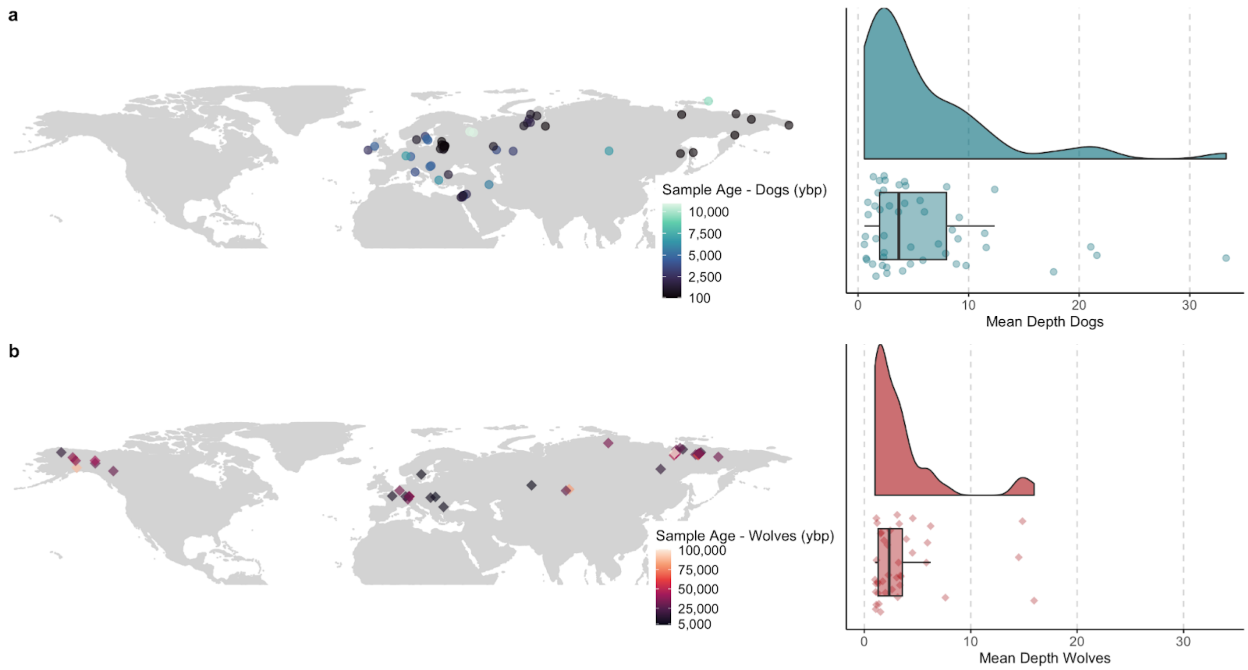

Fig. S1: *Map of ancient a) dog (>0.5x) and b) wolf samples (>1x) used in this study, along with the distributions of mean depth for each.*

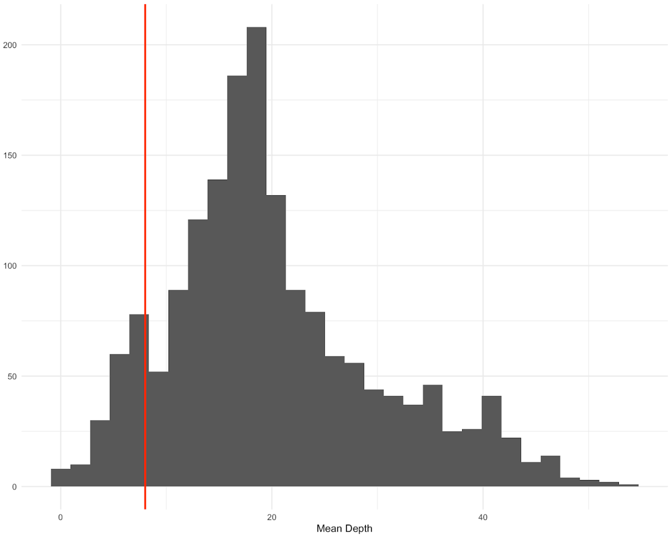

Fig. S2: *Histogram showing the mean depth for all genomes within the 1,715 sample VCF. The red line indicates the 8x cutoff we applied for including a sample in the reference panel. Two samples with very high coverage (>300x) were excluded from the plot for visualisation purposes.*

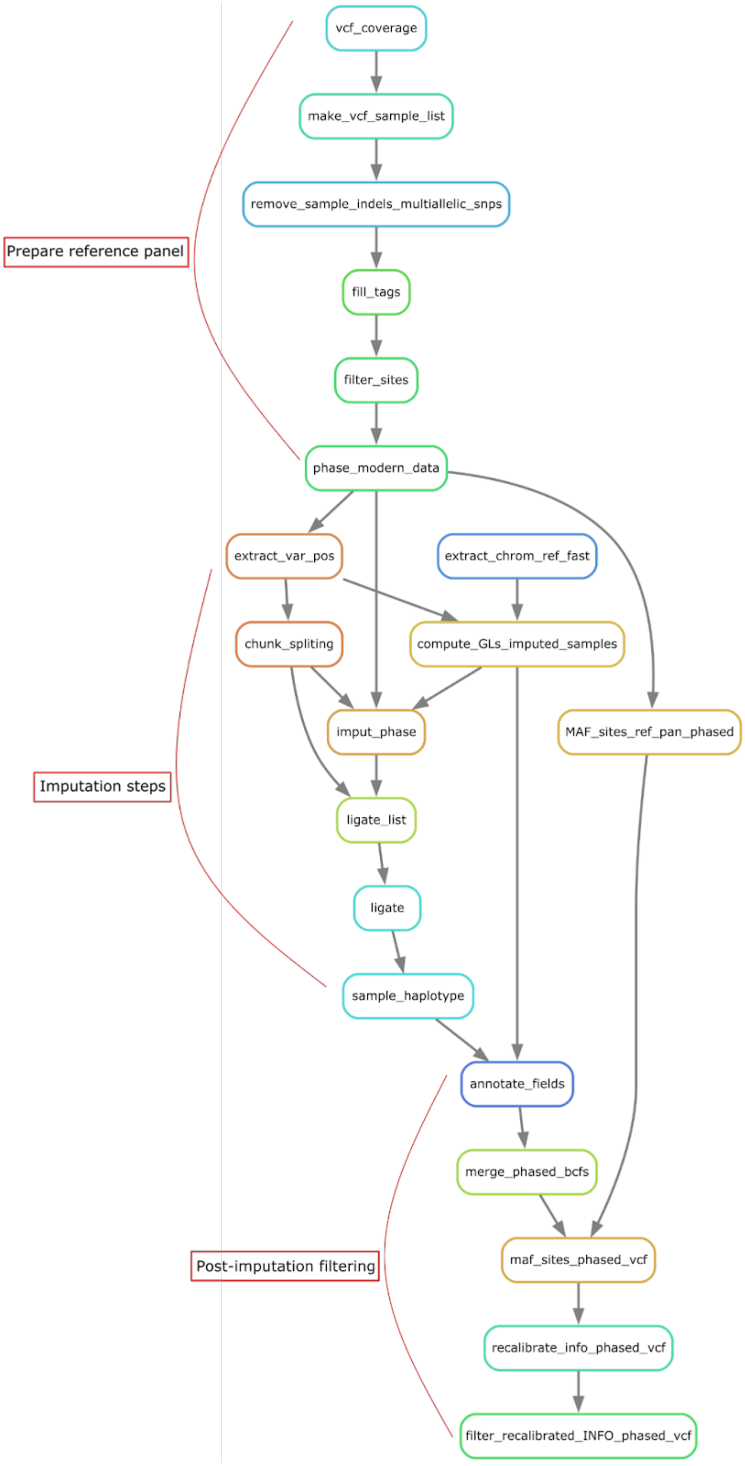

Fig. S3: *Snakemake rulegraph of the imputation pipeline used for this study partitioned into three sections: 1) Filtering and phasing of the reference panel, 2) imputation and phasing of ancient samples and 3) post imputation filtering by applying MAF and INFO score cutoffs.*

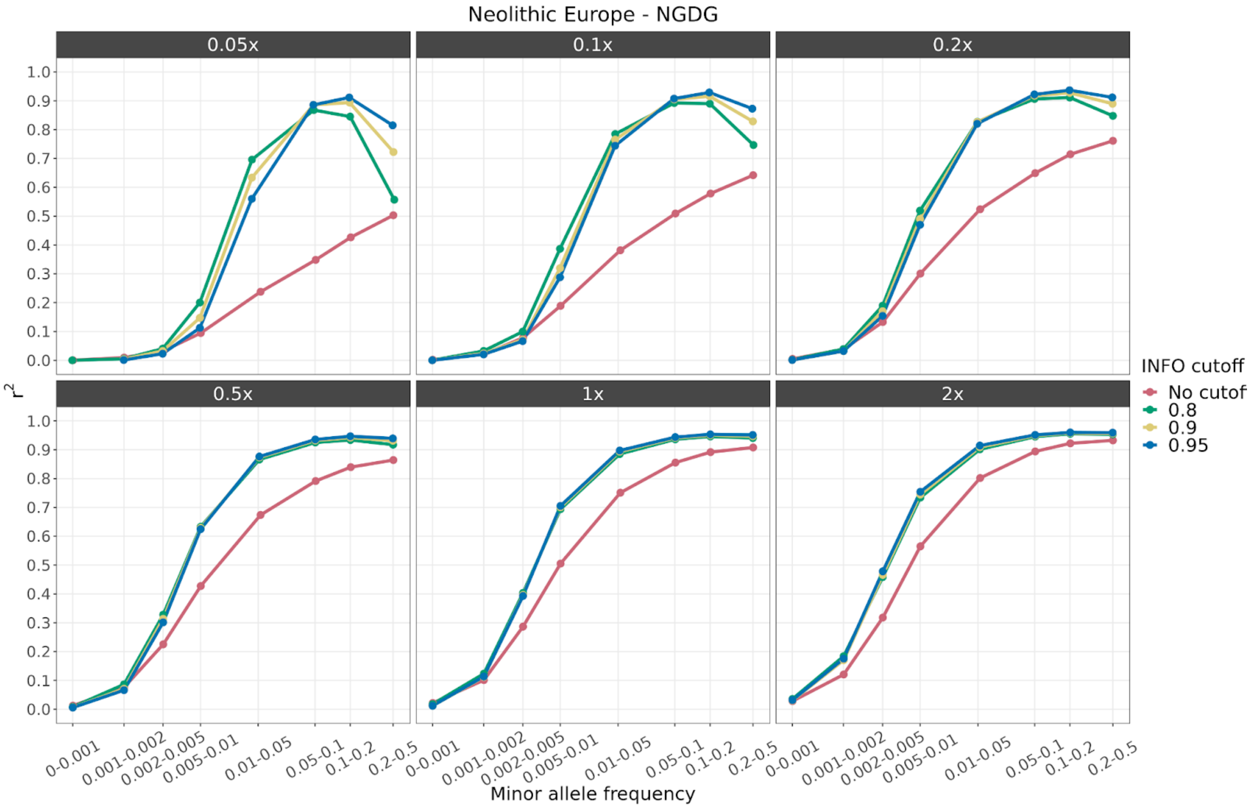

Fig. S4: *Squared correlation between imputed genotypes by GLIMPSE and highly confident called genotypes for the Newgrange Neolithic European dog, downsampled to six coverage values (0.01x, 0.05, 0.1x, 0.5x, 1x and 2x) and across different MAF bins. All target samples were imputed using the reference panel containing all canids. Each colour depicts the accuracy for a given INFO score cutoff. Red: no cut-off, Green: 0.8, Yellow: 0.9 and Blue: 0.95.*

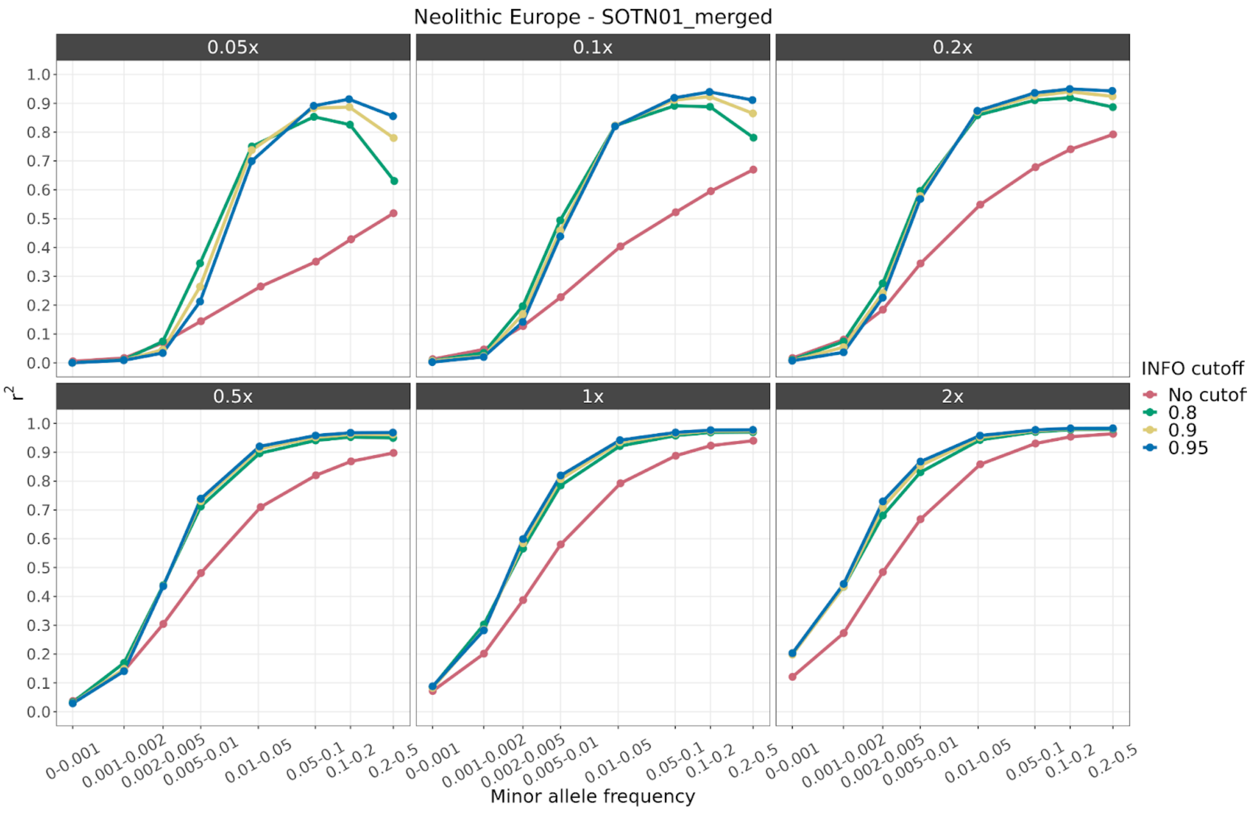

Fig. S5: *Squared correlation between imputed genotypes by GLIMPSE and highly confident called genotypes for the SOTN01 Neolithic European dog, downsampled to six coverage values (0.01x, 0.05, 0.1x, 0.5x, 1x and 2x) and across different MAF bins. All target samples were imputed using the reference panel containing all canids. Each colour depicts the accuracy for a given INFO score cutoff. Red: no cut-off, Green: 0.8, Yellow: 0.9 and Blue: 0.95.*

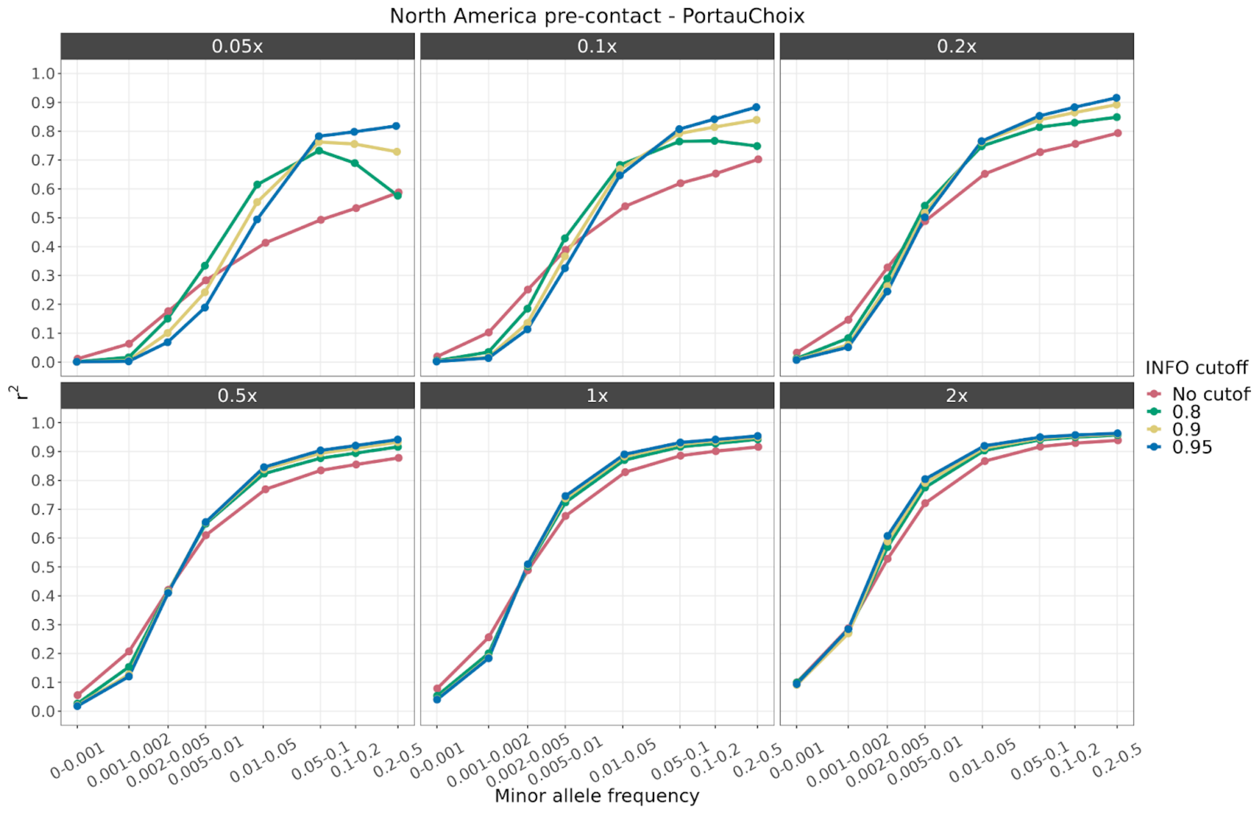

Fig. S6: *Squared correlation between imputed genotypes by GLIMPSE and highly confident called genotypes for the Port au Choix North American pre-contact dog, downsampled to six coverage values (0.01x, 0.05, 0.1x, 0.5x, 1x and 2x) and across different MAF bins. All target samples were imputed using the reference panel containing all canids. Each colour depicts the accuracy for a given INFO score cutoff. Red: no cut-off, Green: 0.8, Yellow: 0.9 and Blue: 0.95.*

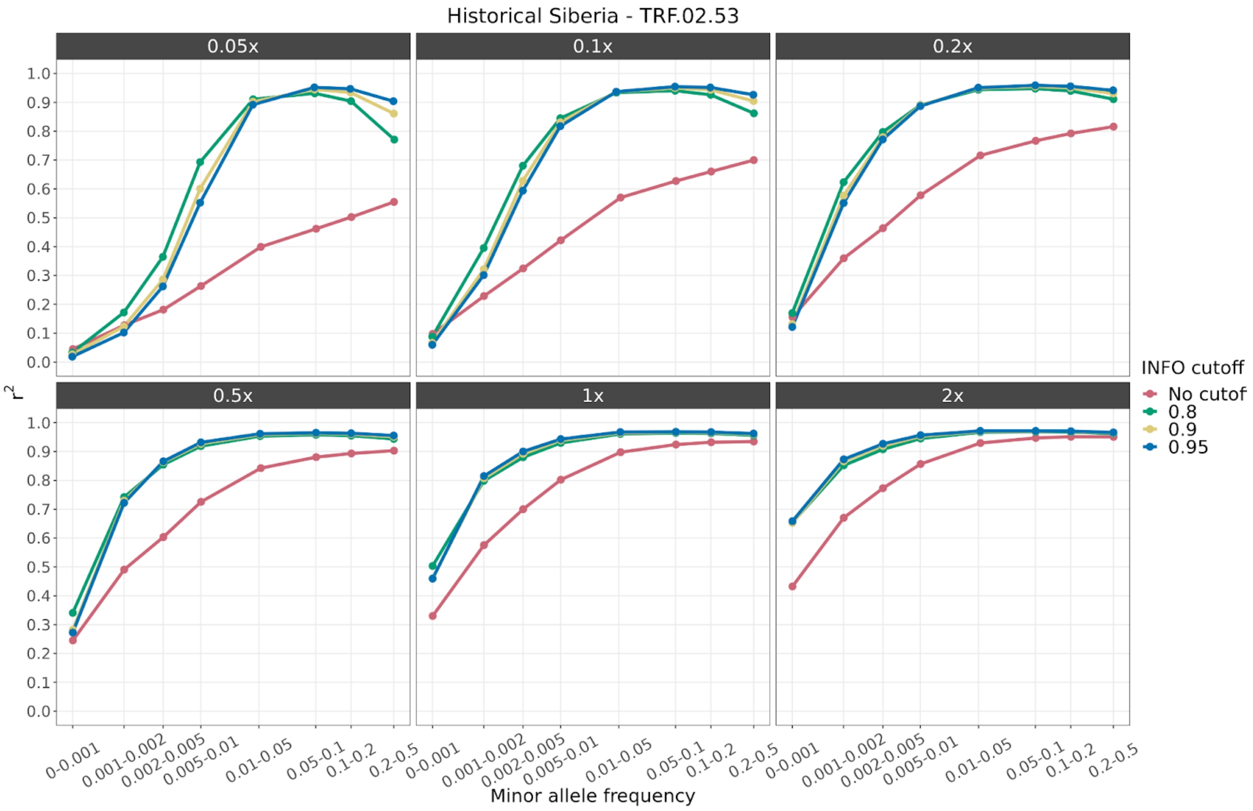

Fig. S7: *Squared correlation between imputed genotypes by GLIMPSE and highly confident called genotypes for the TRF.02.53 historical Siberian dog, downsampled to six coverage values (0.01x, 0.05, 0.1x, 0.5x, 1x and 2x) and across different MAF bins. All target samples were imputed using the reference panel containing all canids. Each colour depicts the accuracy for a given INFO score cutoff. Red: no cut-off, Green: 0.8, Yellow: 0.9 and Blue: 0.95.*

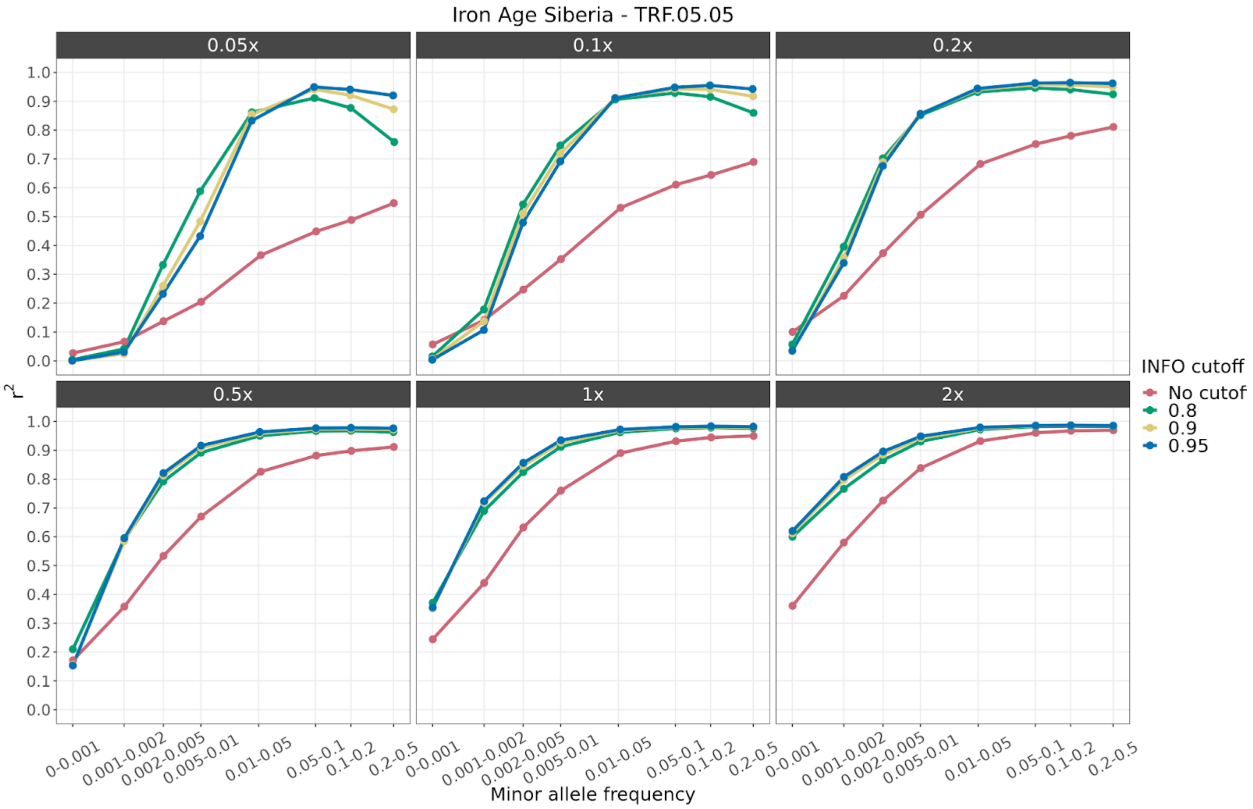

Fig. S8: *Squared correlation between imputed genotypes by GLIMPSE and highly confident called genotypes for the TRF.05.05 Iron Age Siberian dog, downsampled to six coverage values (0.01x, 0.05, 0.1x, 0.5x, 1x and 2x) and across different MAF bins. All target samples were imputed using the reference panel containing all canids. Each colour depicts the accuracy for a given INFO score cutoff. Red: no cut-off, Green: 0.8, Yellow: 0.9 and Blue: 0.95.*

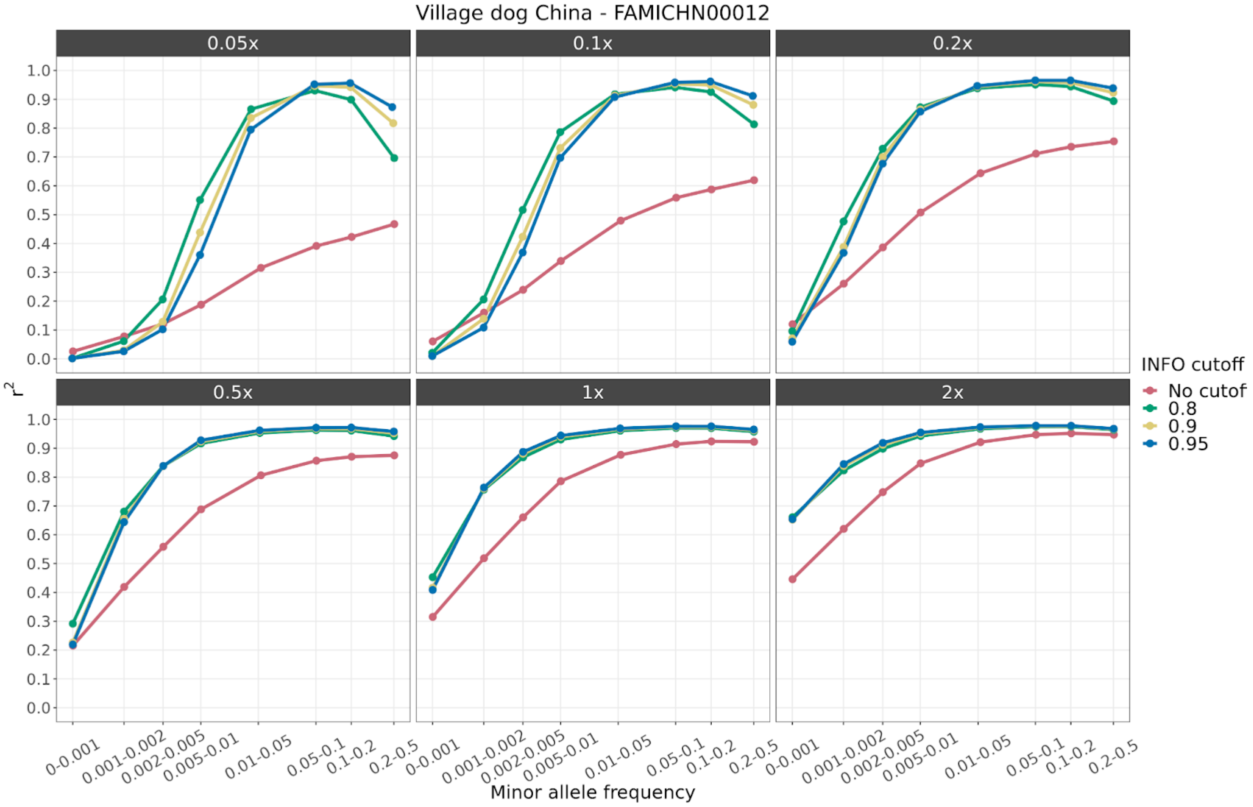

Fig. S9: *Squared correlation between imputed genotypes by GLIMPSE and highly confident called genotypes for the FAMICHN00012 Chinese Village dog, downsampled to six coverage values (0.01x, 0.05, 0.1x, 0.5x, 1x and 2x) and across different MAF bins. All target samples were imputed using the reference panel containing all canids. Each colour depicts the accuracy for a given INFO score cutoff. Red: no cut-off, Green: 0.8, Yellow: 0.9 and Blue: 0.95.*

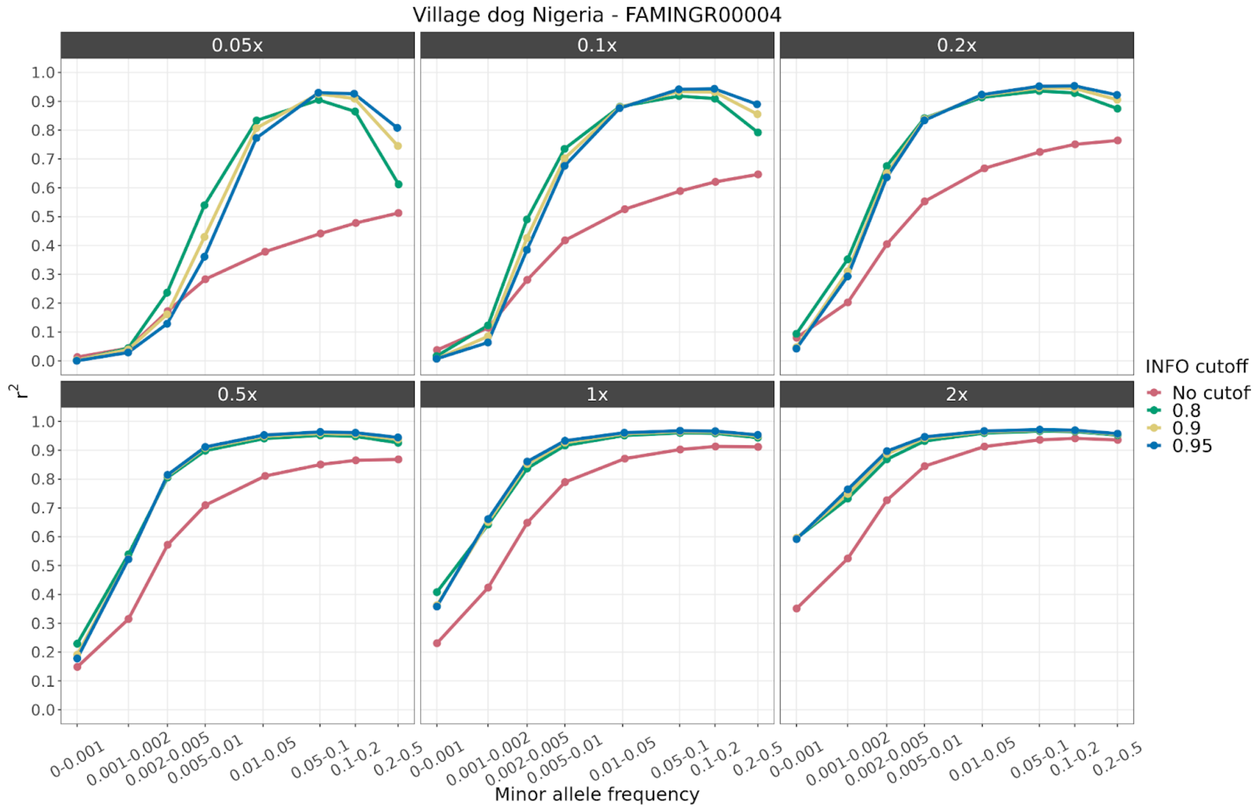

Fig. S10: *Squared correlation between imputed genotypes by GLIMPSE and highly confident called genotypes for the FAMINGR00004 Nigerian Village dog, downsampled to six coverage values (0.01x, 0.05, 0.1x, 0.5x, 1x and 2x) and across different MAF bins. All target samples were imputed using the reference panel containing all canids. Each colour depicts the accuracy for a given INFO score cutoff. Red: no cut-off, Green: 0.8, Yellow: 0.9 and Blue: 0.95.*

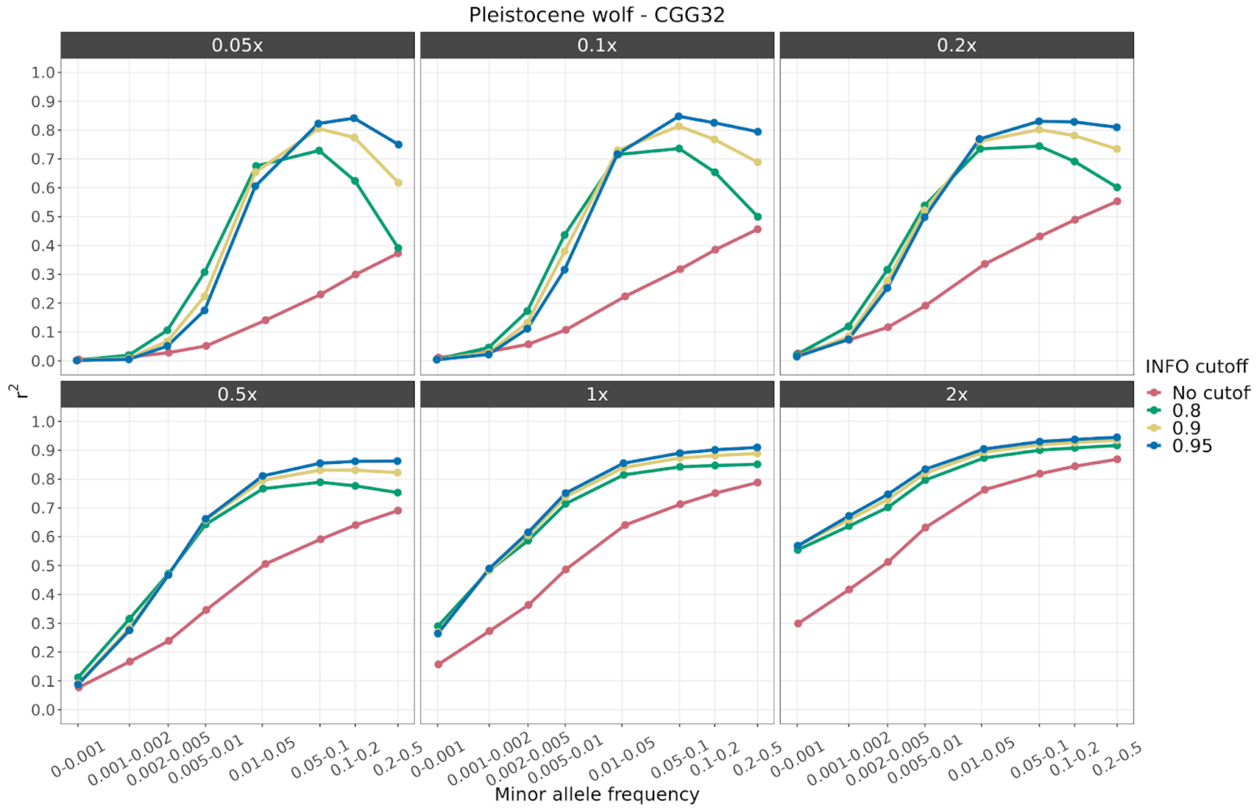

Fig. S11: *Squared correlation between imputed genotypes by GLIMPSE and highly confident called genotypes for the CGG32 Pleistocene wolf, downsampled to six coverage values (0.01x, 0.05, 0.1x, 0.5x, 1x and 2x) and across different MAF bins. All target samples were imputed using the reference panel containing all canids. Each colour depicts the accuracy for a given INFO score cutoff. Red: no cut-off, Green: 0.8, Yellow: 0.9 and Blue: 0.95.*

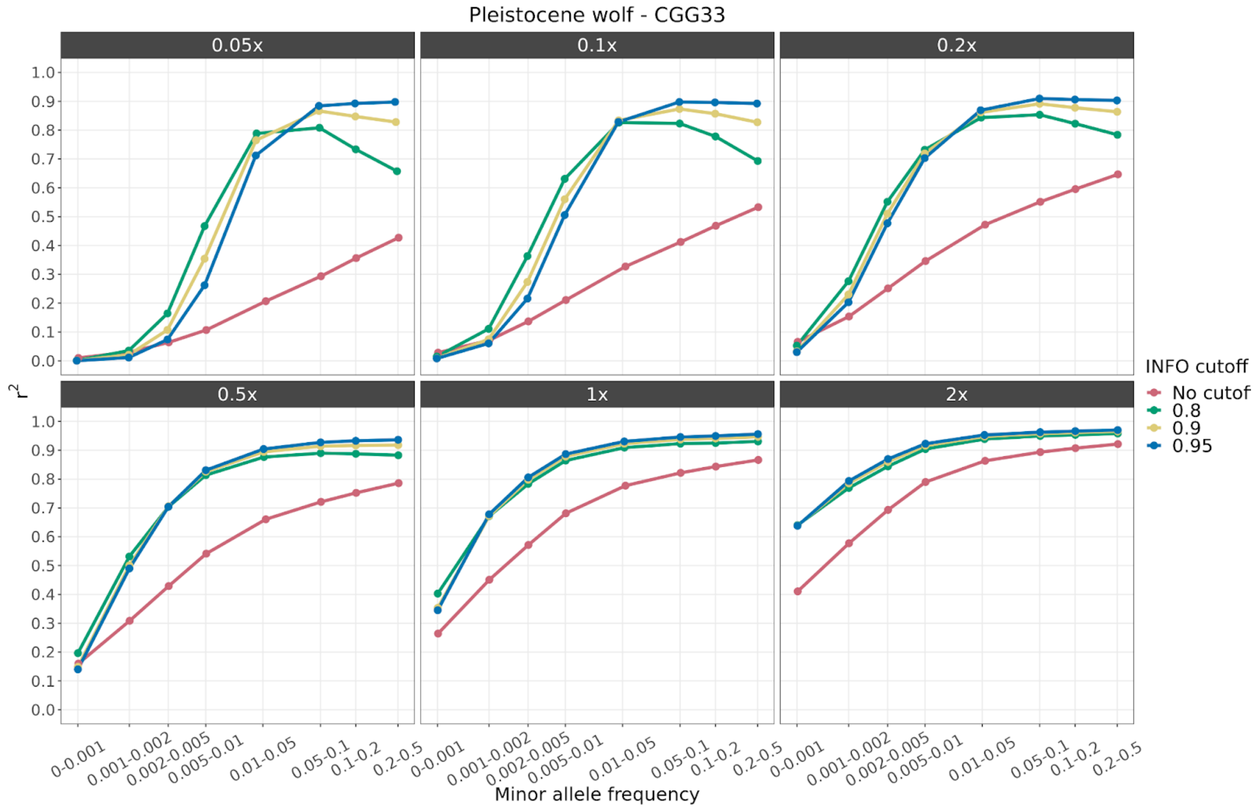

Fig. S12: *Squared correlation between imputed genotypes by GLIMPSE and highly confident called genotypes for the CGG33 Pleistocene wolf, downsampled to six coverage values (0.01x, 0.05, 0.1x, 0.5x, 1x and 2x) and across different MAF bins. All target samples were imputed using the reference panel containing all canids. Each colour depicts the accuracy for a given INFO score cutoff. Red: no cut-off, Green: 0.8, Yellow: 0.9 and Blue: 0.95.*

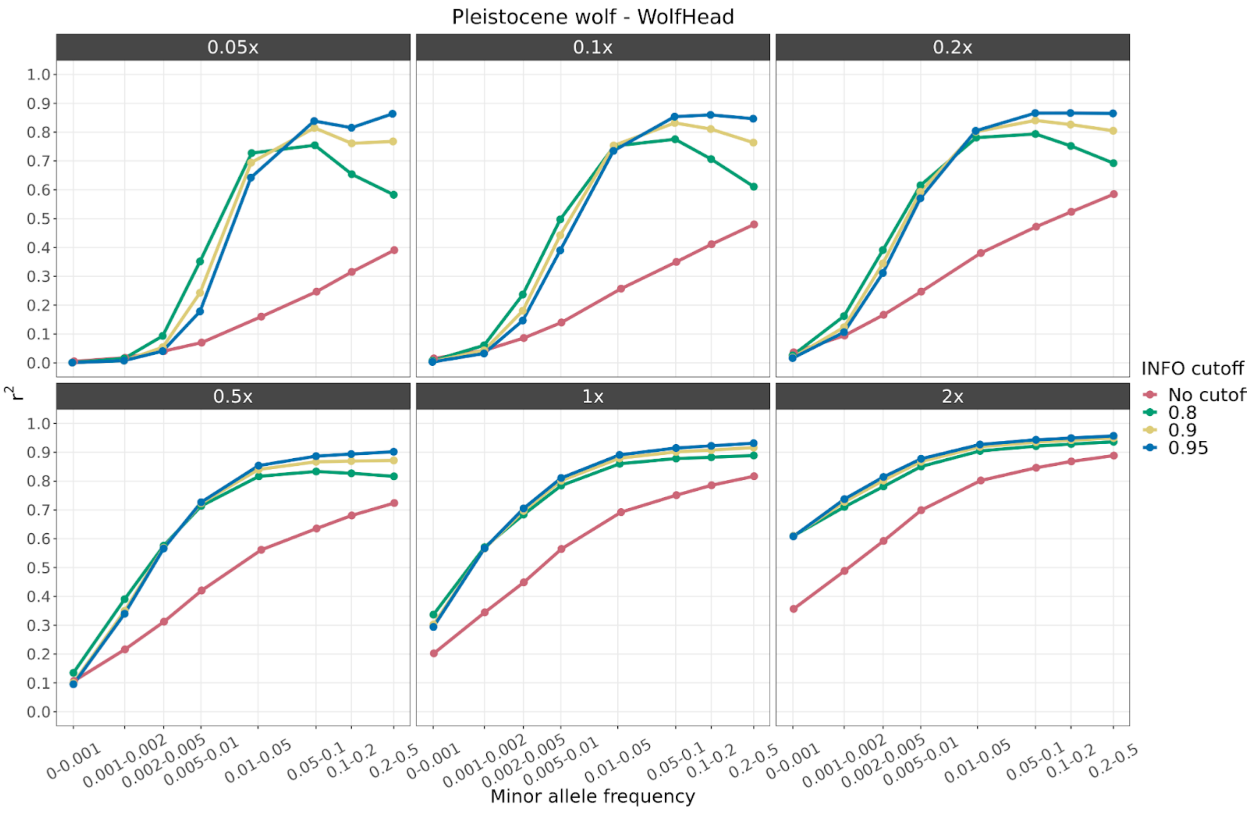

Fig. S13: *Squared correlation between imputed genotypes by GLIMPSE and highly confident called genotypes for the WolfHead Pleistocene wolf, downsampled to six coverage values (0.01x, 0.05, 0.1x, 0.5x, 1x and 2x) and across different MAF bins. All target samples were imputed using the reference panel containing all canids. Each colour depicts the accuracy for a given INFO score cutoff. Red: no cut-off, Green: 0.8, Yellow: 0.9 and Blue: 0.95.*

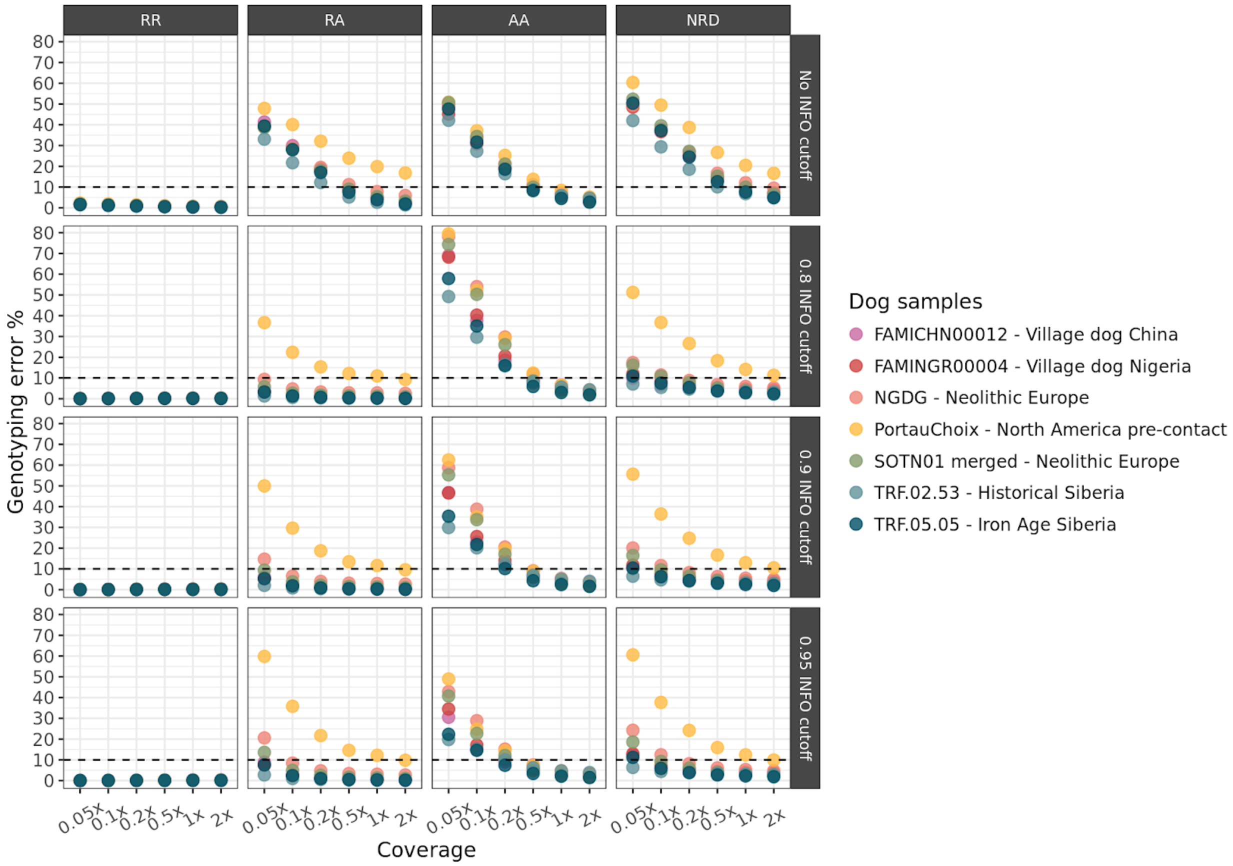

Fig. S14: *Genotyping error between imputed downsampled and the high coverage target dog samples for homozygous alternative (AA), heterozygous (RA) and homozygous reference alleles (RR), and the non-reference discordance (NRD) metric estimated by GLIMPSE concordance for all autosomes. Comparisons are shown for different INFO score cutoffs (no cutoff, 0.8, 0.9 and 0.95).*

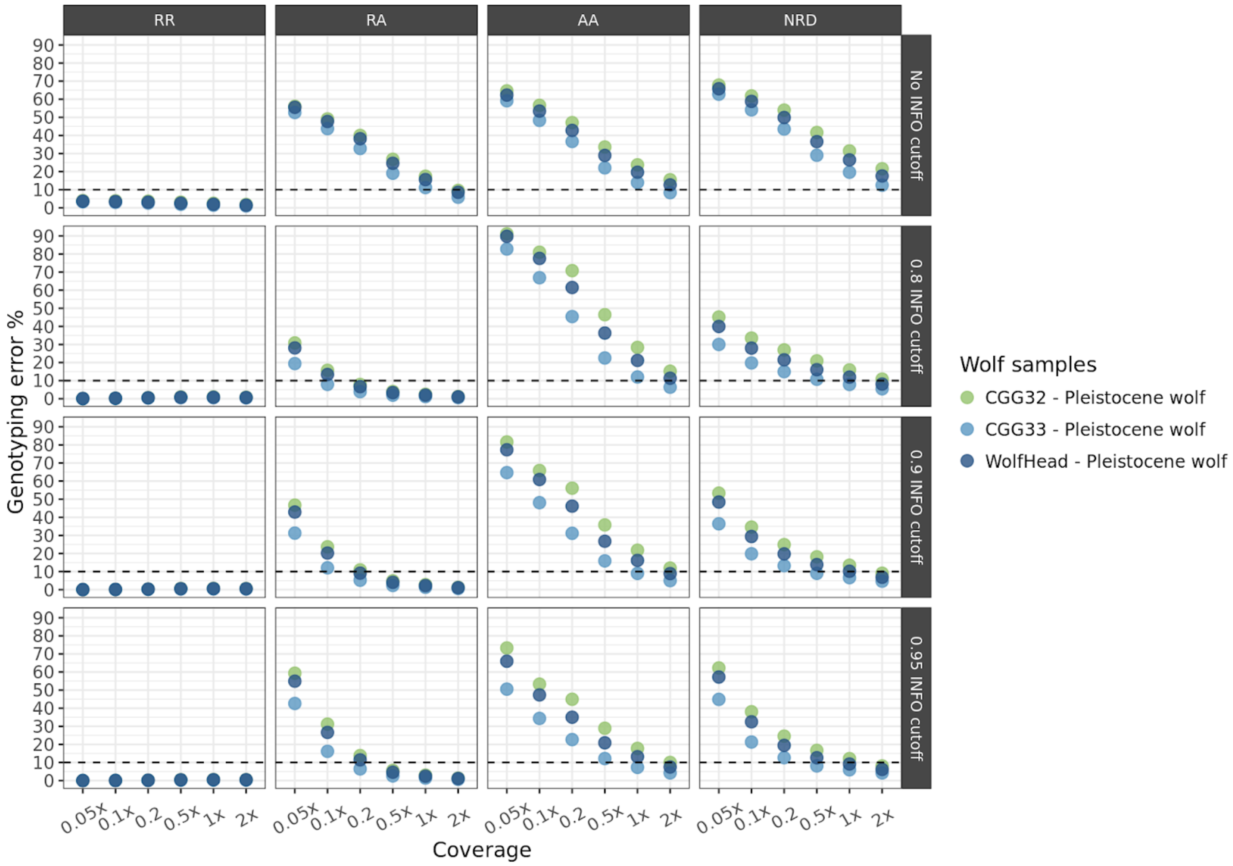

Fig. S15: *Genotyping error between imputed downsampled and the high coverage target Pleistocene wolf samples for homozygous alternative (AA), heterozygous (RA) and homozygous reference alleles (RR), and the non-reference discordance (NRD) metric estimated by GLIMPSE concordance for all autosomes. Comparisons are shown for different INFO score cutoffs (no cutoff, 0.8, 0.9 and 0.95).*

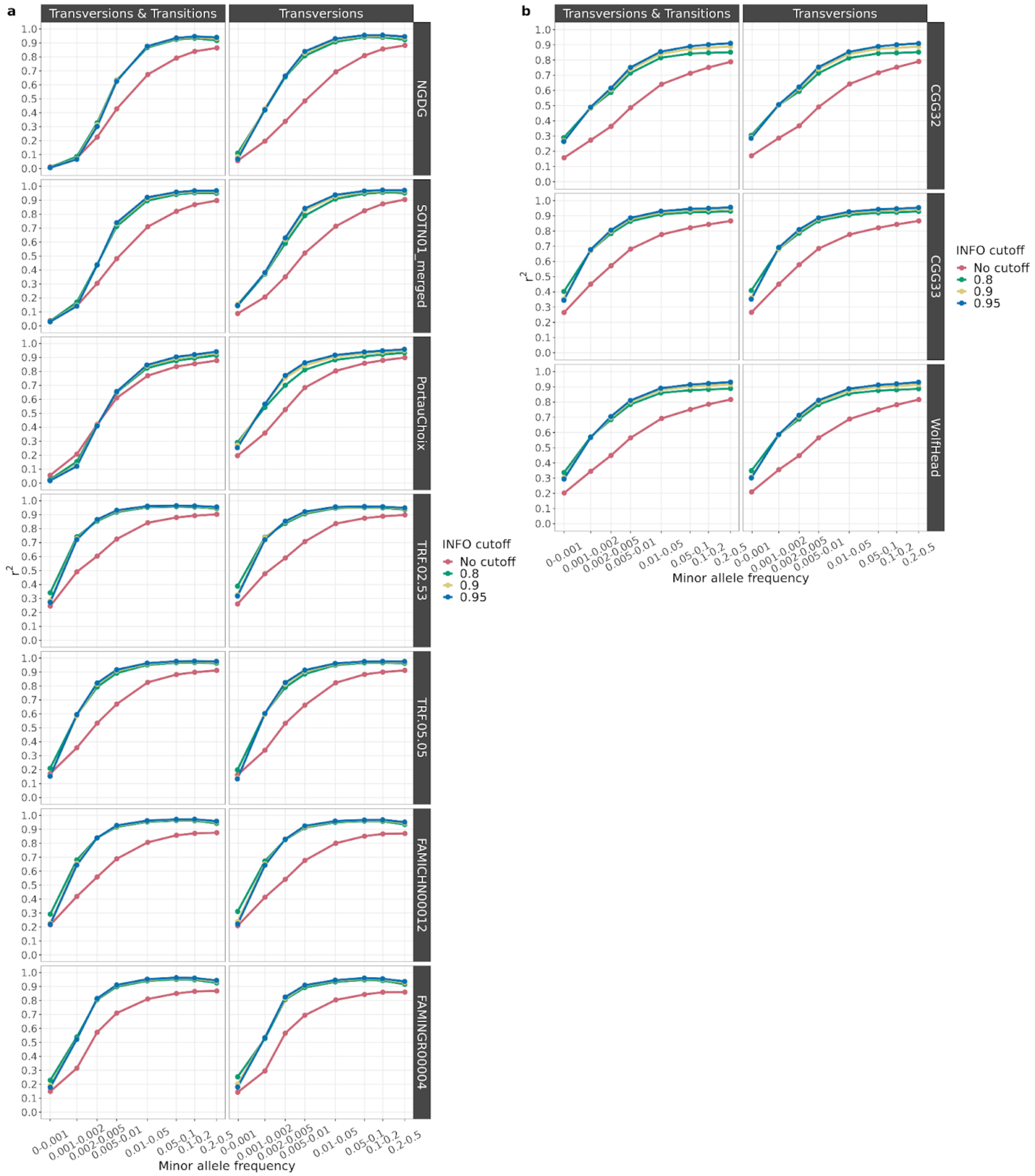

Fig. S16: *Squared correlation between imputed genotypes by GLIMPSE and highly confident called genotypes for all 10 target samples, downsampled to a) 0.5x for dogs and b) 1x for wolves and across different MAF bins using either transversions and transitions or only transversions. Each colour depicts the accuracy for a given INFO score cutoff. Red: no cut-off, Green: 0.8, Yellow: 0.9 and Blue: 0.95.*

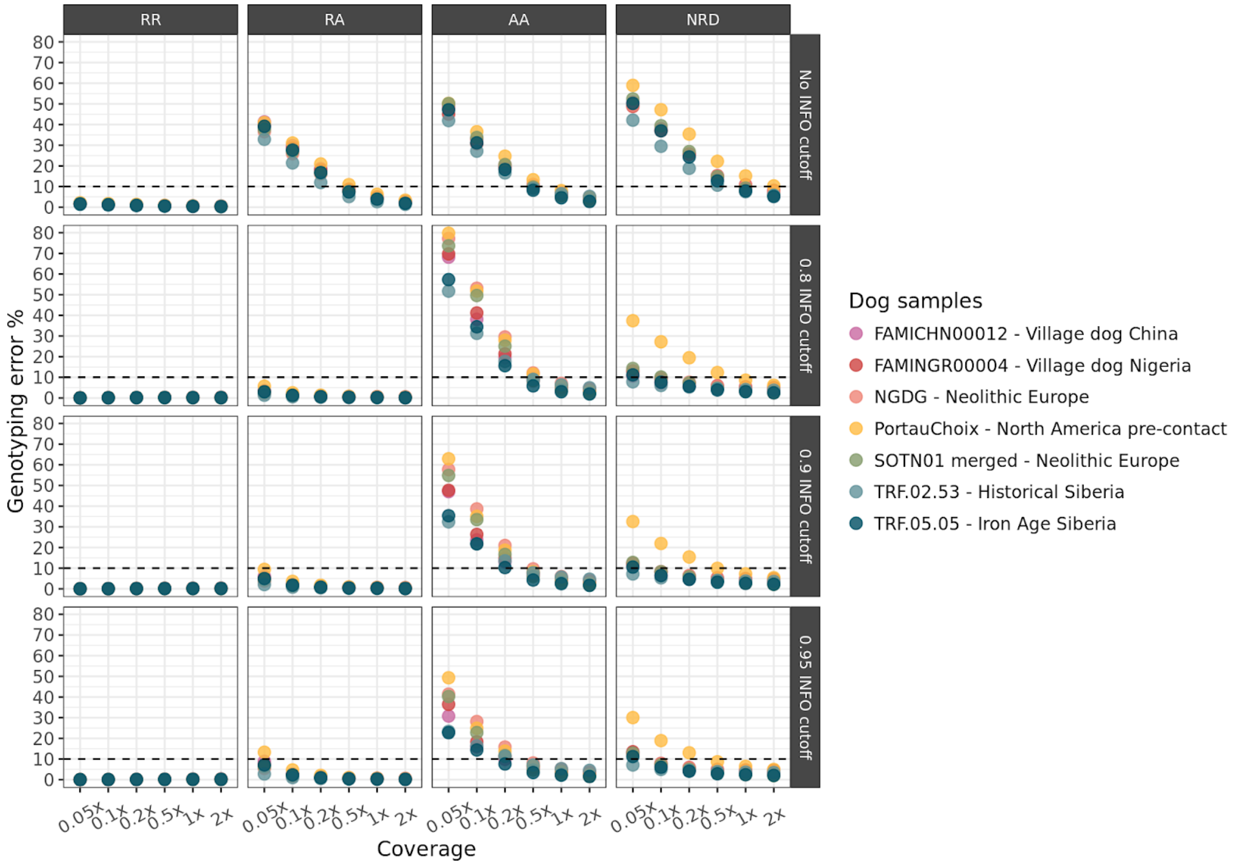

Fig. S17: *Genotyping error between imputed downsampled and the high coverage target dog samples using only transversions for homozygous alternative (AA), heterozygous (RA) and homozygous reference alleles (RR), and the non-reference discordance (NRD) metric estimated by GLIMPSE concordance for all autosomes. Comparisons are shown for different INFO score cutoffs (no cutoff, 0.8, 0.9 and 0.95).*

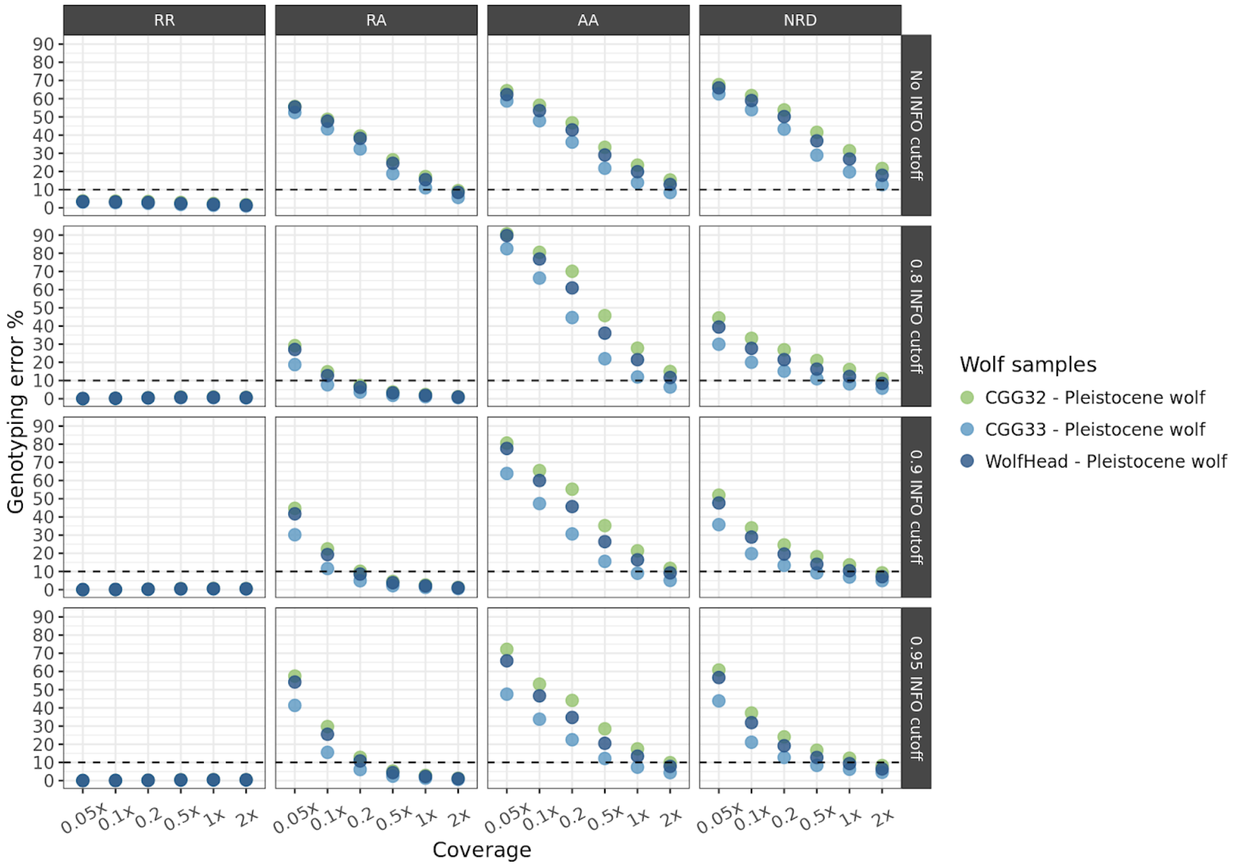

Fig. S18: *Genotyping error between imputed downsampled and the high coverage target Pleistocene wolf samples using only transversions for homozygous alternative (AA), heterozygous (RA) and homozygous reference alleles (RR), and the non-reference discordance (NRD) metric estimated by GLIMPSE concordance for all autosomes. Comparisons are shown for different INFO score cutoffs (no cutoff, 0.8, 0.9 and 0.95).*

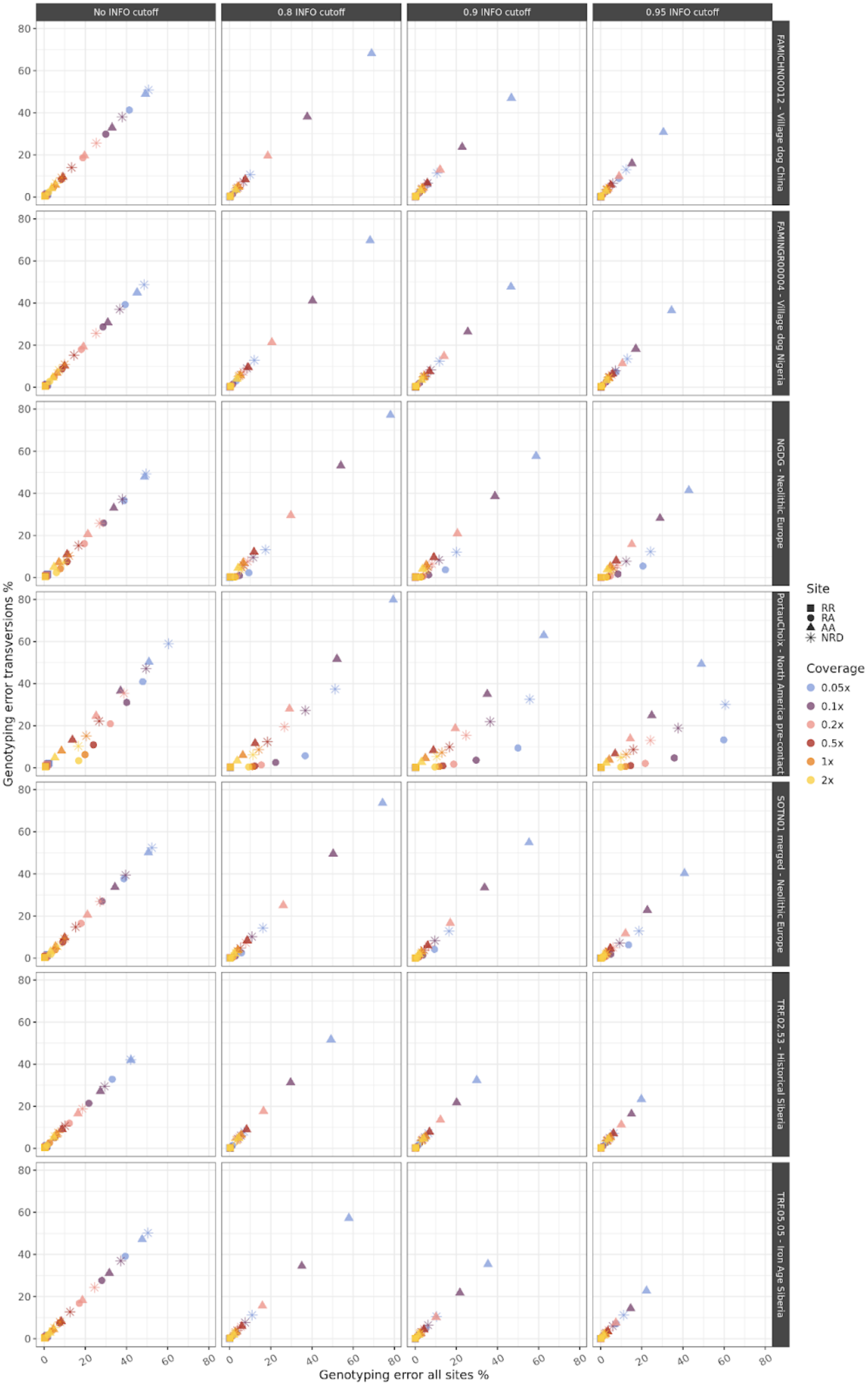

No INFO cutoff
0.8 INFO cutoff
0.9 INFO cutoff
0.95 INFO cutoff
FAMICHN00012 - Village dog China
FAMINGR00004 - Village dog Nigeria
NGDG - Neolithic Europe
PortauChoix - North America pre-contact
SOTN01 merged - Neolithic Europe
TRF.02.53 - Historical Siberia
TRF.05.05 - Iron Age Siberia
Genotyping error transversions %
Genotyping error all sites %
Site
RR
RA
AA
NRD
Coverage
0.05x
0.1x
0.2x
0.5x
1x
2x

Fig. S19: *Genotyping error of transversions and transitions (all sites) against genotyping error of transversions for the seven imputed target dog samples (rows) applying different INFO score cutoffs (columns). Estimates are shown for genotyping errors of homozygous reference (RR), heterozygous (RA), homozygous alternative (AA) alleles and the non-reference discordance (NRD) metric. Colours represent different coverages.*

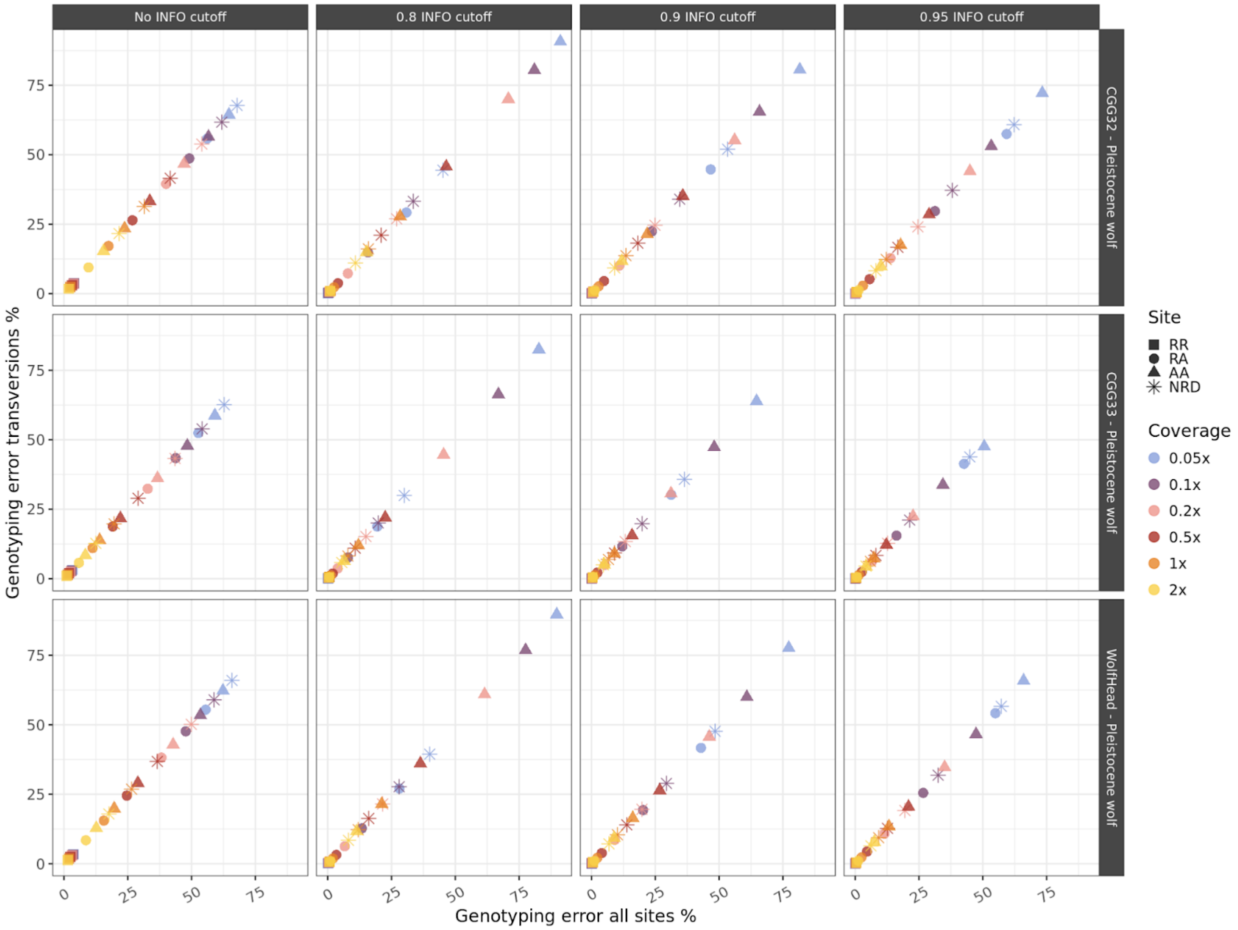

Fig. S20: *Genotyping error of transversions and transitions (all sites) against genotyping error of transversions for the three imputed target wolf samples (rows) applying different INFO score cutoffs (columns). Estimates are shown for genotyping errors of homozygous reference (RR), heterozygous (RA), homozygous alternative (AA) alleles and the non-reference discordance (NRD) metric. Colours represent different coverages.*

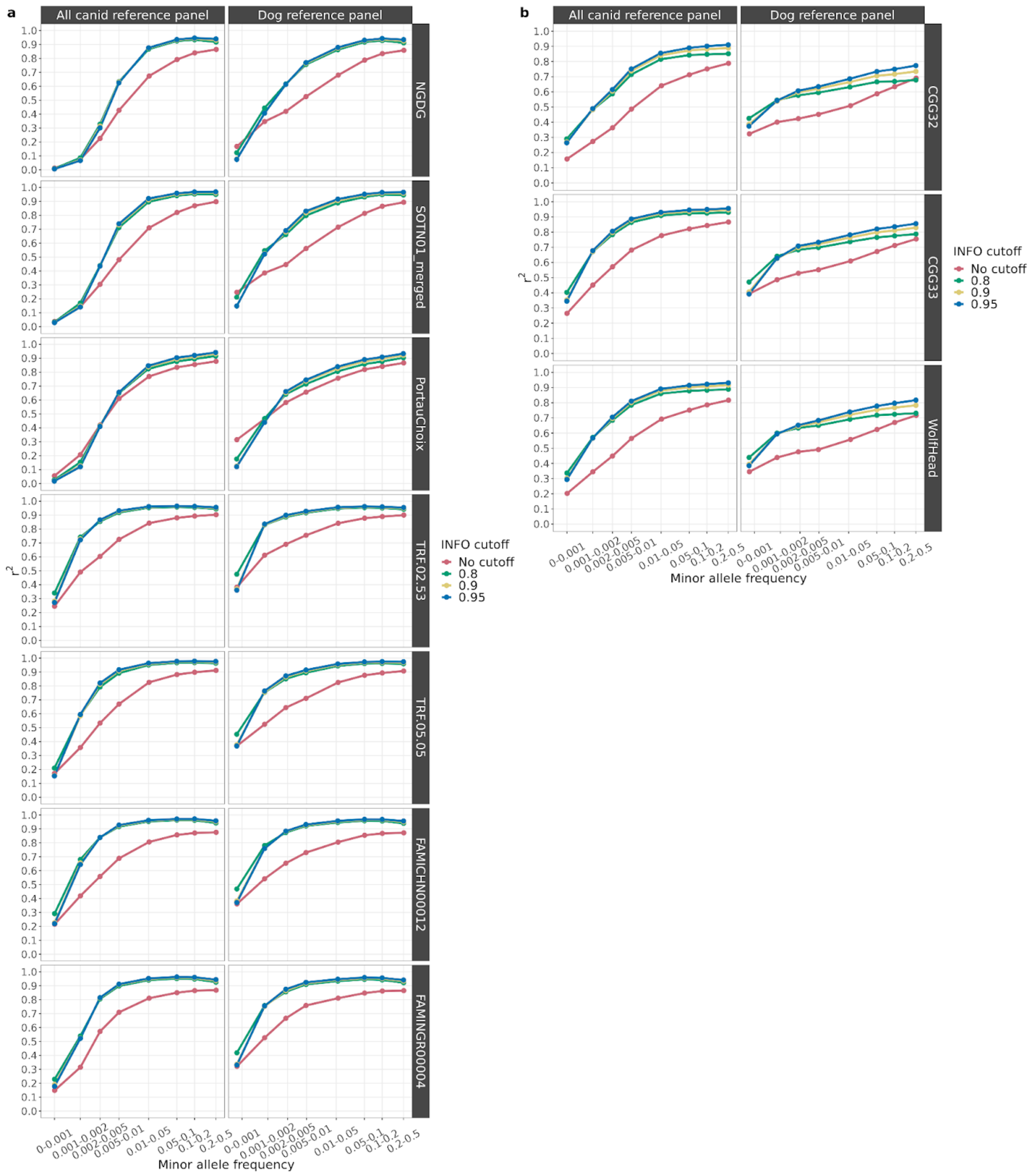

Fig. S21: *Squared correlation between imputed genotypes by GLIMPSE and highly confident called genotypes for all 10 target samples, downsampled to a) 0.5x for dogs and b) 1x for wolves and across different MAF bins using either the all canid reference panel or the reference panel containing only dogs. Each colour depicts the accuracy for a given INFO score cutoff. Red: no cut-off, Green: 0.8, Yellow: 0.9 and Blue: 0.95.*

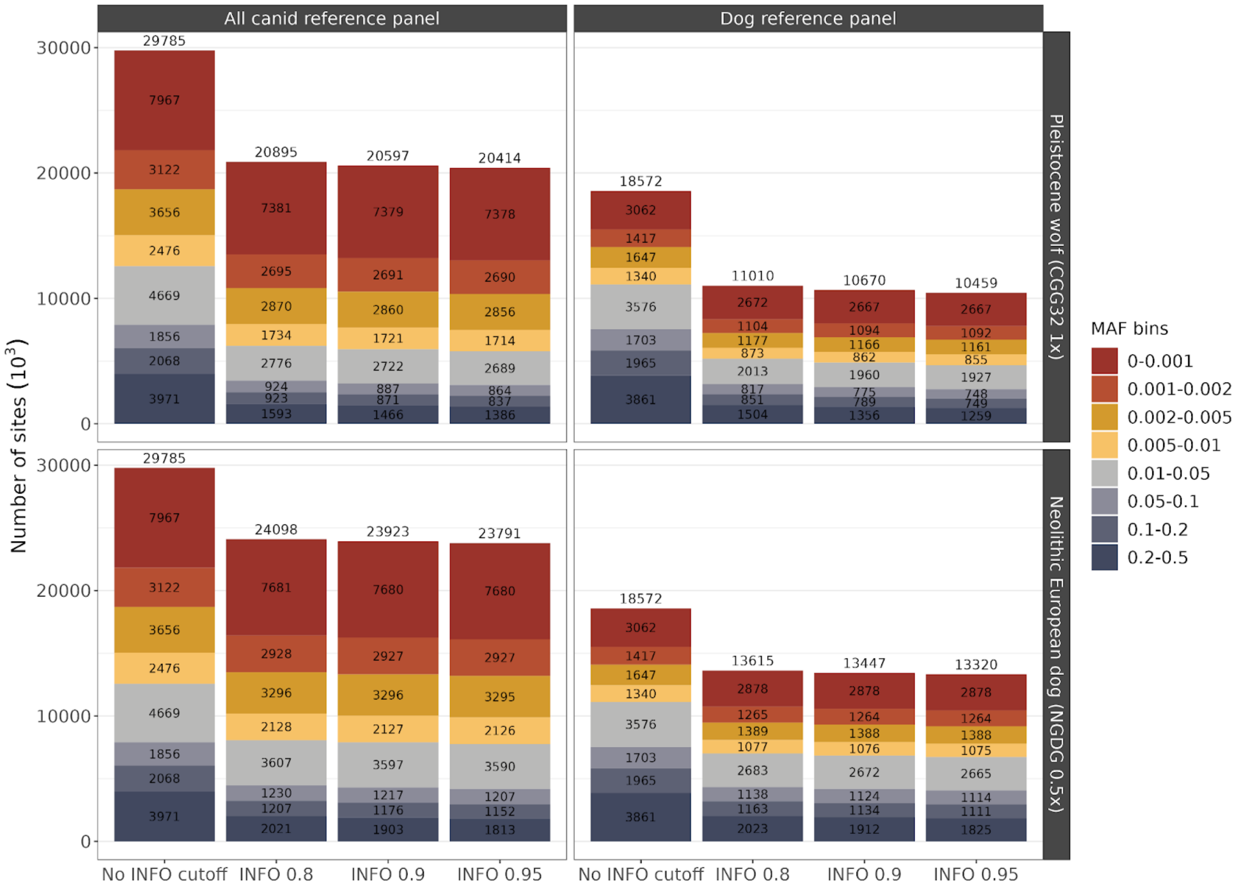

Fig. S22: *Number of imputed sites retained after applying INFO score cutoffs (no cutoff, >0.8, >0.9 and >0.95) across different minor allele frequency (MAF) bins for two imputed target samples (Dog: NGDG 0.5x, Wolf: CGG32 1x) using an all canid and a dog only reference panel. Numbers in each subgroup correspond to the number of sites (10^3) within that MAF bin. Numbers on top of each bar are the total number of sites (10^3) across all MAF bins.*

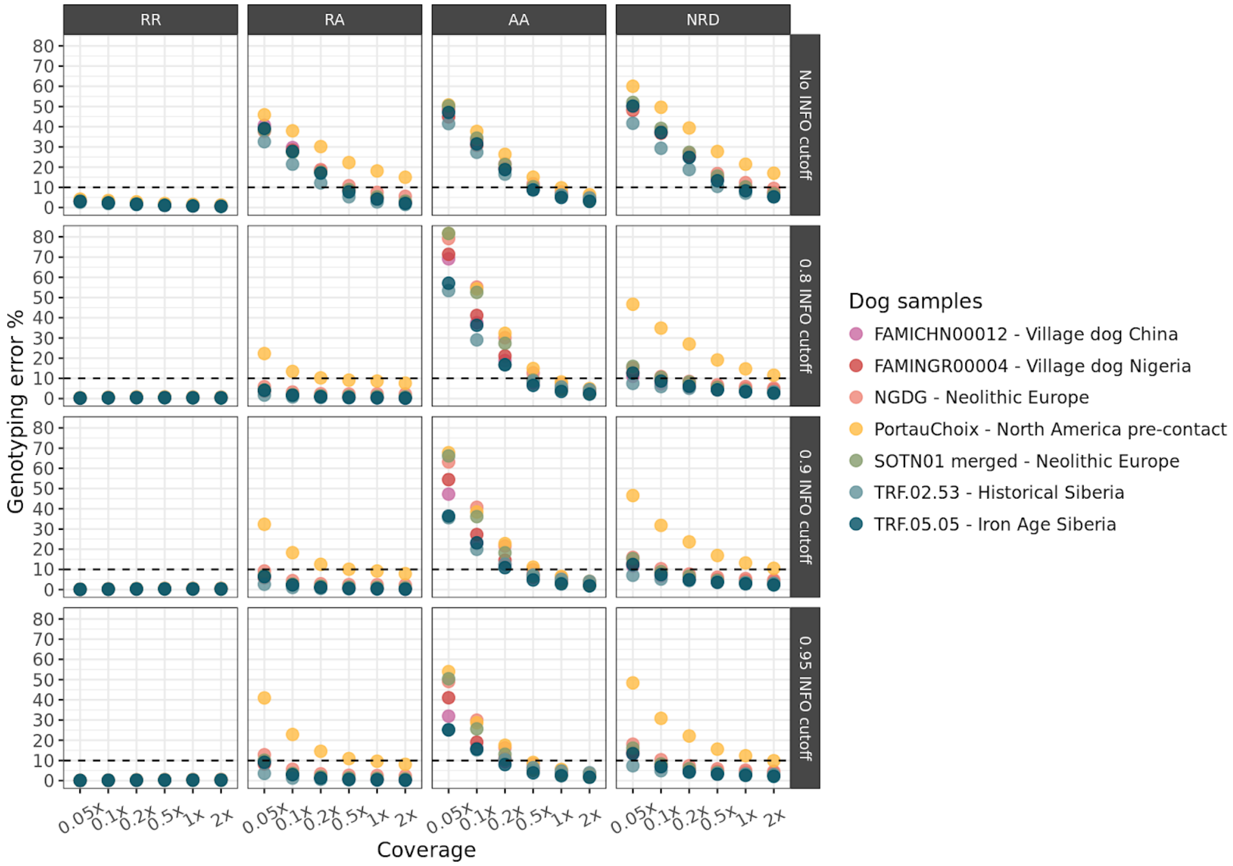

Fig. S23: *Genotyping error between imputed downsampled and the high coverage target dog samples imputed using a dog reference panel for homozygous alternative (AA), heterozygous (RA) and homozygous reference alleles (RR), and the non-reference discordance (NRD) metric estimated by GLIMPSE concordance for all autosomes. Comparisons are shown for different INFO score cutoffs (no cutoff, 0.8, 0.9 and 0.95).*

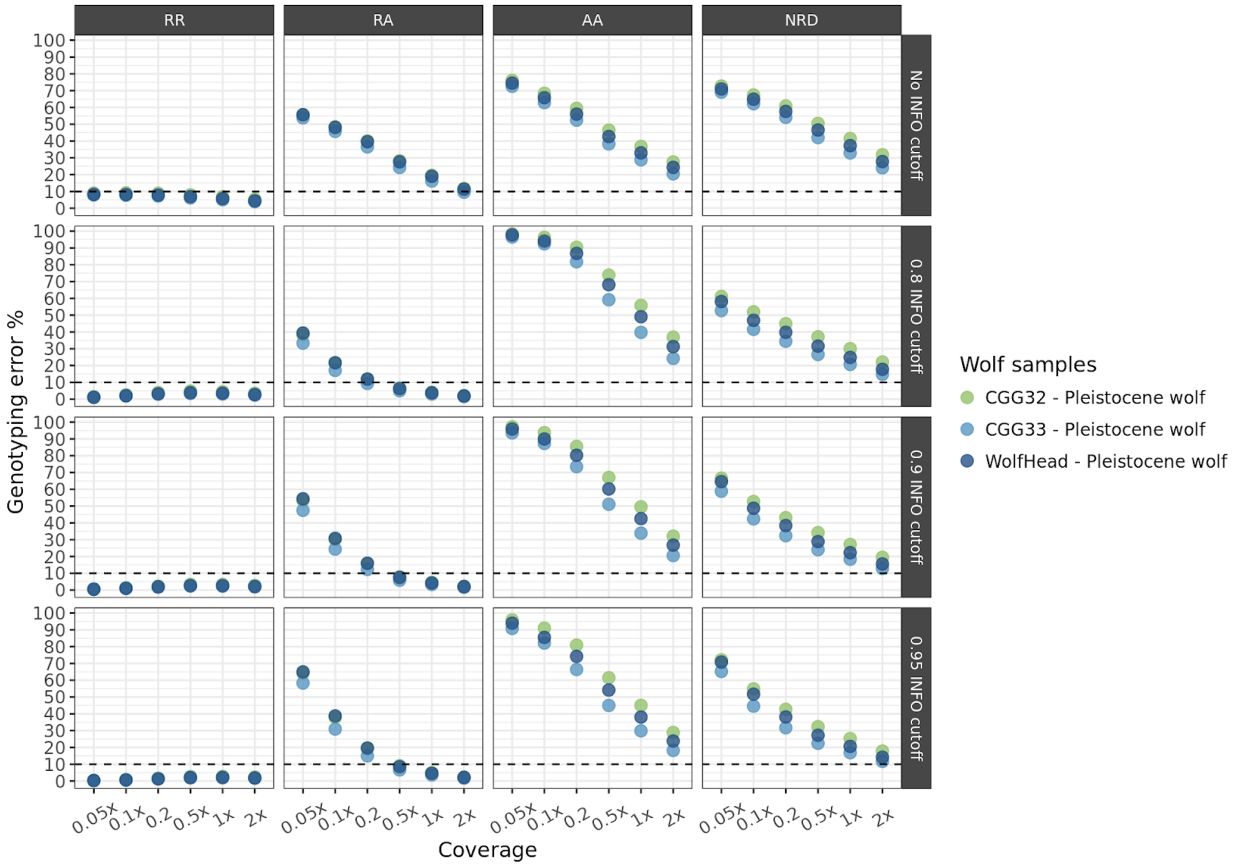

Fig. S24: *Genotyping error between imputed downsampled and the high coverage target Pleistocene wolf samples using a dog reference panel for homozygous alternative (AA), heterozygous (RA) and homozygous reference alleles (RR), and the non-reference discordance (NRD) metric estimated by GLIMPSE concordance for all autosomes. Comparisons are shown for different INFO score cutoffs (no cutoff, 0.8, 0.9 and 0.95).*

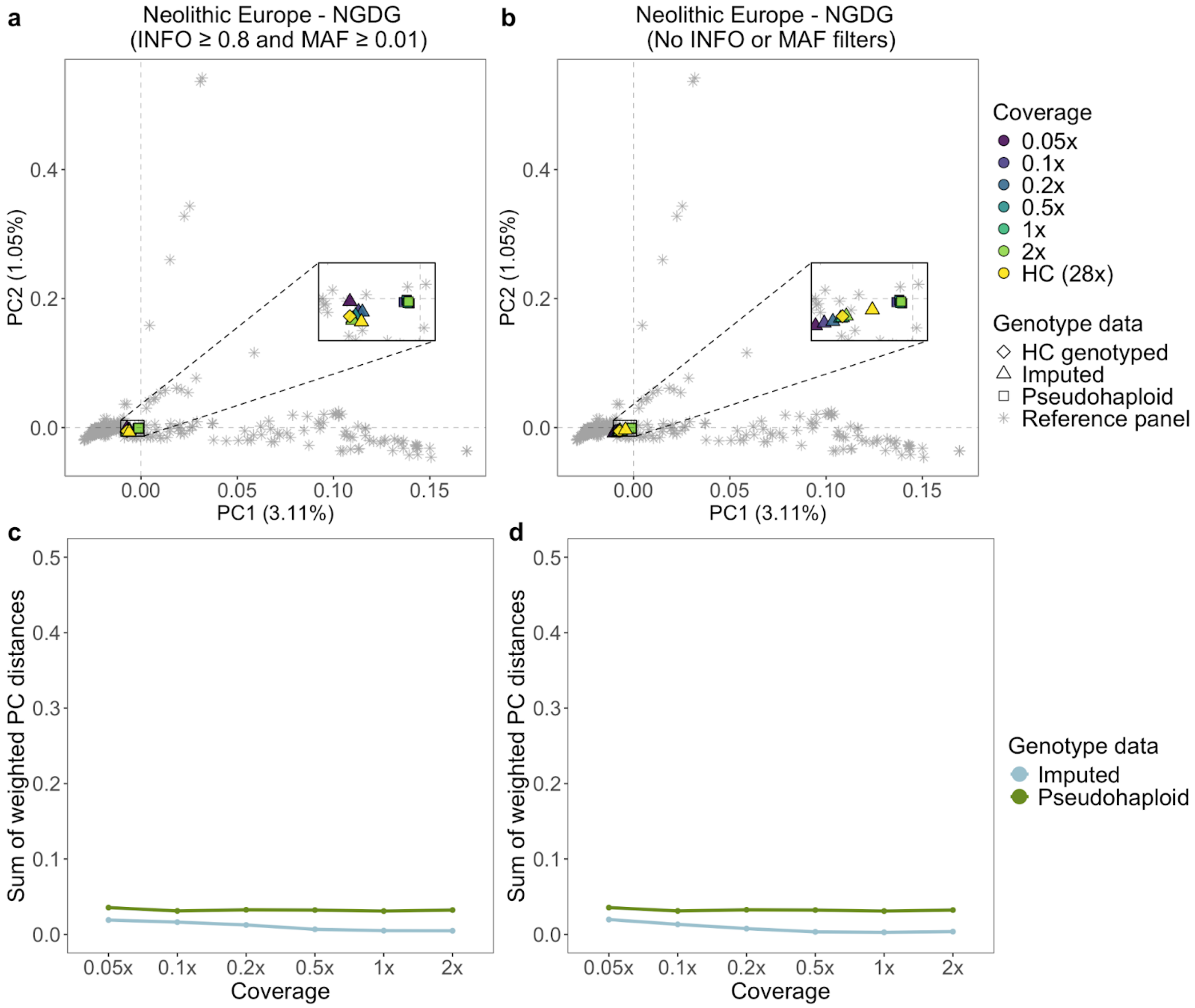

Fig. S25: *a, b) Principal component analysis showcasing the imputation accuracy for the Newgrange Neolithic European dog against its corresponding downsampled pseudohaploid counterpart. The PCs were created using modern dog samples from the reference panel, and then the imputed, pseudohaploid and high coverage genotyped replicas were projected onto them. c, d) Sum of weighted PC distances across all 10 PCs of the imputed and pseudohaploid downsampled individual from its high coverage genotyped version. The left plots (a,c) show the PCA results when applying INFO score and MAF cutoffs on the imputed samples, whereas the right ones (b,d) show when no post-imputation filter is applied. HC: High coverage.*

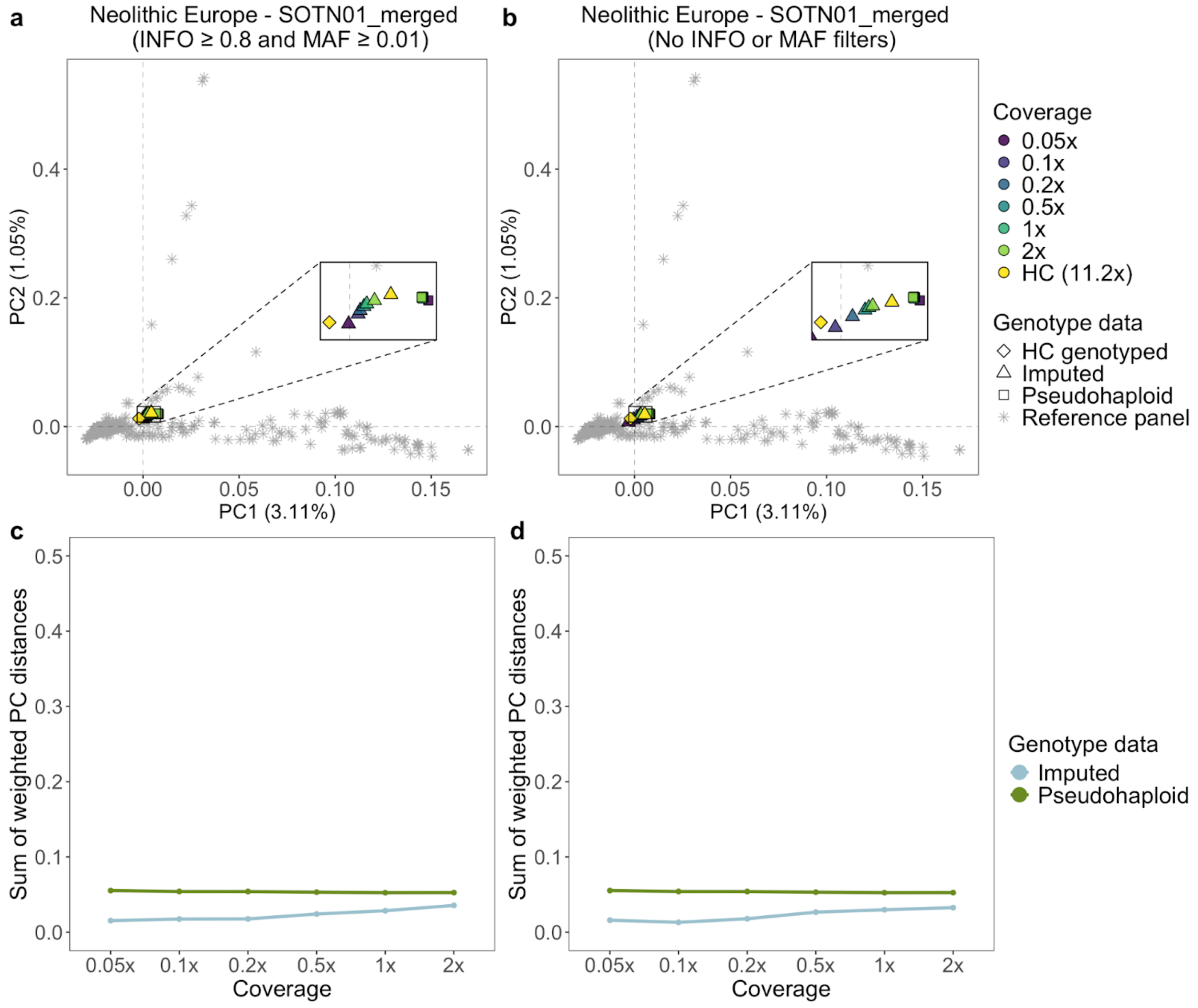

Fig. S26: *a, b) Principal component analysis showcasing the imputation accuracy for the SOTN01 Neolithic European dog against its corresponding downsampled pseudohaploid counterpart. The PCs were created using modern dog samples from the reference panel, and then the imputed, pseudohaploid and high coverage genotyped replicas were projected onto them. c, d) Sum of weighted PC distances across all 10 PCs of the imputed and pseudohaploid downsampled individual from its high coverage genotyped version. The left plots (a,c) show the PCA results when applying INFO score and MAF cutoffs on the imputed samples, whereas the right ones (b,d) show when no post-imputation filter is applied. HC: High coverage.*

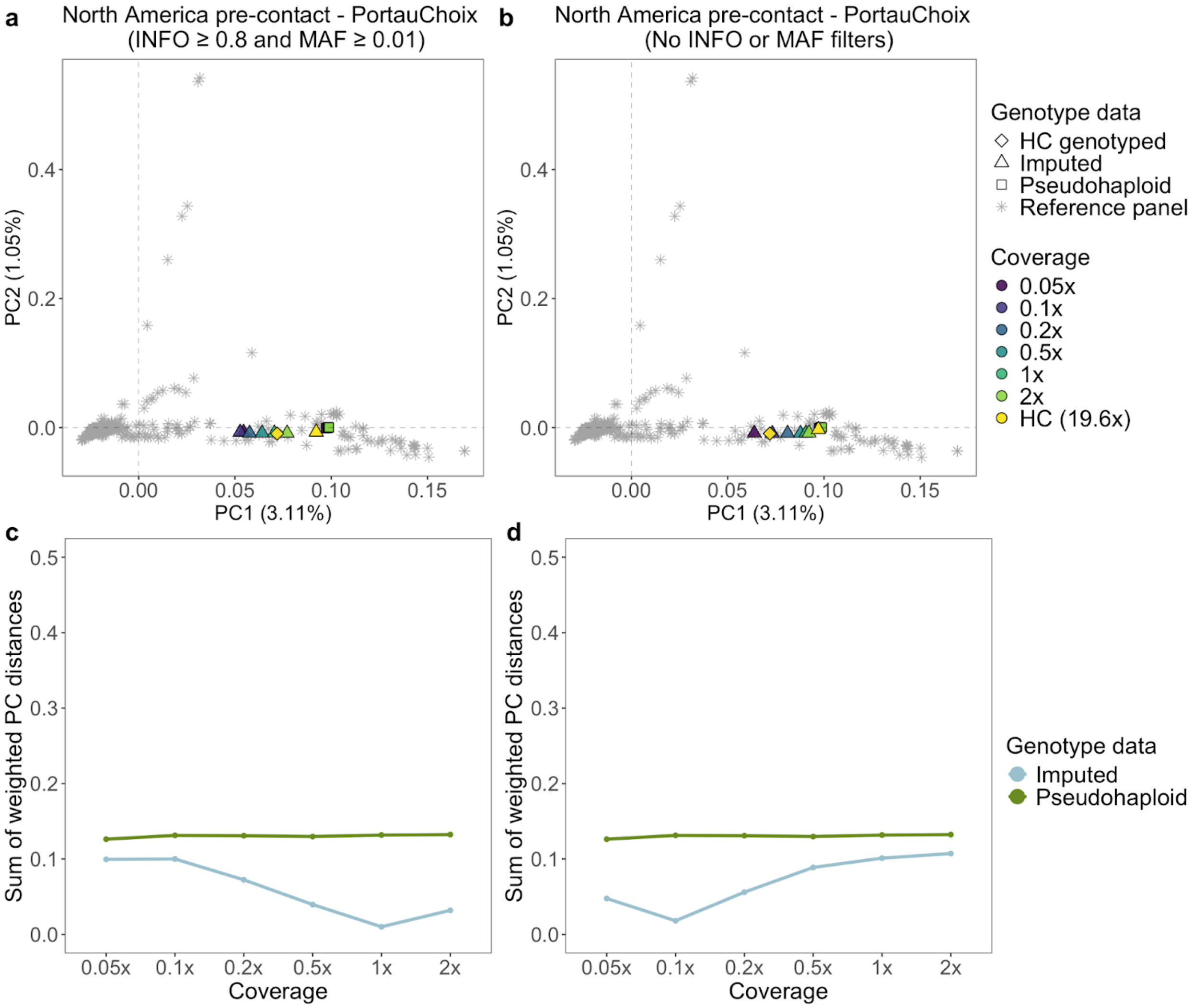

Fig. S27: *a, b) Principal component analysis showcasing the imputation accuracy for the Port au Choix North American pre-contact dog against its corresponding downsampled pseudohaploid counterpart. The PCs were created using modern dog samples from the reference panel, and then the imputed, pseudohaploid and high coverage genotyped replicas were projected onto them. c, d) Sum of weighted PC distances across all 10 PCs of the imputed and pseudohaploid downsampled individual from its high coverage genotyped version. The left plots (a,c) show the PCA results when applying INFO score and MAF cutoffs on the imputed samples, whereas the right ones (b,d) show when no post-imputation filter is applied. HC: High coverage.*

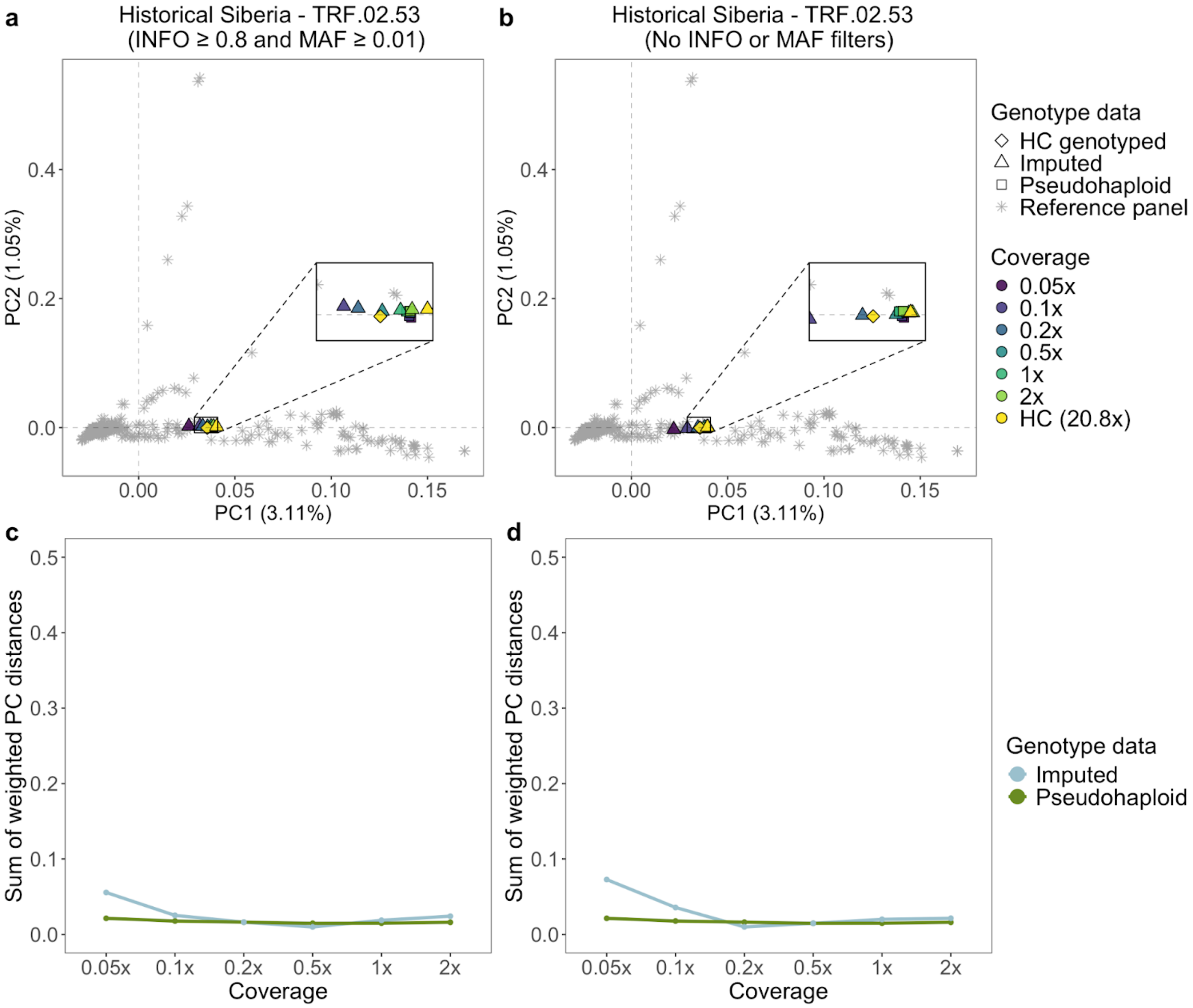

Fig. S28: *a, b) Principal component analysis showcasing the imputation accuracy for the TRF.02.53 historical Siberian dog against its corresponding downsampled pseudohaploid counterpart. The PCs were created using modern dog samples from the reference panel, and then the imputed, pseudohaploid and high coverage genotyped replicas were projected onto them. c, d) Sum of weighted PC distances across all 10 PCs of the imputed and pseudohaploid downsampled individual from its high coverage genotyped version. The left plots (a,c) show the PCA results when applying INFO score and MAF cutoffs on the imputed samples, whereas the right ones (b,d) show when no post-imputation filter is applied. HC: High coverage.*

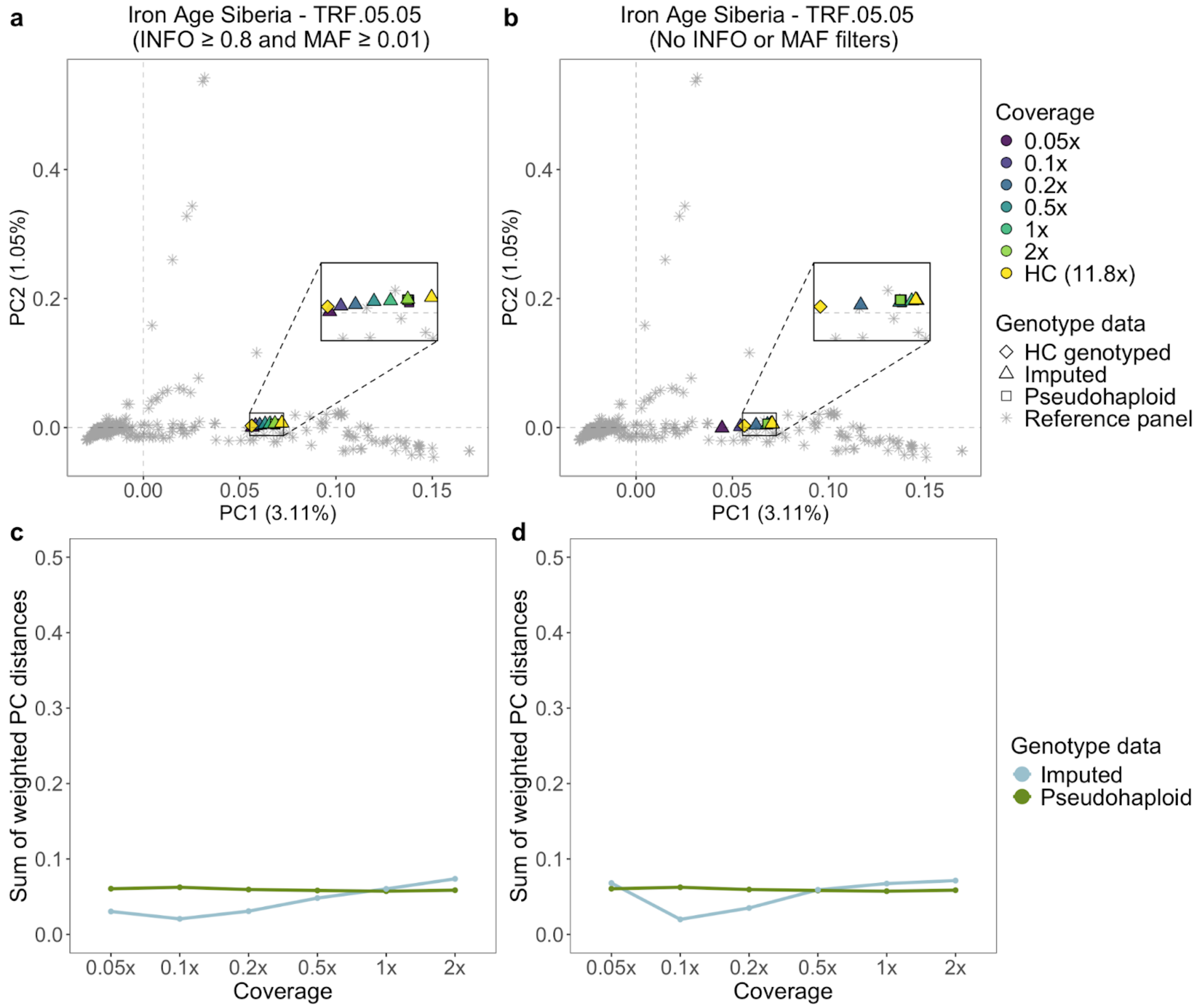

Fig. S29: *a, b) Principal component analysis showcasing the imputation accuracy for the TRF.05.05 Iron Age Siberian dog against its corresponding downsampled pseudohaploid counterpart. The PCs were created using modern dog samples from the reference panel, and then the imputed, pseudohaploid and high coverage genotyped replicas were projected onto them. c, d) Sum of weighted PC distances across all 10 PCs of the imputed and pseudohaploid downsampled individual from its high coverage genotyped version. The left plots (a,c) show the PCA results when applying INFO score and MAF cutoffs on the imputed samples, whereas the right ones (b,d) show when no post-imputation filter is applied. HC: High coverage.*

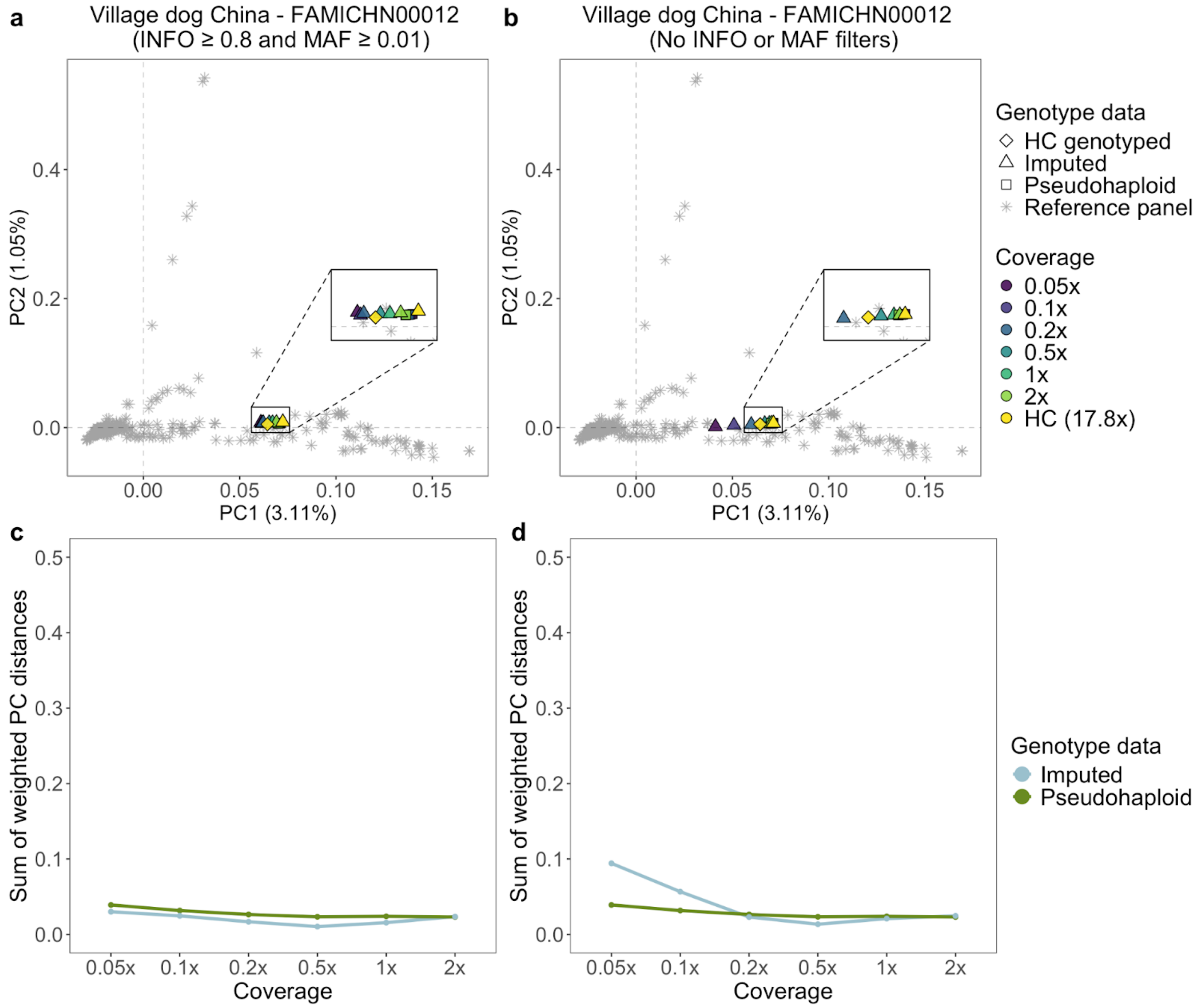

Fig. S30: *a, b) Principal component analysis showcasing the imputation accuracy for the FAMICHN00012 Chinese village dog against its corresponding downsampled pseudohaploid counterpart. The PCs were created using modern dog samples from the reference panel, and then the imputed, pseudohaploid and high coverage genotyped replicas were projected onto them. c, d) Sum of weighted PC distances across all 10 PCs of the imputed and pseudohaploid downsampled individual from its high coverage genotyped version. The left plots (a,c) show the PCA results when applying INFO score and MAF cutoffs on the imputed samples, whereas the right ones (b,d) show when no post-imputation filter is applied. HC: High coverage.*

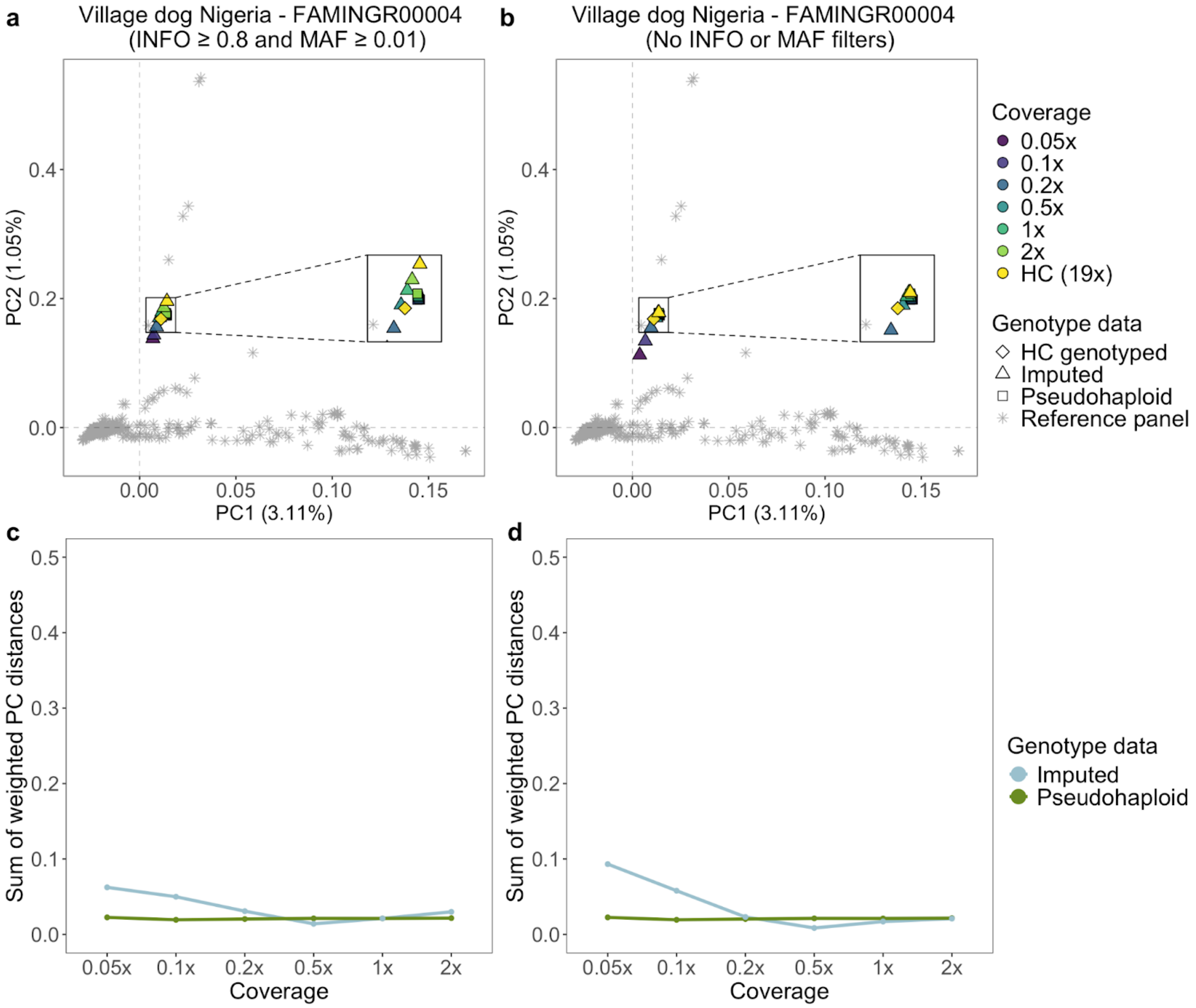

Fig. S31: *a, b) Principal component analysis showcasing the imputation accuracy for the FAMINGR00004 Nigerian village dog against its corresponding downsampled pseudohaploid counterpart. The PCs were created using modern dog samples from the reference panel, and then the imputed, pseudohaploid and high coverage genotyped replicas were projected onto them. c, d) Sum of weighted PC distances across all 10 PCs of the imputed and pseudohaploid downsampled individual from its high coverage genotyped version. The left plots (a,c) show the PCA results when applying INFO score and MAF cutoffs on the imputed samples, whereas the right ones (b,d) show when no post-imputation filter is applied. HC: High coverage.*

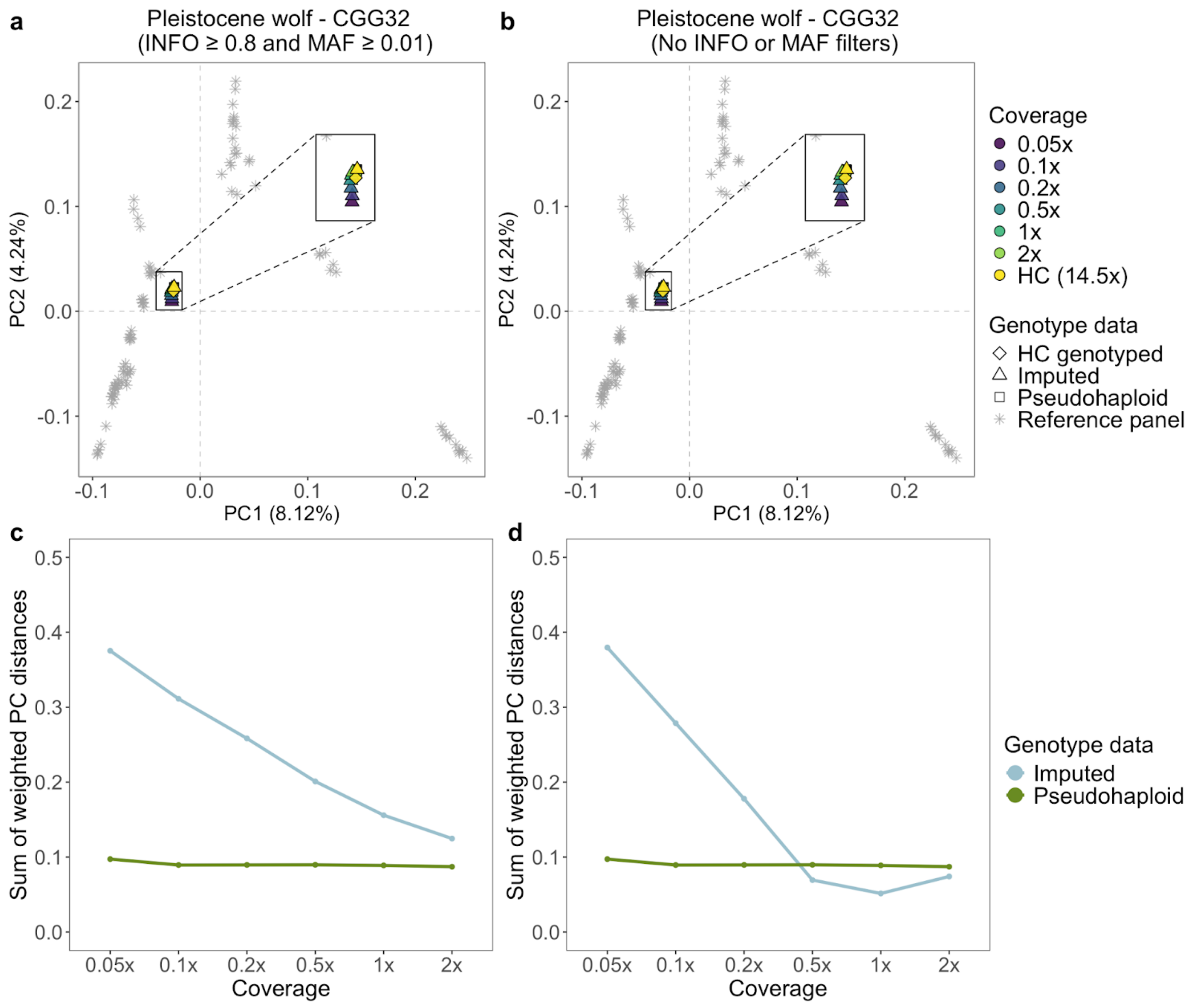

Fig. S32: *a, b) Principal component analysis showcasing the imputation accuracy for the CGG32 Pleistocene wolf against its corresponding downsampled pseudohaploid counterpart. The PCs were created using modern wolf samples from the reference panel, and then the imputed, pseudohaploid and high coverage genotyped replicas were projected onto them. c, d) Sum of weighted PC distances across all 10 PCs of the imputed and pseudohaploid downsampled individual from its high coverage genotyped version. The left plots (a,c) show the PCA results when applying INFO score and MAF cutoffs on the imputed samples, whereas the right ones (b,d) show when no post-imputation filter is applied. HC: High coverage.*

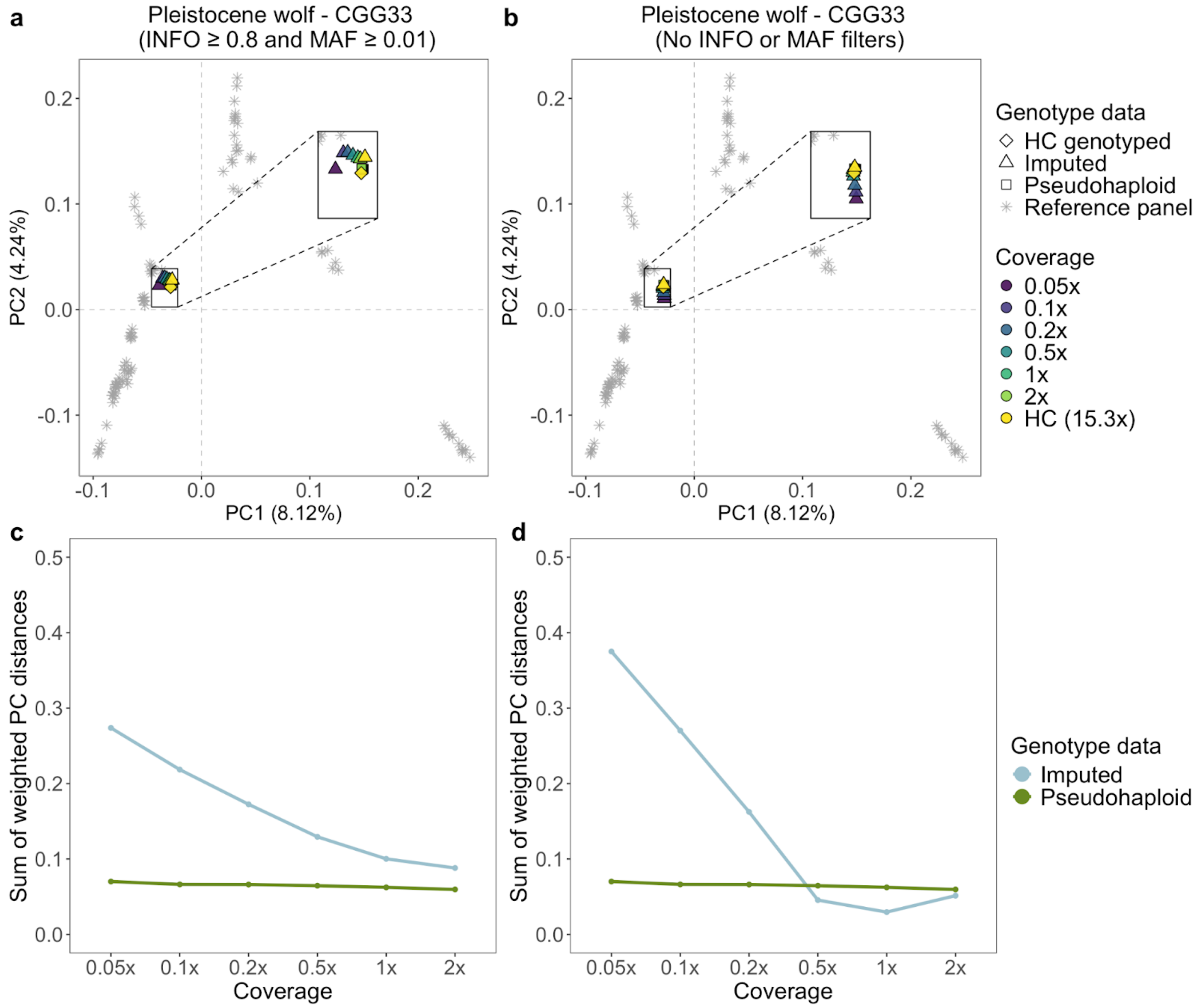

Fig. S33: *a, b) Principal component analysis showcasing the imputation accuracy for the CGG33 Pleistocene wolf against its corresponding downsampled pseudohaploid counterpart. The PCs were created using modern wolf samples from the reference panel, and then the imputed, pseudohaploid and high coverage genotyped replicas were projected onto them. c, d) Sum of weighted PC distances across all 10 PCs of the imputed and pseudohaploid downsampled individual from its high coverage genotyped version. The left plots (a,c) show the PCA results when applying INFO score and MAF cutoffs on the imputed samples, whereas the right ones (b,d) show when no post-imputation filter is applied. HC: High coverage.*

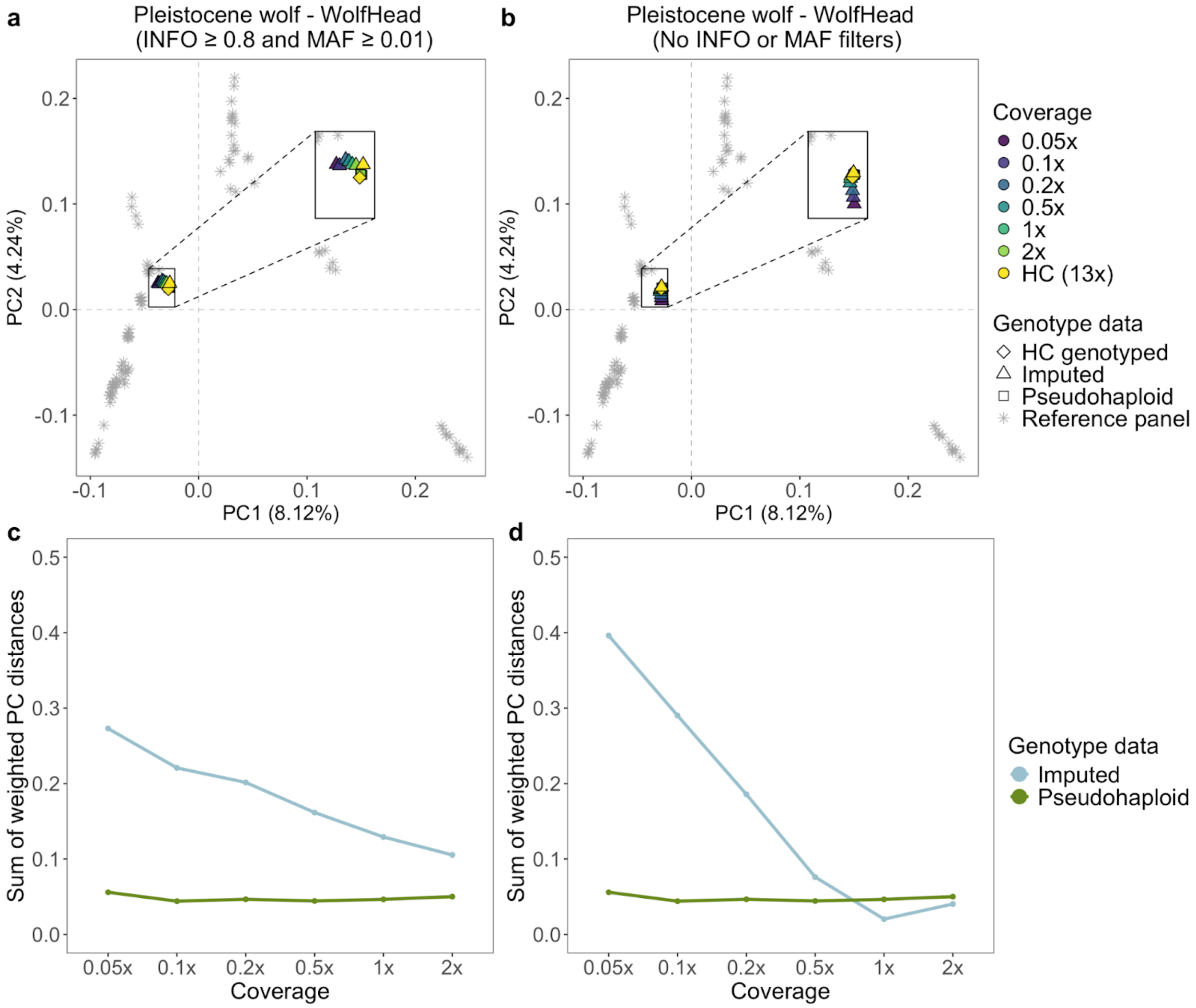

Fig. S34: *a, b) Principal component analysis showcasing the imputation accuracy for the WolfHead Pleistocene wolf against its corresponding downsampled pseudohaploid counterpart. The PCs were created using modern wolf samples from the reference panel, and then the imputed, pseudohaploid and high coverage genotyped replicas were projected onto them. c, d) Sum of weighted PC distances across all 10 PCs of the imputed and pseudohaploid downsampled individual from its high coverage genotyped version. The left plots (a,c) show the PCA results when applying INFO score and MAF cutoffs on the imputed samples, whereas the right ones (b,d) show when no post-imputation filter is applied. HC: High coverage.*

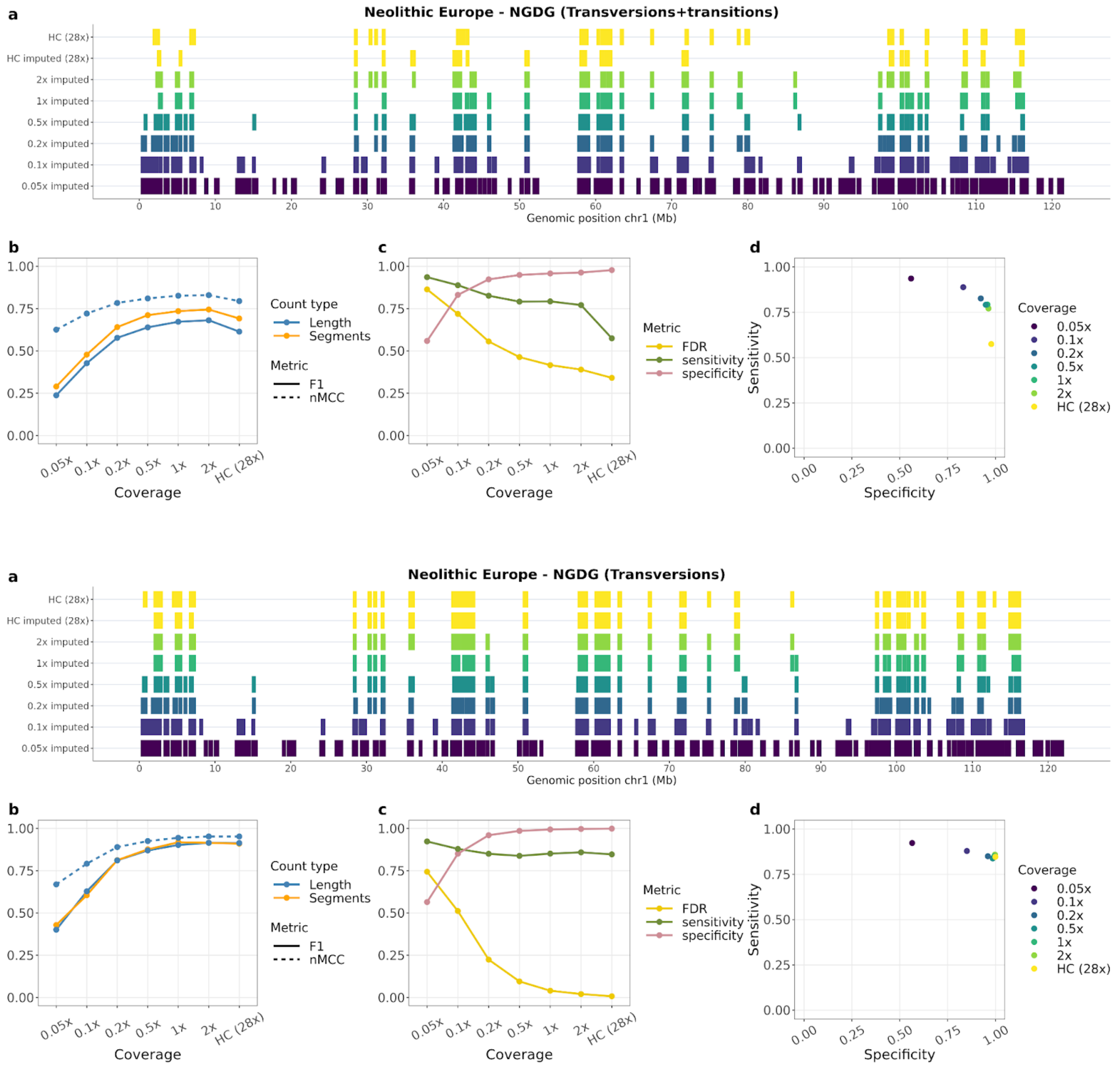

Fig. S35: *Overlap of ROH called from the Newgrange Neolithic European dog for each imputed downsampled replicate using ROH estimates from the ground truth, using transversions and transitions (top panel) or only transversions (bottom panel). a) ROH called across the six tested coverages and the high coverage imputed and genotyped (ground truth) sample on chromosome one. b) Accuracy of recovering ROH across all tested coverages based on total length in bp (blue lines) and total number of segments (orange line) using the F1-score (solid line) and normalised Matthew correlation coefficient (nMCC) (dotted line). c) FDR, sensitivity and specificity measurements based on the total length of recovered ROH per coverage. d) Sensitivity plotted against specificity estimated based on the total length of recovered ROH across all tested coverages. HC: High coverage.*

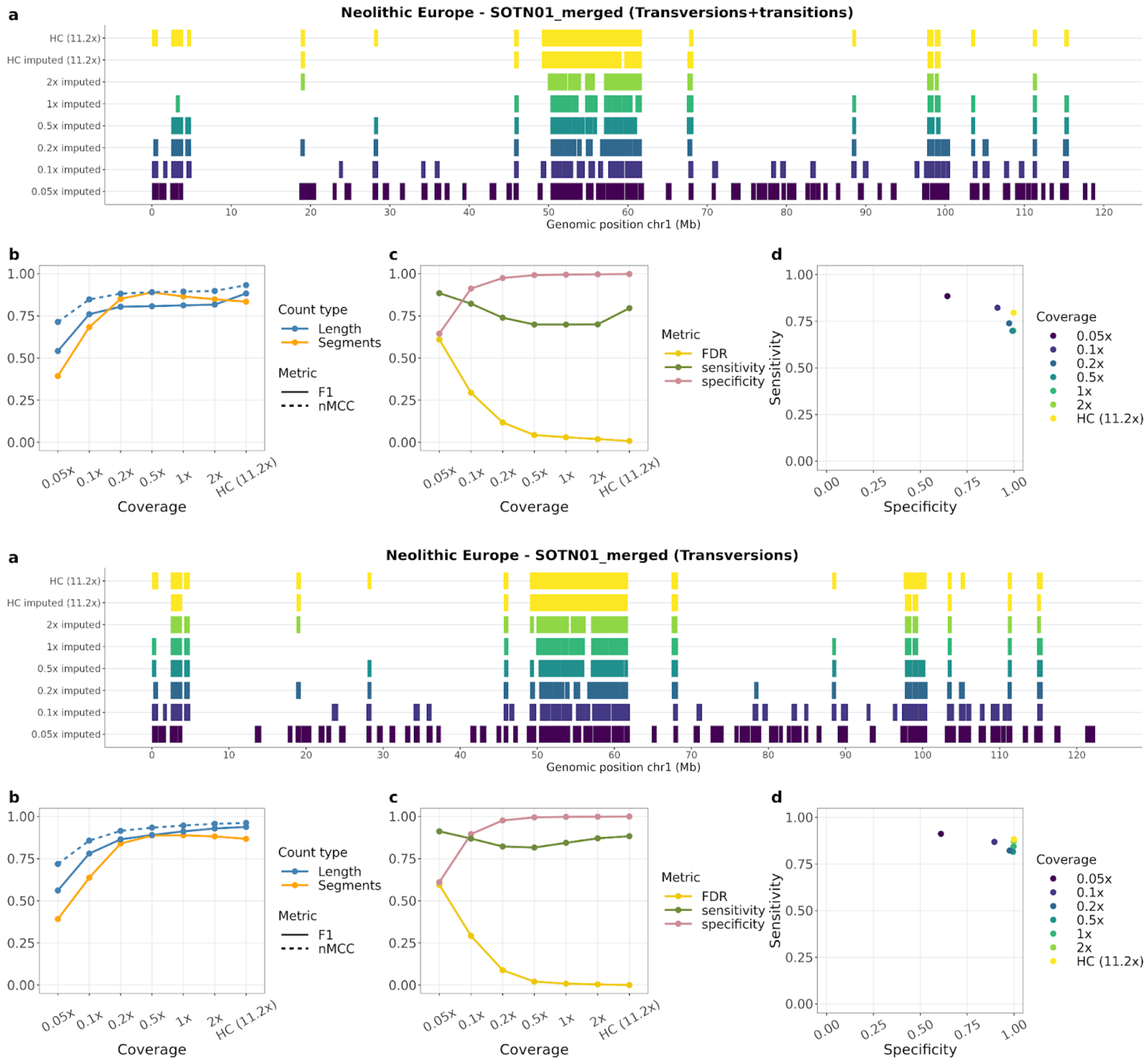

Fig. S36: *Overlap of ROH called from the SOTN01 Neolithic European dog for each imputed downsampled replicate using ROH estimates from the ground truth, using transversions and transitions (top panel) or only transversions (bottom panel). a) ROH called across the six tested coverages and the high coverage imputed and genotyped (ground truth) sample on chromosome one. b) Accuracy of recovering ROH across all tested coverages based on total length in bp (blue lines) and total number of segments (orange line) using the F1-score (solid line) and normalised Matthew correlation coefficient (nMCC) (dotted line). c) FDR, sensitivity and specificity measurements based on the total length of recovered ROH per coverage. d) Sensitivity plotted against specificity estimated based on the total length of recovered ROH across all tested coverages. HC: High coverage.*

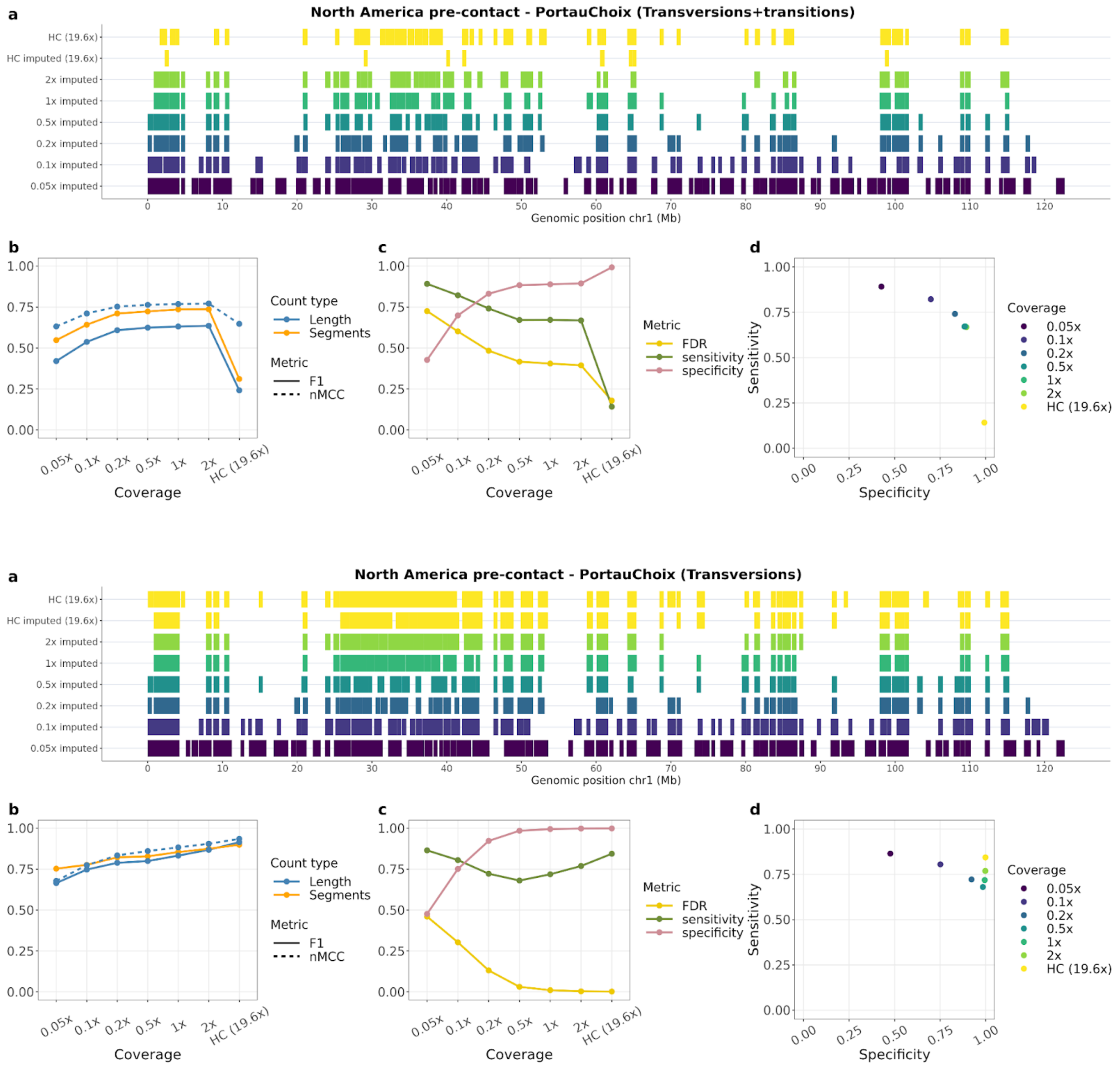

Fig. S37: *Overlap of ROH called from the Port au Choix North American pre-contact dog for each imputed downsampled replicate using ROH estimates from the ground truth, using transversions and transitions (top panel) or only transversions (bottom panel). a) ROH called across the six tested coverages and the high coverage imputed and genotyped (ground truth) sample on chromosome one. b) Accuracy of recovering ROH across all tested coverages based on total length in bp (blue lines) and total number of segments (orange line) using the F1-score (solid line) and normalised Matthew correlation coefficient (nMCC) (dotted line). c) FDR, sensitivity and specificity measurements based on the total length of recovered ROH per coverage. d) Sensitivity plotted against specificity estimated based on the total length of recovered ROH across all tested coverages. HC: High coverage.*

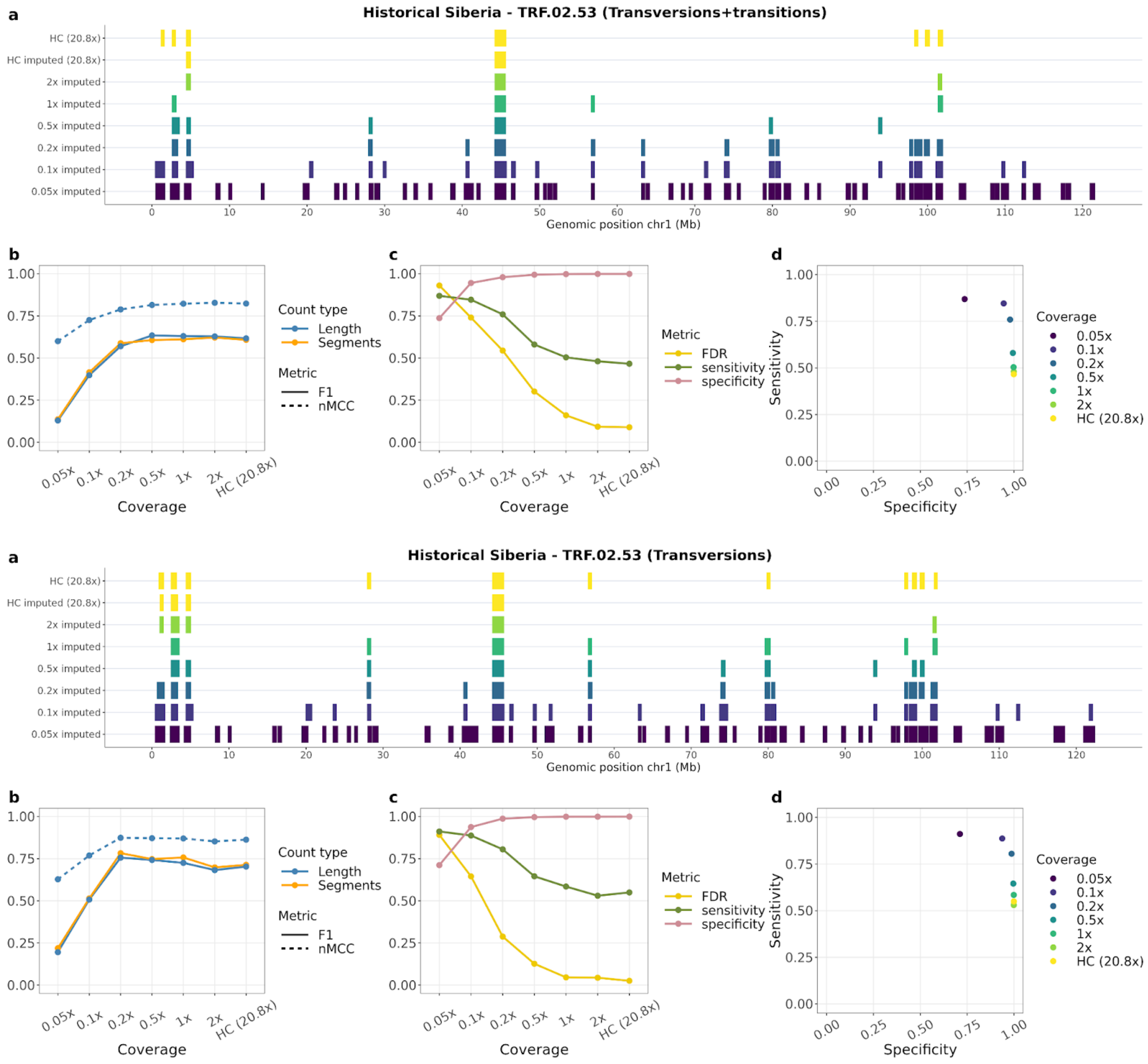

Fig. S38: *Overlap of ROH called from the TRF.02.53 historical Siberian dog for each imputed downsampled replicate using ROH estimates from the ground truth, using transversions and transitions (top panel) or only transversions (bottom panel). a) ROH called across the six tested coverages and the high coverage imputed and genotyped (ground truth) sample on chromosome one. b) Accuracy of recovering ROH across all tested coverages based on total length in bp (blue lines) and total number of segments (orange line) using the F1-score (solid line) and normalised Matthew correlation coefficient (nMCC) (dotted line). c) FDR, sensitivity and specificity measurements based on the total length of recovered ROH per coverage. d) Sensitivity plotted against specificity estimated based on the total length of recovered ROH across all tested coverages. HC: High coverage.*

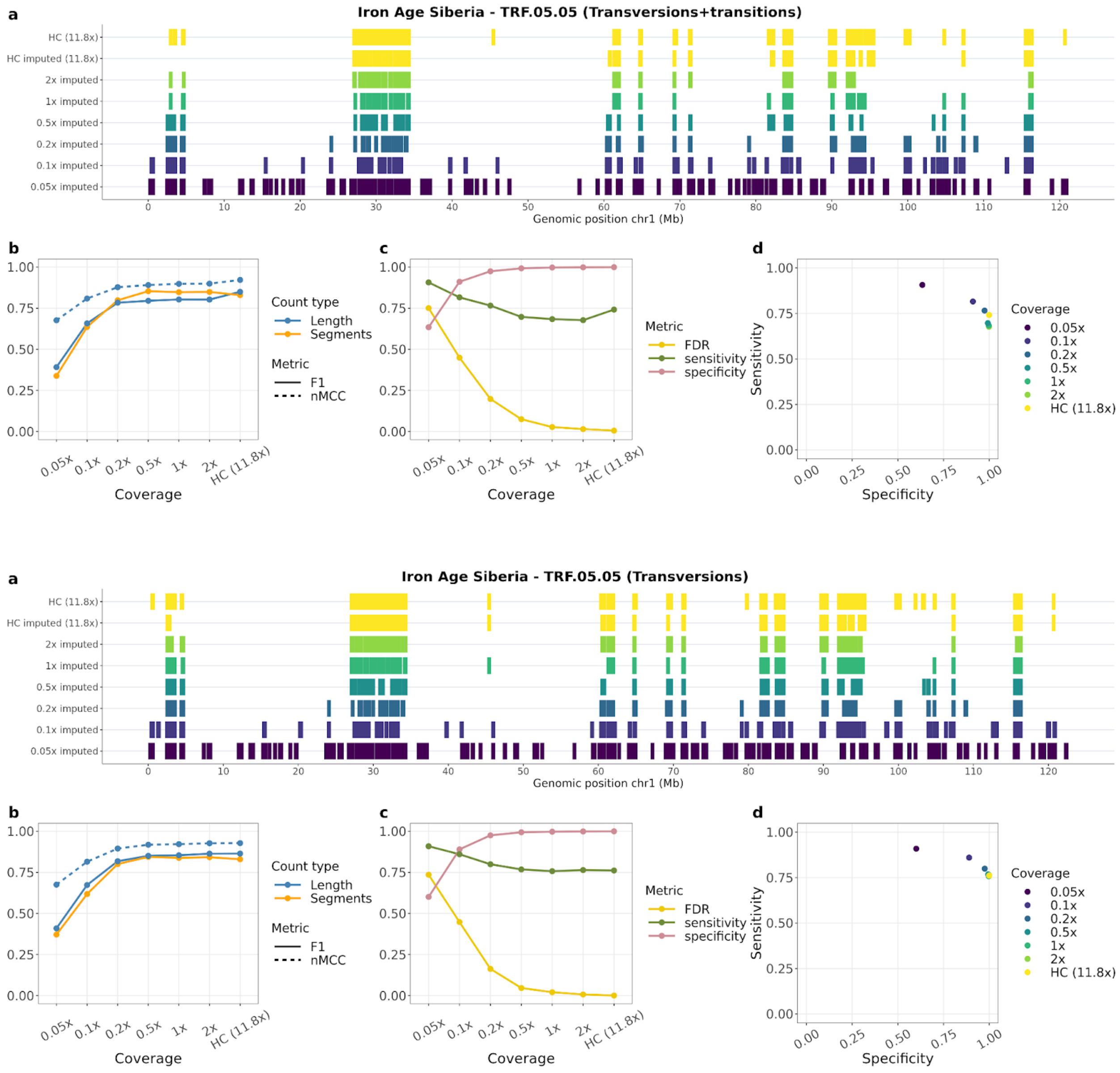

Fig. S39: *Overlap of ROH called from the TRF.05.05 Iron Age Siberian dog for each imputed downsampled replicate using ROH estimates from the ground truth, using transversions and transitions (top panel) or only transversions (bottom panel). a) ROH called across the six tested coverages and the high coverage imputed and genotyped (ground truth) sample on chromosome one. b) Accuracy of recovering ROH across all tested coverages based on total length in bp (blue lines) and total number of segments (orange line) using the F1-score (solid line) and normalised Matthew correlation coefficient (nMCC) (dotted line). c) FDR, sensitivity and specificity measurements based on the total length of recovered ROH per coverage. d) Sensitivity plotted against specificity estimated based on the total length of recovered ROH across all tested coverages. HC: High coverage.*

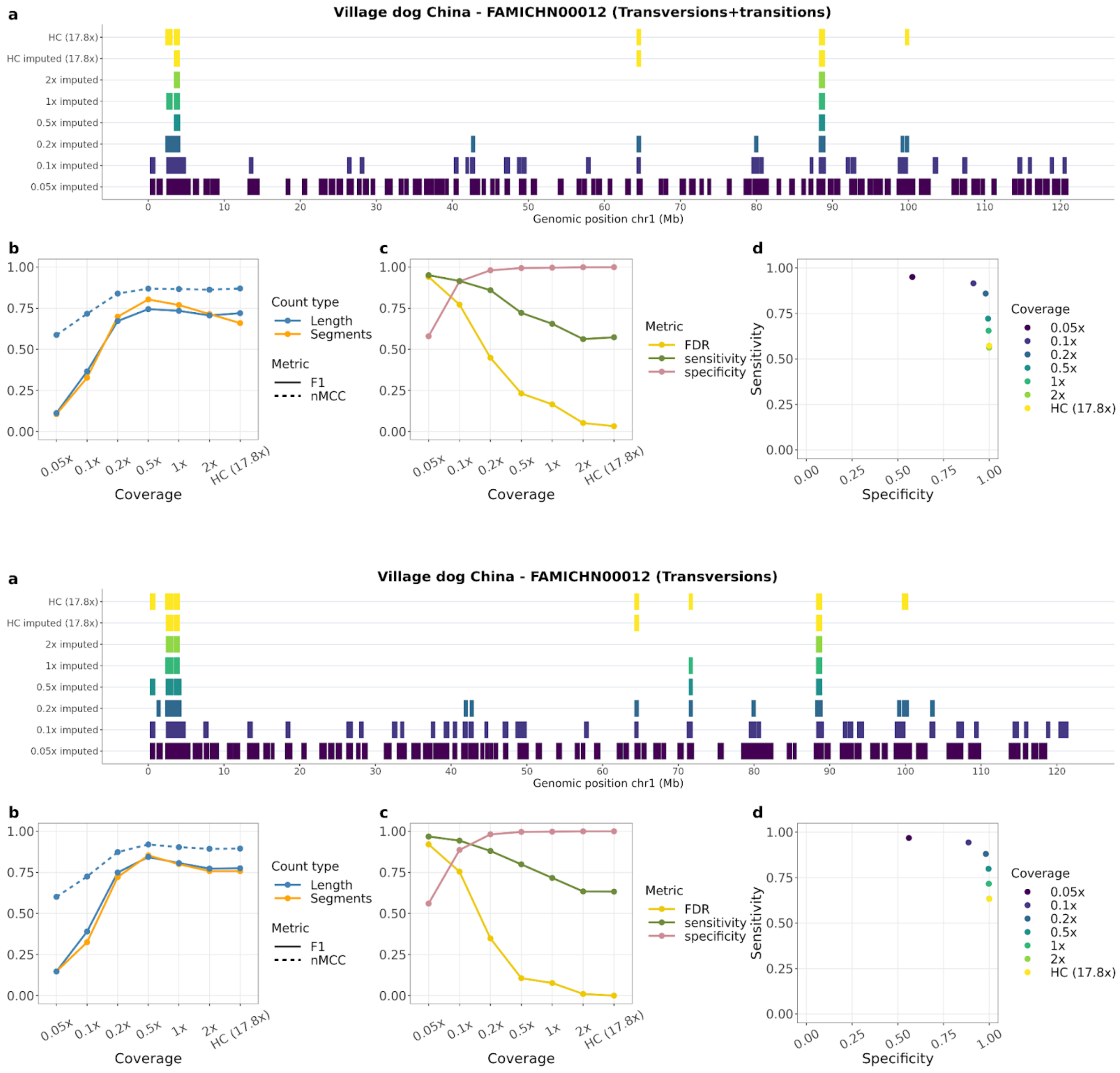

Fig. S40: *Overlap of ROH called from the FAMICHN00012 Chinese Village dog for each imputed downsampled replicate using ROH estimates from the ground truth, using transversions and transitions (top panel) or only transversions (bottom panel). a) ROH called across the six tested coverages and the high coverage imputed and genotyped (ground truth) sample on chromosome one. b) Accuracy of recovering ROH across all tested coverages based on total length in bp (blue lines) and total number of segments (orange line) using the F1-score (solid line) and normalised Matthew correlation coefficient (nMCC) (dotted line). c) FDR, sensitivity and specificity measurements based on the total length of recovered ROH per coverage. d) Sensitivity plotted against specificity estimated based on the total length of recovered ROH across all tested coverages. HC: High coverage.*

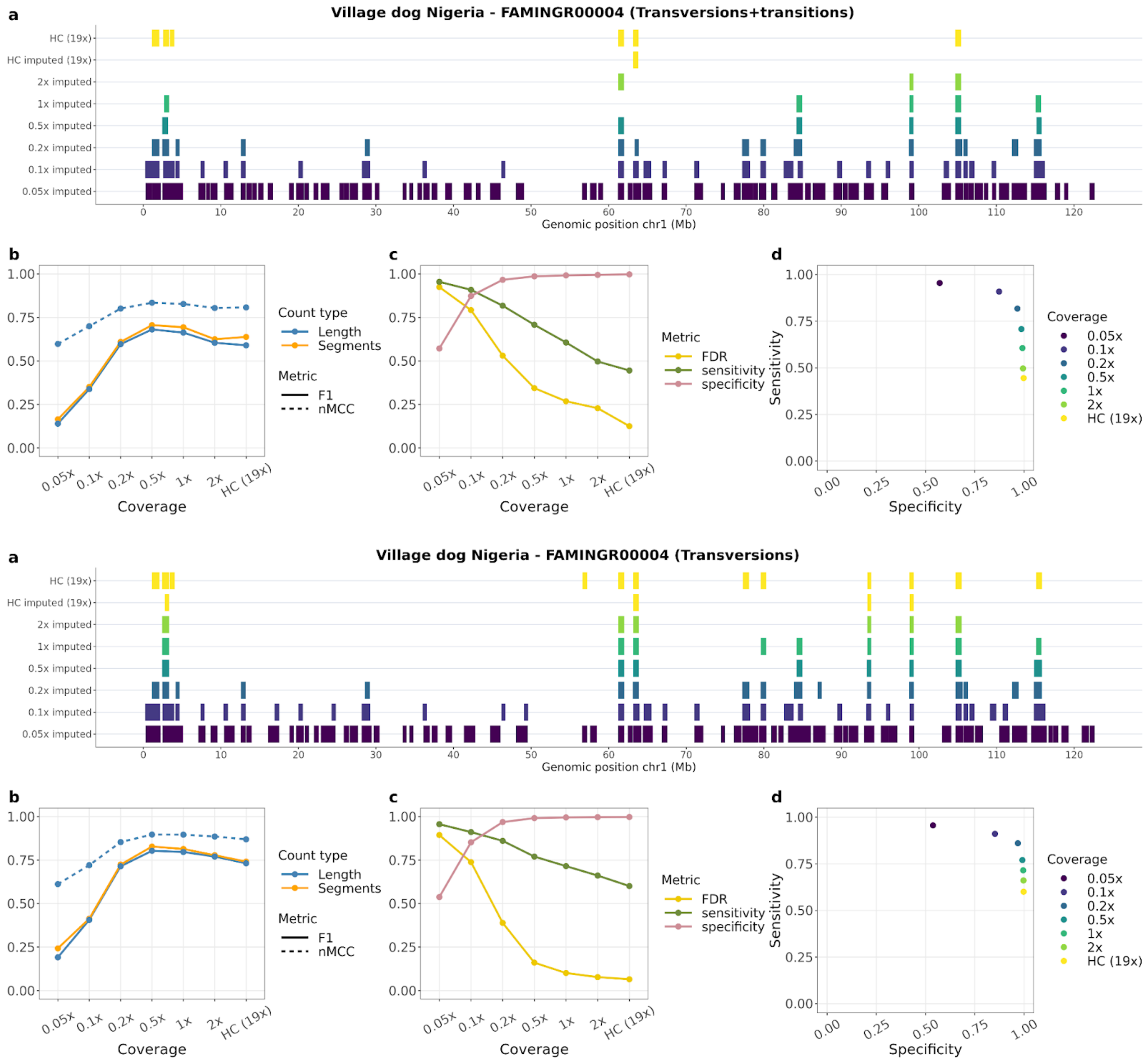

Fig. S41: *Overlap of ROH called from the FAMINGR00004 Nigerian Village dog for each imputed downsampled replicate using ROH estimates from the ground truth, using transversions and transitions (top panel) or only transversions (bottom panel). a) ROH called across the six tested coverages and the high coverage imputed and genotyped (ground truth) sample on chromosome one. b) Accuracy of recovering ROH across all tested coverages based on total length in bp (blue lines) and total number of segments (orange line) using the F1-score (solid line) and normalised Matthew correlation coefficient (nMCC) (dotted line). c) FDR, sensitivity and specificity measurements based on the total length of recovered ROH per coverage. d) Sensitivity plotted against specificity estimated based on the total length of recovered ROH across all tested coverages. HC: High coverage.*

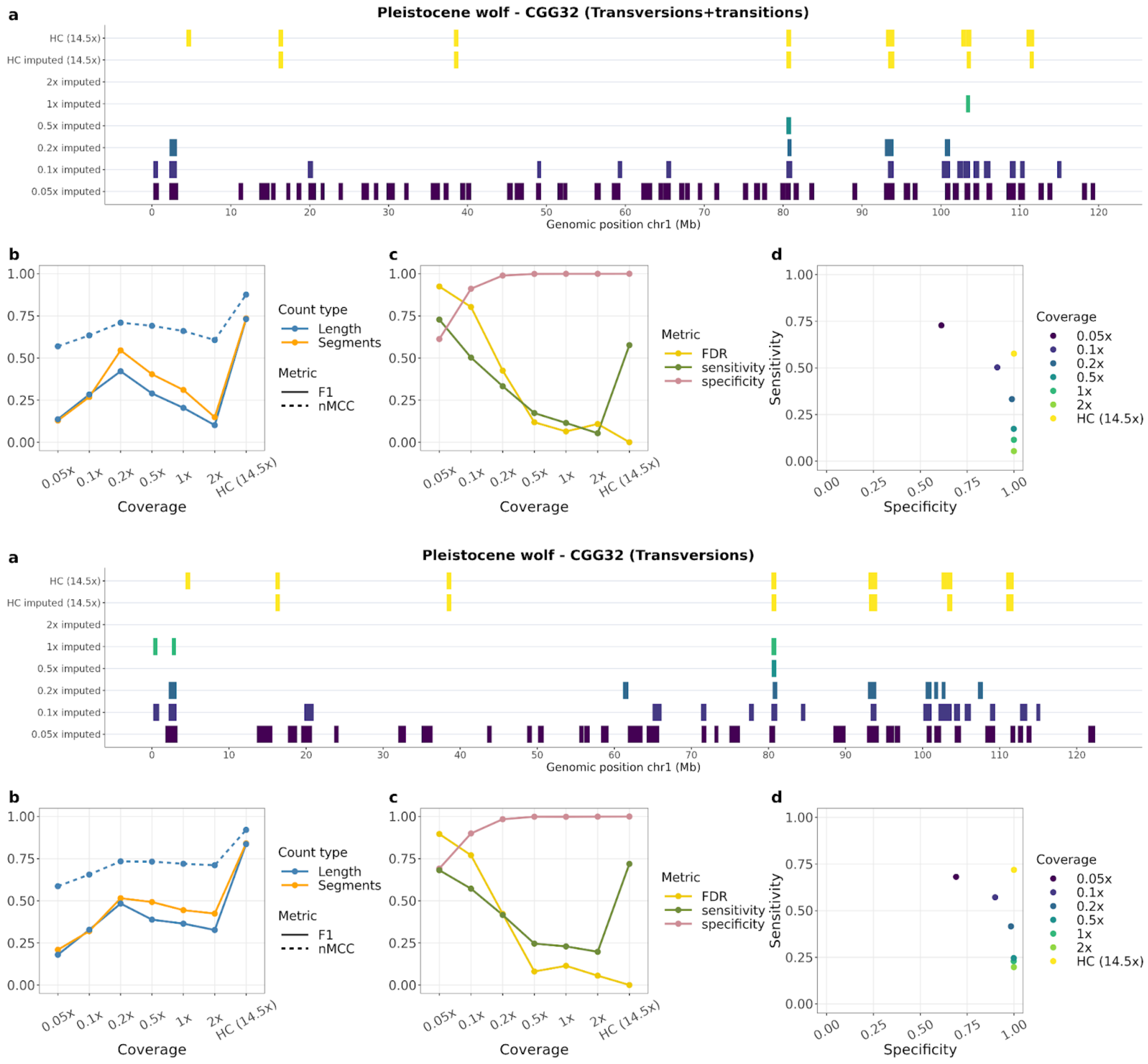

Fig. S42: *Overlap of ROH called from the CGG32 Pleistocene wolf for each imputed downsampled replicate using ROH estimates from the ground truth, using transversions and transitions (top panel) or only transversions (bottom panel). a) ROH called across the six tested coverages and the high coverage imputed and genotyped (ground truth) sample on chromosome one. b) Accuracy of recovering ROH across all tested coverages based on total length in bp (blue lines) and total number of segments (orange line) using the F1-score (solid line) and normalised Matthew correlation coefficient (nMCC) (dotted line). c) FDR, sensitivity and specificity measurements based on the total length of recovered ROH per coverage. d) Sensitivity plotted against specificity estimated based on the total length of recovered ROH across all tested coverages. HC: High coverage.*

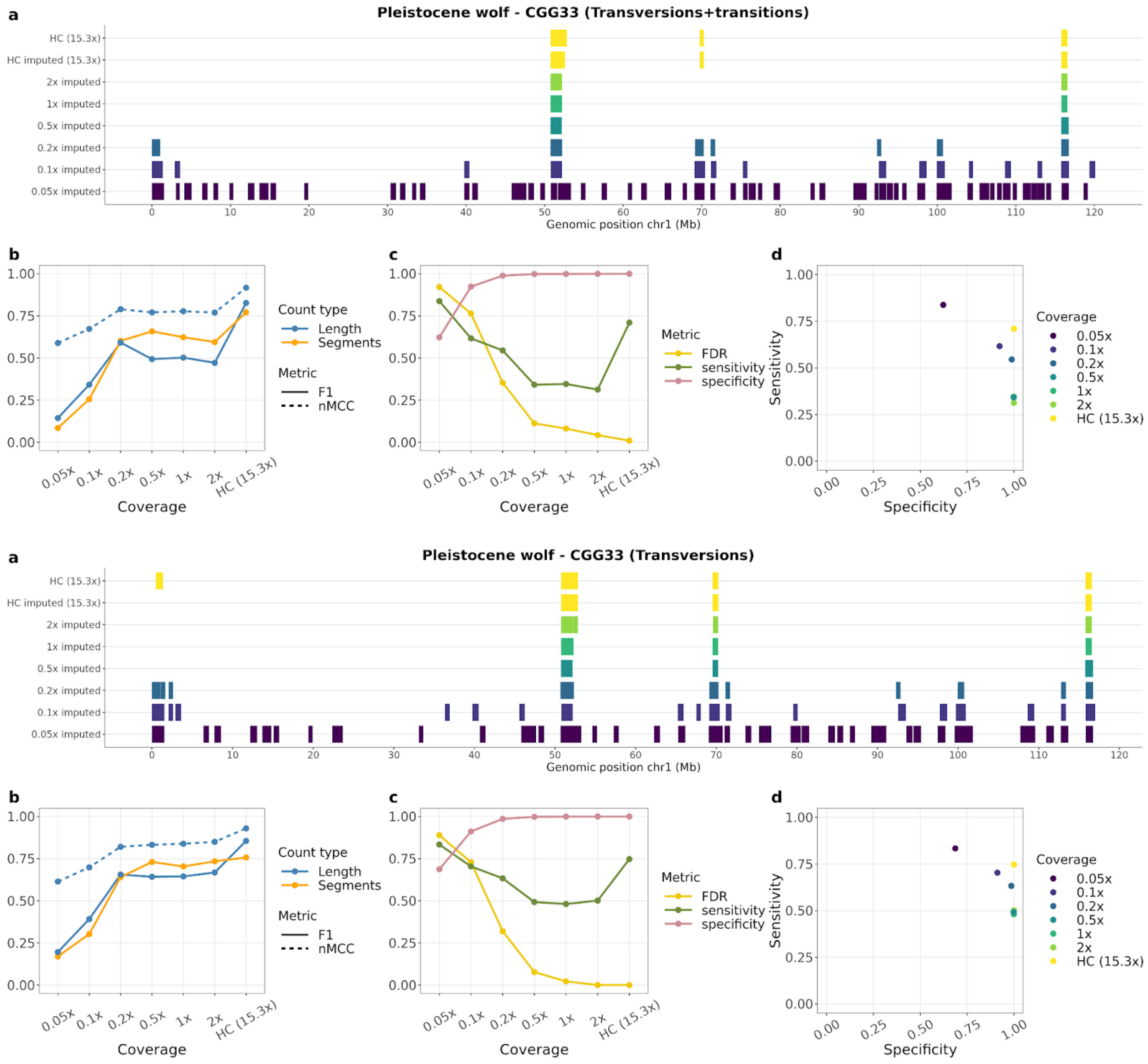

Fig. S43: *Overlap of ROH called from the CGG33 Pleistocene wolf for each imputed downsampled replicate using ROH estimates from the ground truth, using transversions and transitions (top panel) or only transversions (bottom panel). a) ROH called across the six tested coverages and the high coverage imputed and genotyped (ground truth) sample on chromosome one. b) Accuracy of recovering ROH across all tested coverages based on total length in bp (blue lines) and total number of segments (orange line) using the F1-score (solid line) and normalised Matthew correlation coefficient (nMCC) (dotted line). c) FDR, sensitivity and specificity measurements based on the total length of recovered ROH per coverage. d) Sensitivity plotted against specificity estimated based on the total length of recovered ROH across all tested coverages. HC: High coverage.*

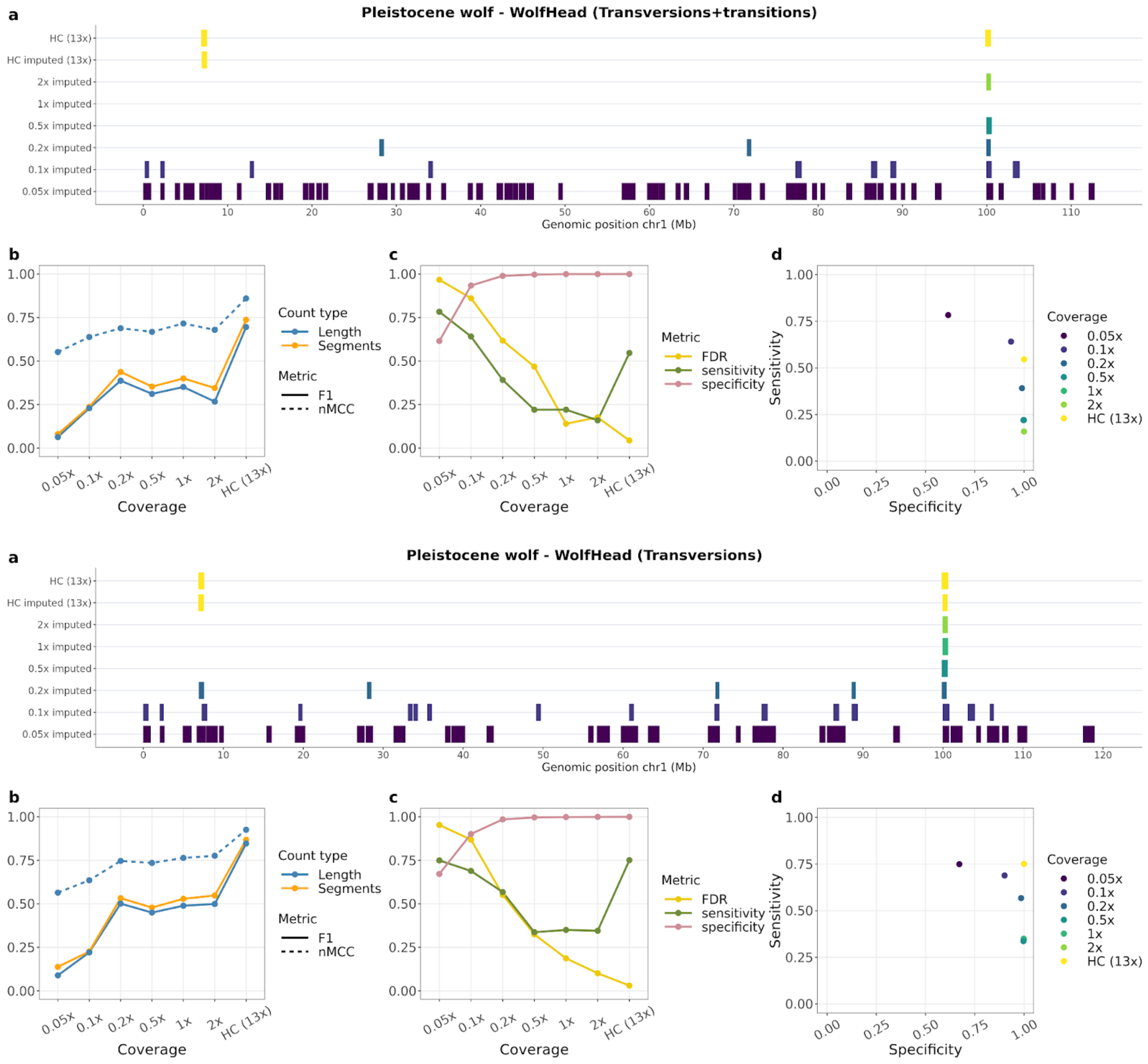

Fig. S44: *Overlap of ROH called from the WolfHead Pleistocene wolf for each imputed downsampled replicate using ROH estimates from the ground truth, using transversions and transitions (top panel) or only transversions (bottom panel). a) ROH called across the six tested coverages and the high coverage imputed and genotyped (ground truth) sample on chromosome one. b) Accuracy of recovering ROH across all tested coverages based on total length in bp (blue lines) and total number of segments (orange line) using the F1-score (solid line) and normalised Matthew correlation coefficient (nMCC) (dotted line). c) FDR, sensitivity and specificity measurements based on the total length of recovered ROH per coverage. d) Sensitivity plotted against specificity estimated based on the total length of recovered ROH across all tested coverages. HC: High coverage.*

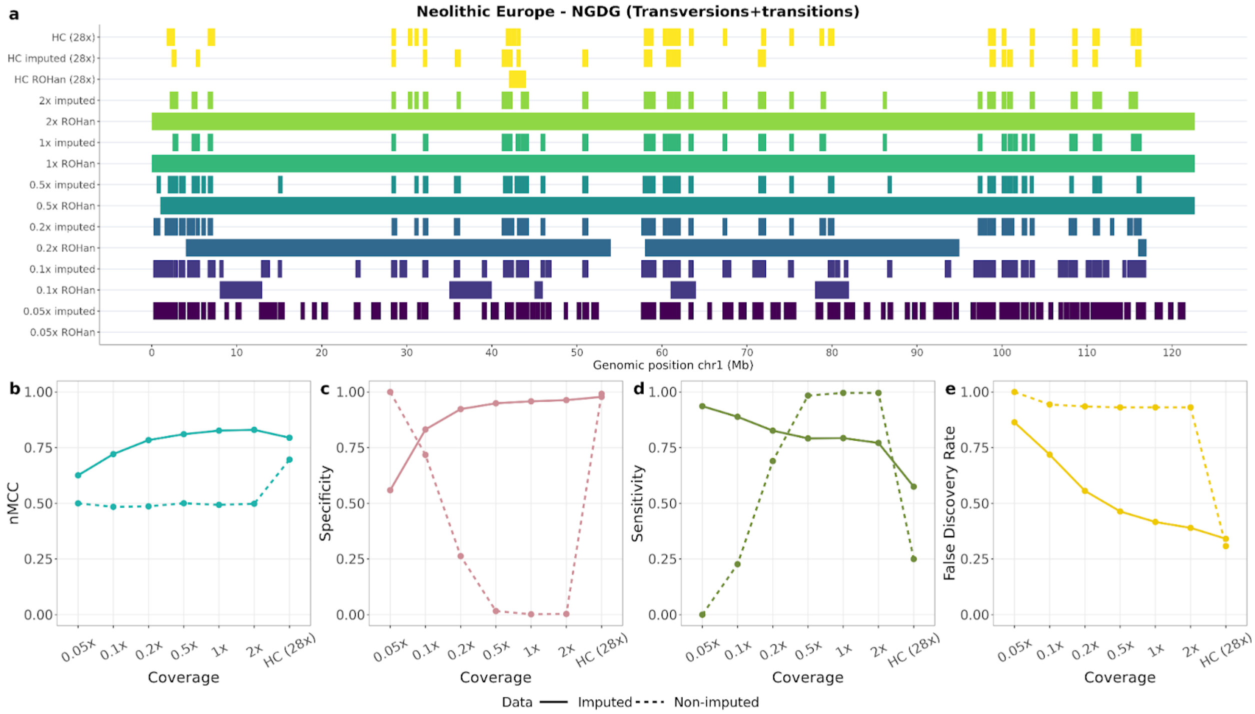

Fig. S45: *a) ROH called for the Newgrange Neolithic European dog across the high coverage and downsampled coverages (imputed and non-imputed) on chromosome one. b) Normalised Matthew correlation coefficient (nMCC), c) specificity, d) sensitivity and e) false discovery rate estimates based on the ROH inferred using the imputed (PLINK) or non-imputed (ROHan) samples, across all chromosomes and tested coverages. HC: High coverage.*

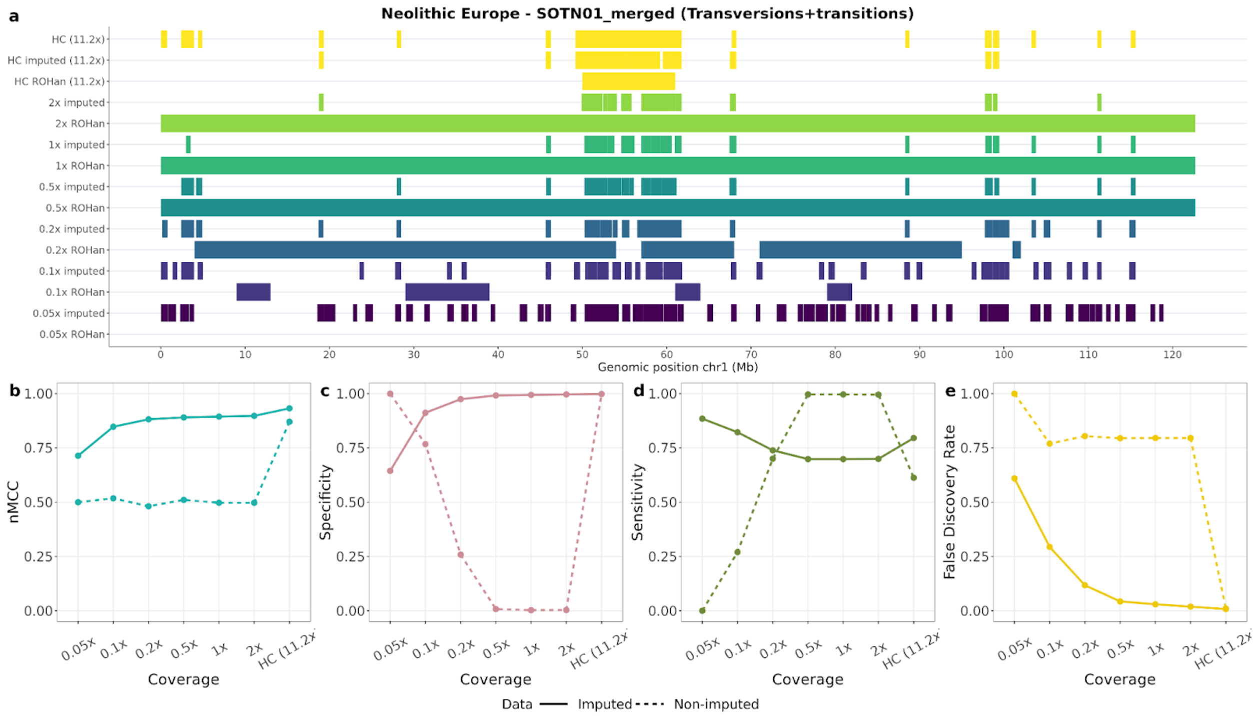

Fig. S46: *a) ROH called for the SOTN01 Neolithic European dog across the high coverage and downsampled coverages (imputed and non-imputed) on chromosome one. b) Normalised Matthew correlation coefficient (nMCC), c) specificity, d) sensitivity and e) false discovery rate estimates based on the ROH inferred using the imputed (PLINK) or non-imputed (ROHan) samples, across all chromosomes and tested coverages. HC: High coverage*

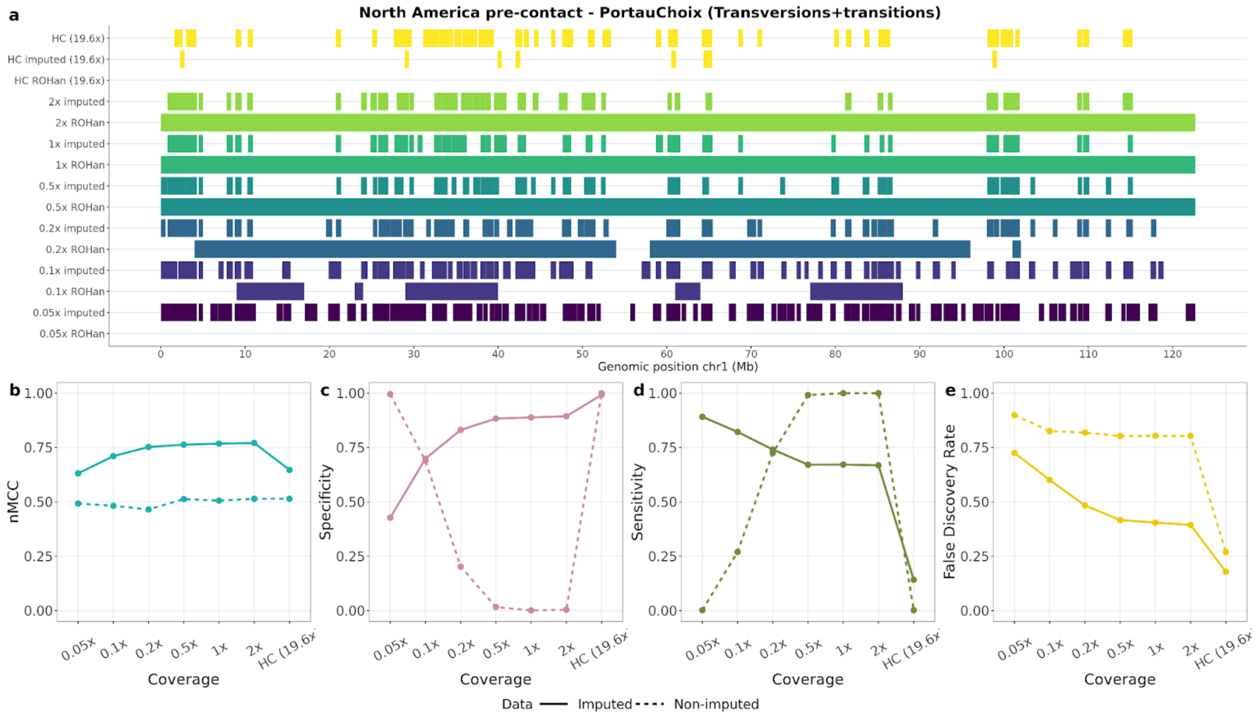

Fig. S47: *a) ROH called for the Port au Choix North American pre-contact dog across the high coverage and downsampled coverages (imputed and non-imputed) on chromosome one. b) Normalised Matthew correlation coefficient (nMCC), c) specificity, d) sensitivity and e) false discovery rate estimates based on the ROH inferred using the imputed (PLINK) or non-imputed (ROHan) samples, across all chromosomes and tested coverages. HC: High coverage*

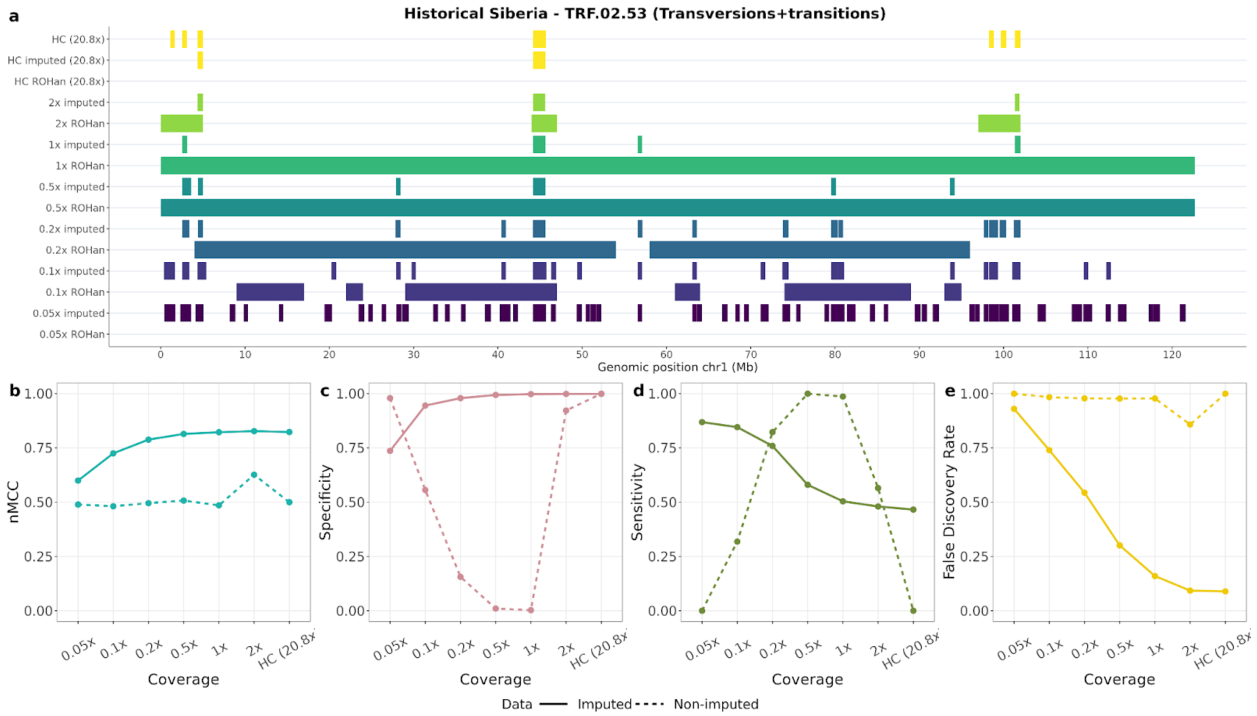

Fig. S48: *a) ROH called for the TRF.02.53 historical Siberian dog across the high coverage and downsampled coverages (imputed and non-imputed) on chromosome one. b) Normalised Matthew correlation coefficient (nMCC), c) specificity, d) sensitivity and e) false discovery rate estimates based on the ROH inferred using the imputed (PLINK) or non-imputed (ROHan) samples, across all chromosomes and tested coverages. HC: High coverage*

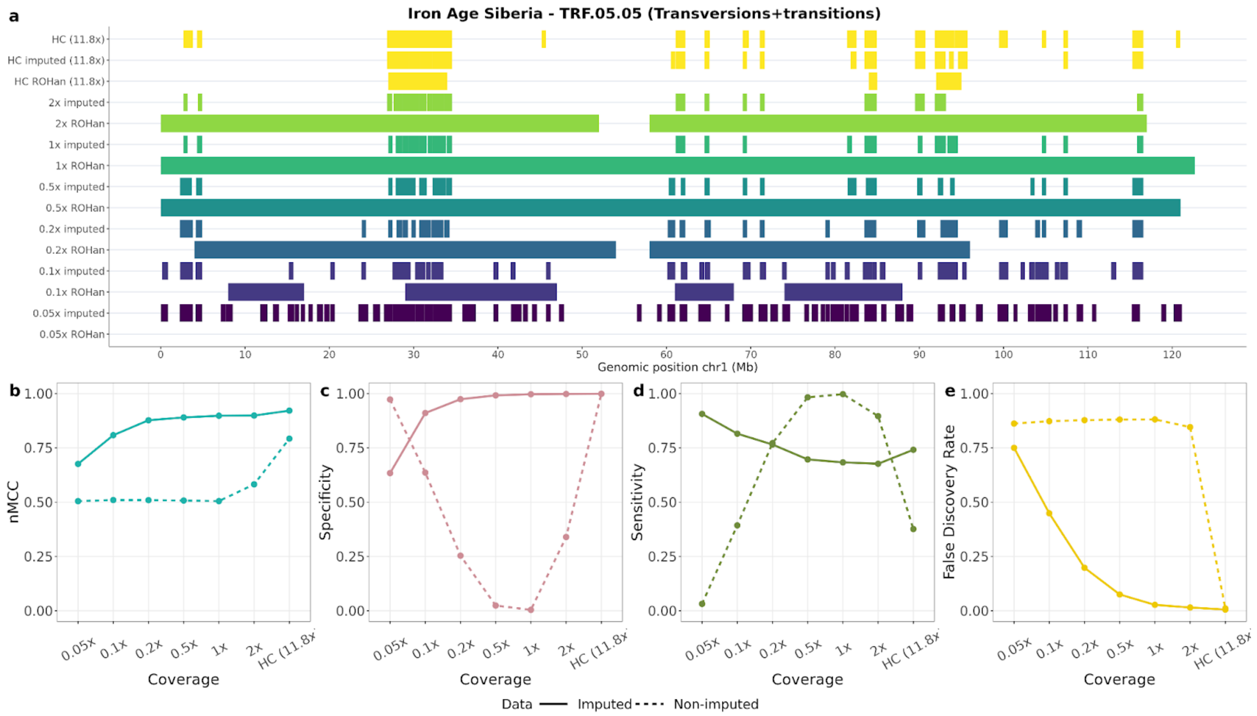

Fig. S49: *a) ROH called for the TRF.05.05 Iron Age Siberian dog across the high coverage and downsampled coverages (imputed and non-imputed) on chromosome one. b) Normalised Matthew correlation coefficient (nMCC), c) specificity, d) sensitivity and e) false discovery rate estimates based on the ROH inferred using the imputed (PLINK) or non-imputed (ROHan) samples, across all chromosomes and tested coverages. HC: High coverage*

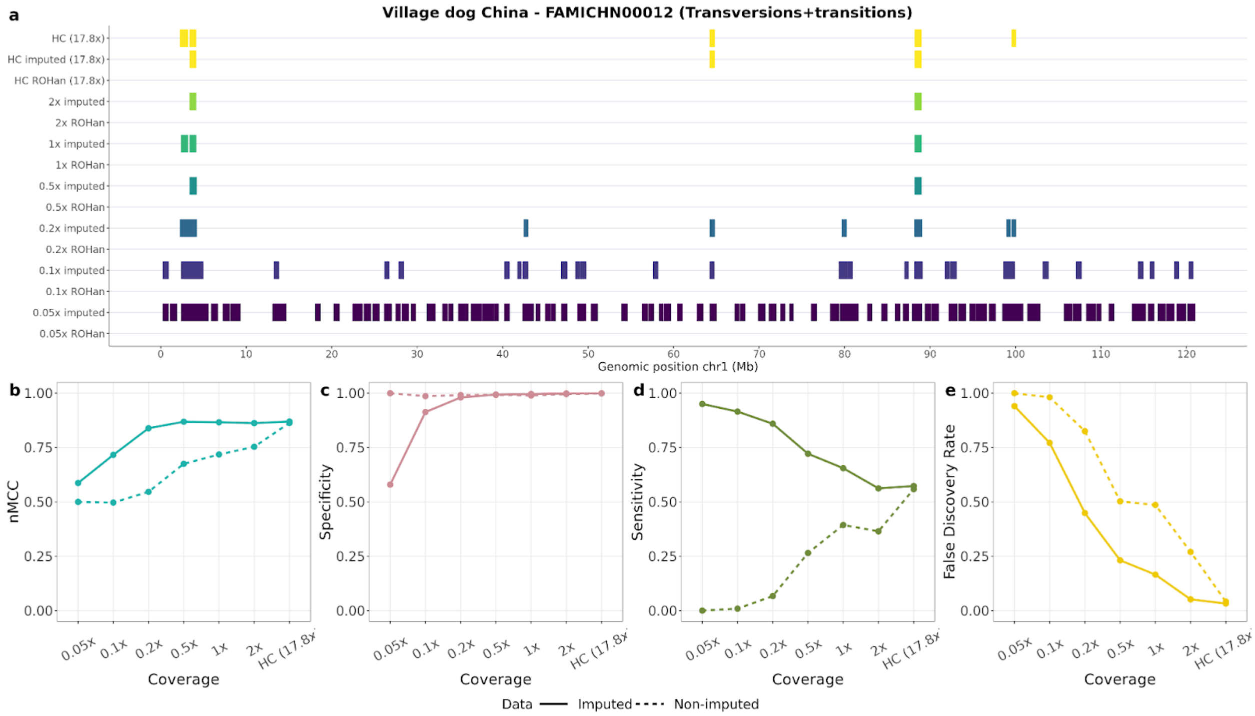

Fig. S50: *a) ROH called for the FAMICHN00012 Chinese Village dog across the high coverage and downsampled coverages (imputed and non-imputed) on chromosome one. b) Normalised Matthew correlation coefficient (nMCC), c) specificity, d) sensitivity and e) false discovery rate estimates based on the ROH inferred using the imputed (PLINK) or non-imputed (ROHan) samples, across all chromosomes and tested coverages. HC: High coverage*

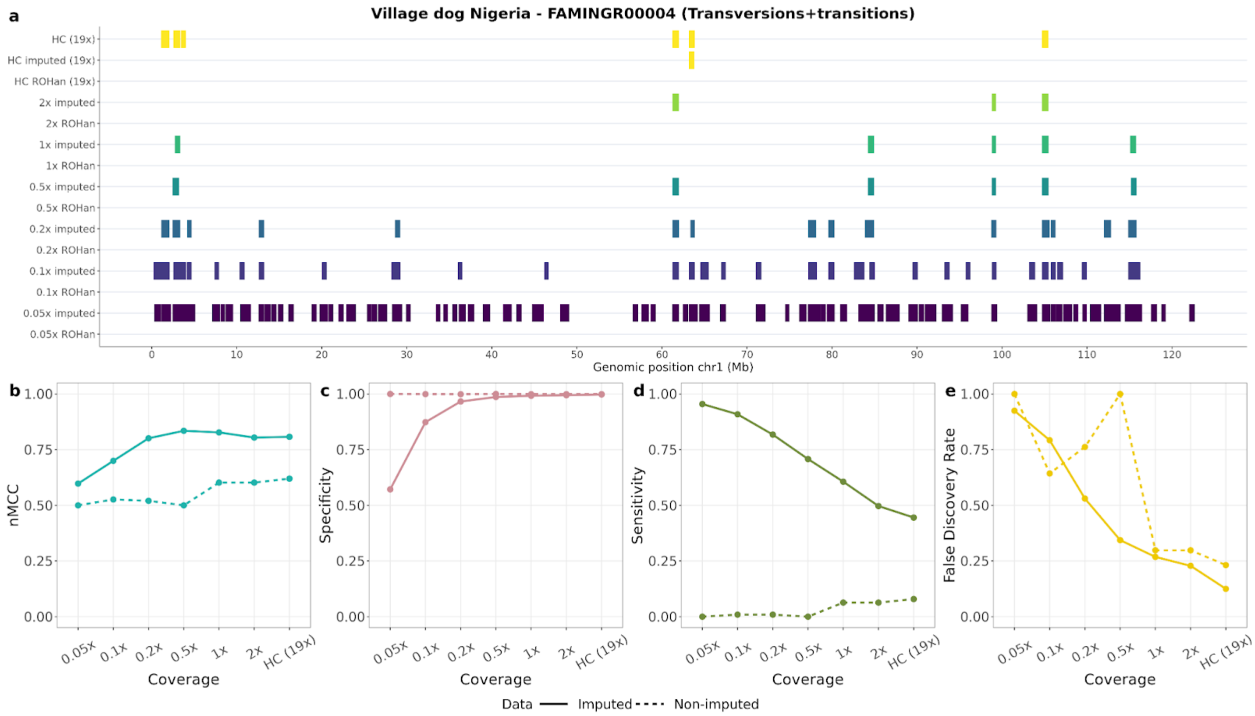

Fig. S51: *a) ROH called for the FAMINGR00004 Nigerian Village dog across the high coverage and downsampled coverages (imputed and non-imputed) on chromosome one. b) Normalised Matthew correlation coefficient (nMCC), c) specificity, d) sensitivity and e) false discovery rate estimates based on the ROH inferred using the imputed (PLINK) or non-imputed (ROHan) samples, across all chromosomes and tested coverages. HC: High coverage*

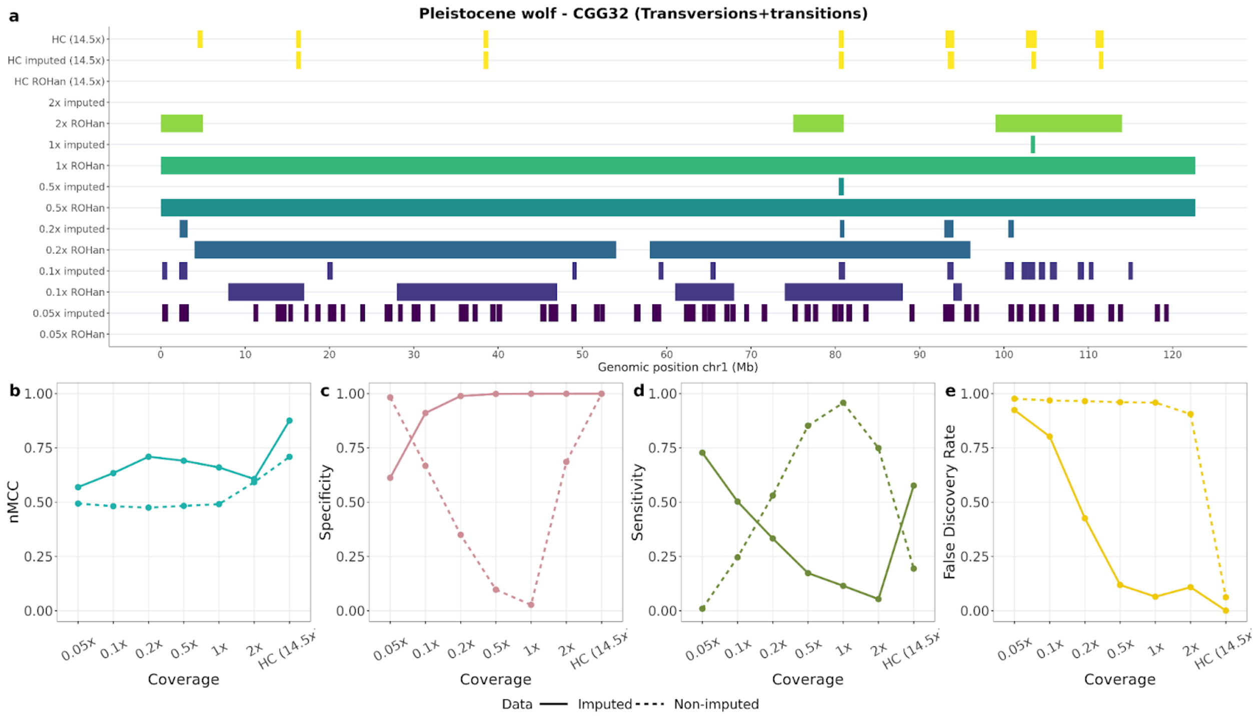

Fig. S52: *a) ROH called for the CGG32 Pleistocene wolf across the high coverage and downsampled coverages (imputed and non-imputed) on chromosome one. b) Normalised Matthew correlation coefficient (nMCC), c) specificity, d) sensitivity and e) false discovery rate estimates based on the ROH inferred using the imputed (PLINK) or non-imputed (ROHan) samples, across all chromosomes and tested coverages. HC: High coverage*

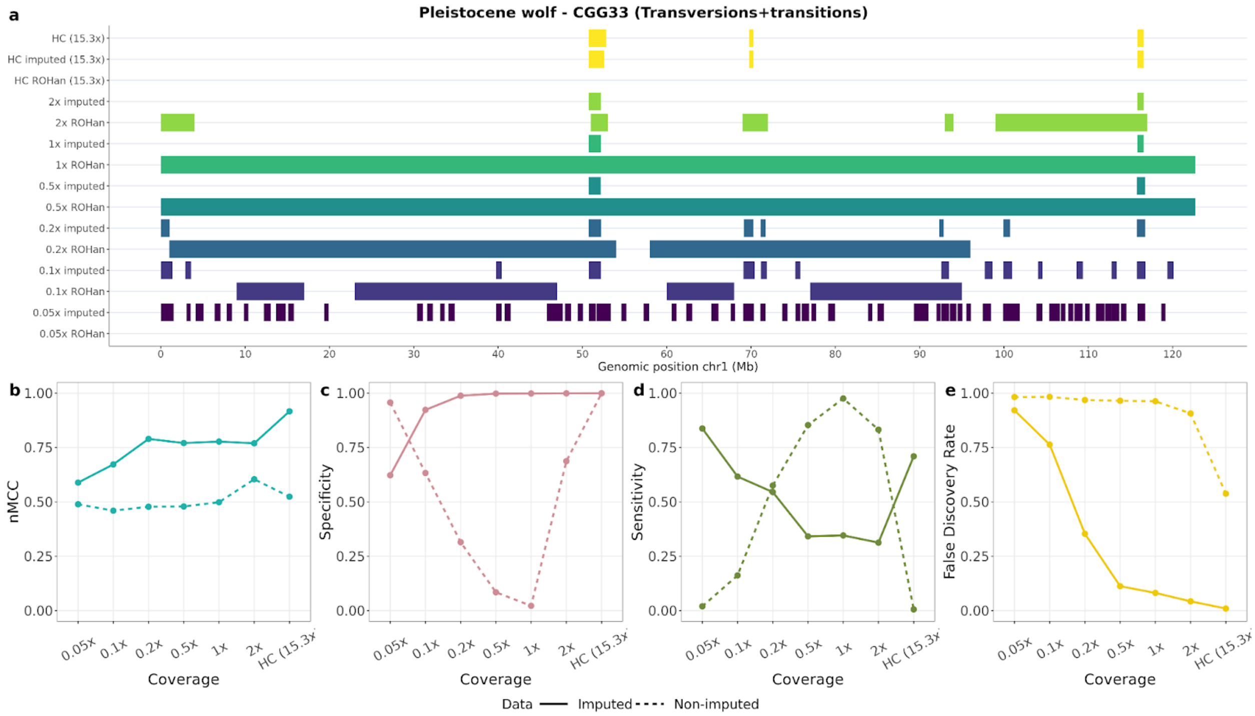

Fig. S53: *a) ROH called for the CGG33 Pleistocene wolf across the high coverage and downsampled coverages (imputed and non-imputed) on chromosome one. b) Normalised Matthew correlation coefficient (nMCC), c) specificity, d) sensitivity and e) false discovery rate estimates based on the ROH inferred using the imputed (PLINK) or non-imputed (ROHan) samples, across all chromosomes and tested coverages. HC: High coverage*

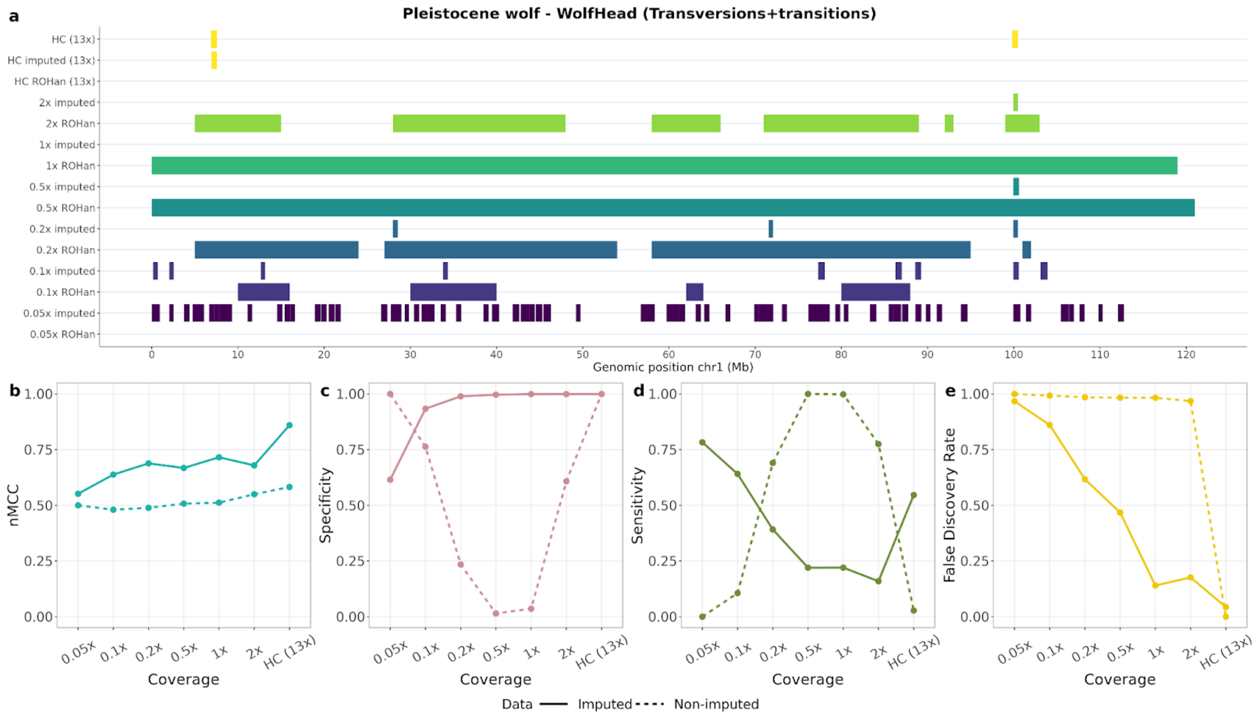

Fig. S54: *a) ROH called for the WolfHead Pleistocene wolf across the high coverage and downsampled coverages (imputed and non-imputed) on chromosome one. b) Normalised Matthew correlation coefficient (nMCC), c) specificity, d) sensitivity and e) false discovery rate estimates based on the ROH inferred using the imputed (PLINK) or non-imputed (ROHan) samples, across all chromosomes and tested coverages. HC: High coverage*

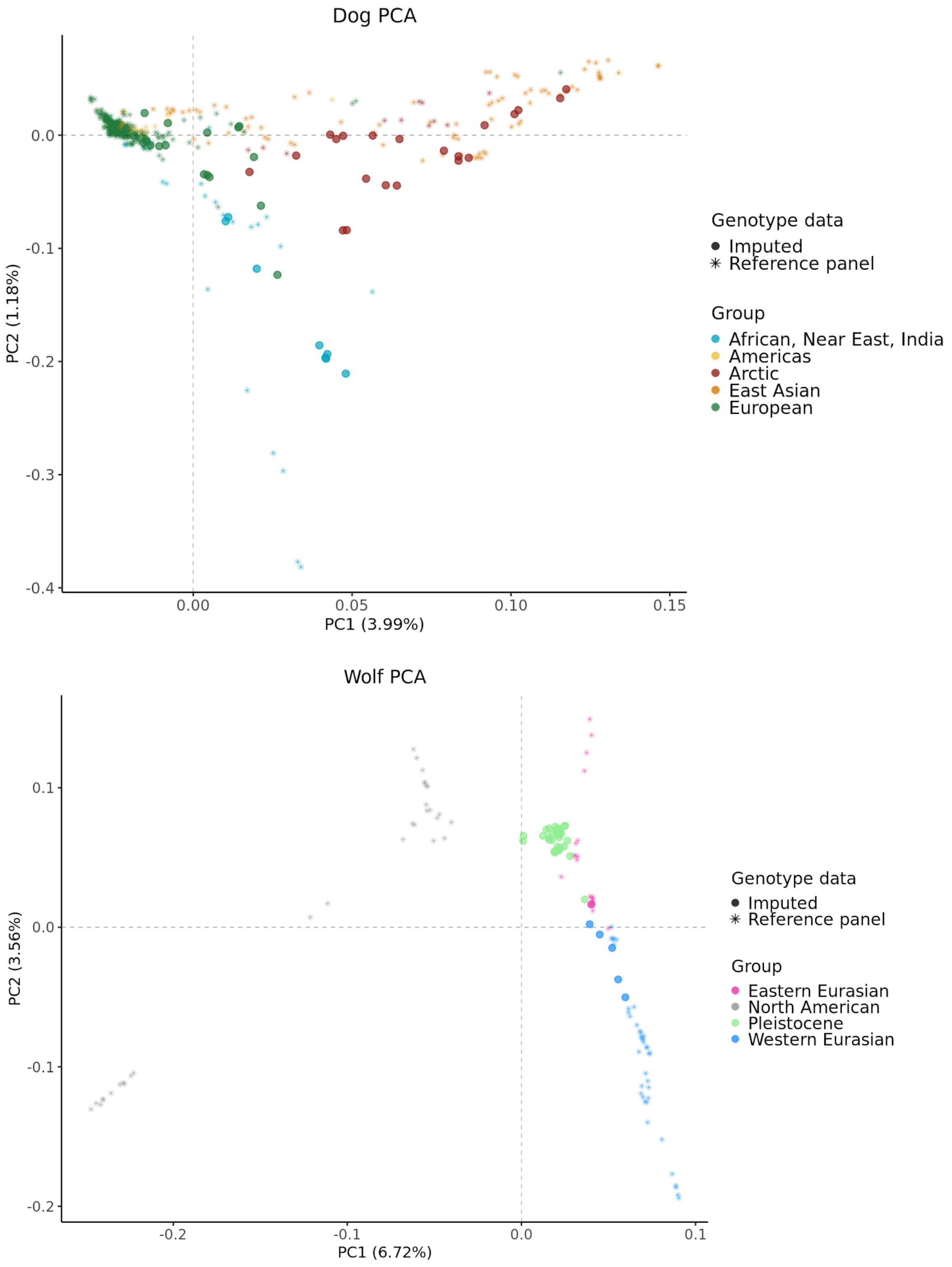

Fig. S55: *PCA of the imputed dogs (top plot) and wolves (bottom plot), alongside the dog or wolf samples from the reference panel. Both imputed and modern samples were used to create the PCs.*

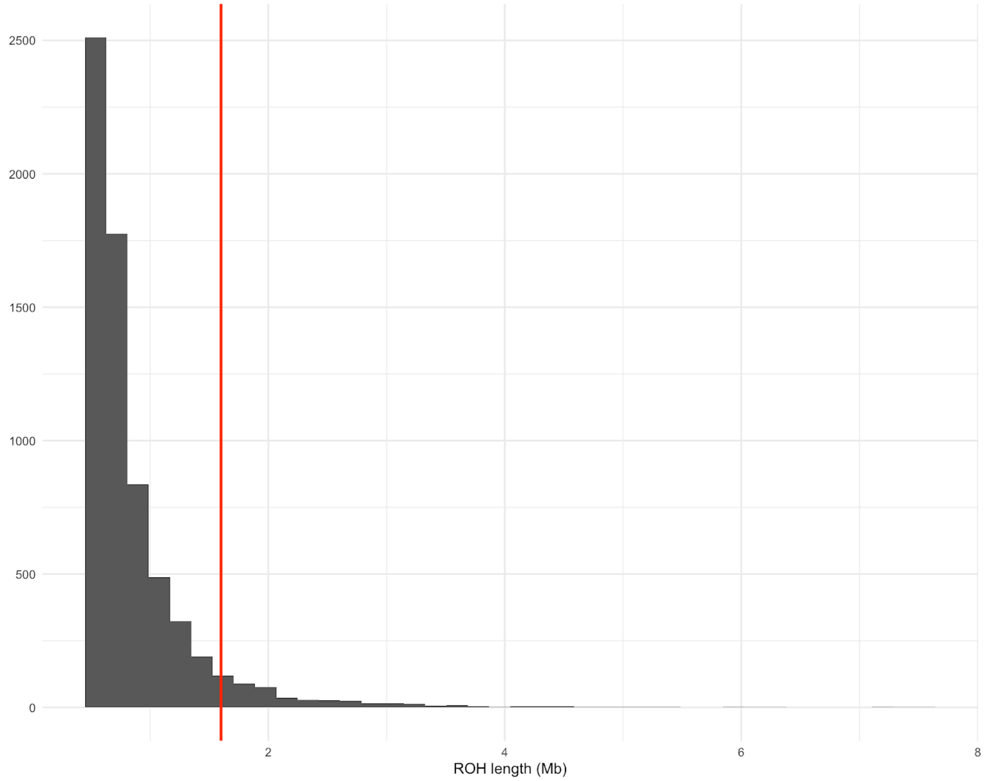

Fig. S56: *Histogram of ROH lengths of imputed ancient dog samples. Red line is the 1.6Mb cutoff used to categorise short (<1.6Mb) and long (≥1.6Mb) ROH.*

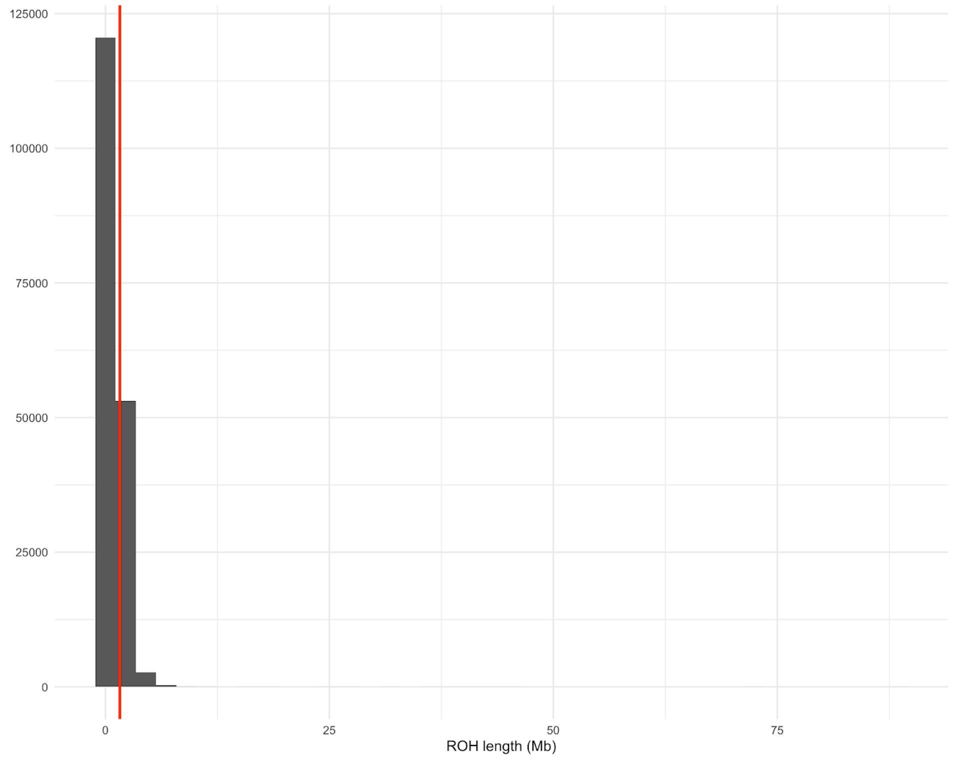

Fig. S57: *Histogram of ROH lengths of modern dog samples. Red line is the 1.6Mb cutoff used to categorise short (<1.6Mb) and long (≥1.6Mb) ROH.*

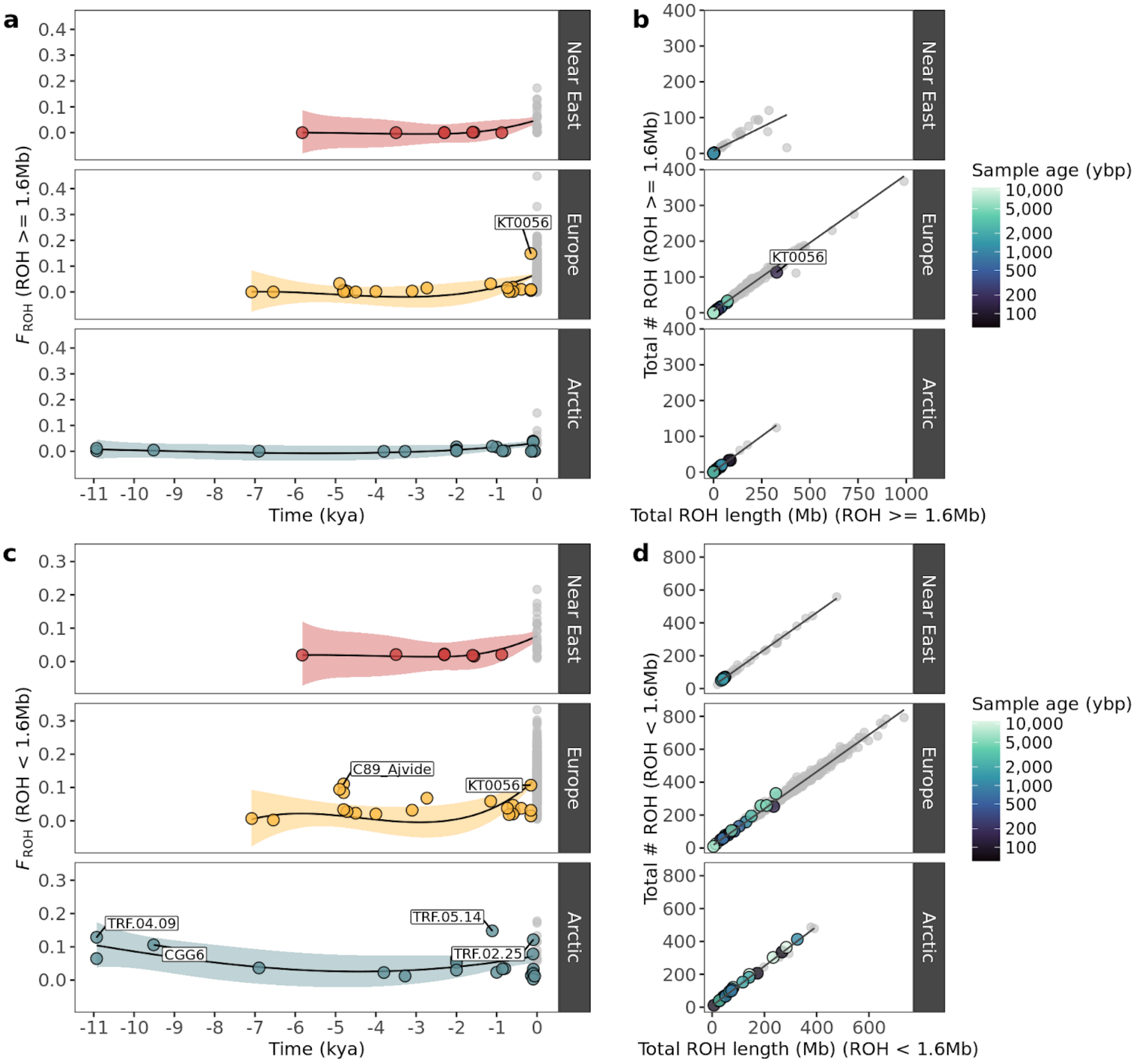

Fig. S58: *Left plots: Genomic inbreeding coefficient (FROH) of imputed and modern dogs as a function of time for a) ROH≥1.6Mb and c) ROH<1.6Mb. Imputed samples are coloured based on their geographic grouping, while modern samples are coloured in grey. Samples with F_{ROH} values above 0.1 are indicated. Right plots: Total number of ROH segments plotted against total ROH length for the imputed dogs for b) ROH ≥ 1.6Mb and d) ROH < 1.6Mb. Colours correspond to age of imputed samples in years before present, while modern samples belonging to each dog group are coloured in grey.*

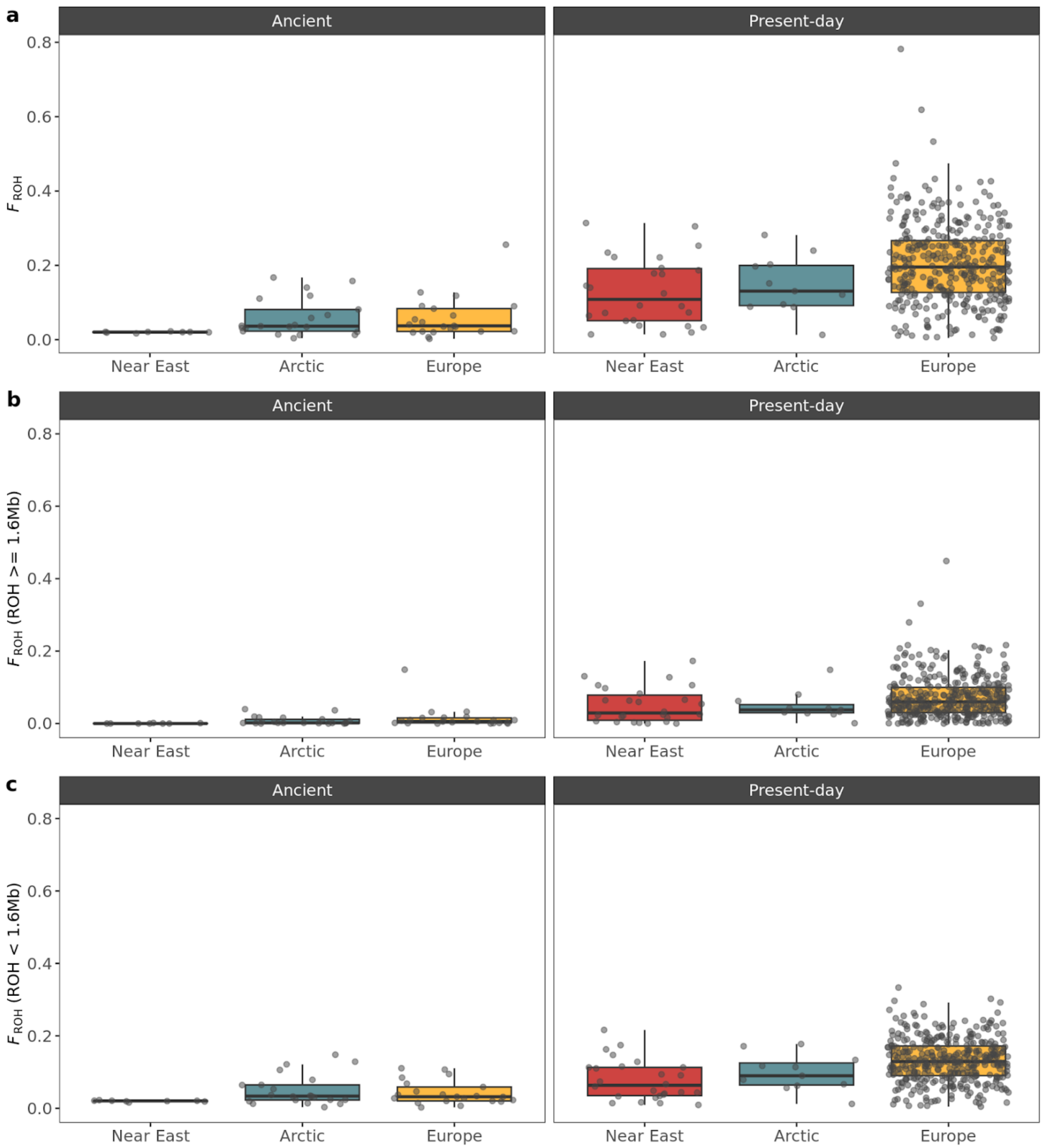

Fig. S59: *Boxplots of genomic inbreeding coefficients (FROH) from ancient and modern dogs for three ancestral groups for a) all ROH, b) ROH ≥ 1.6Mb and c) ROH < 1.6Mb. Horizontal lines represent the median.*

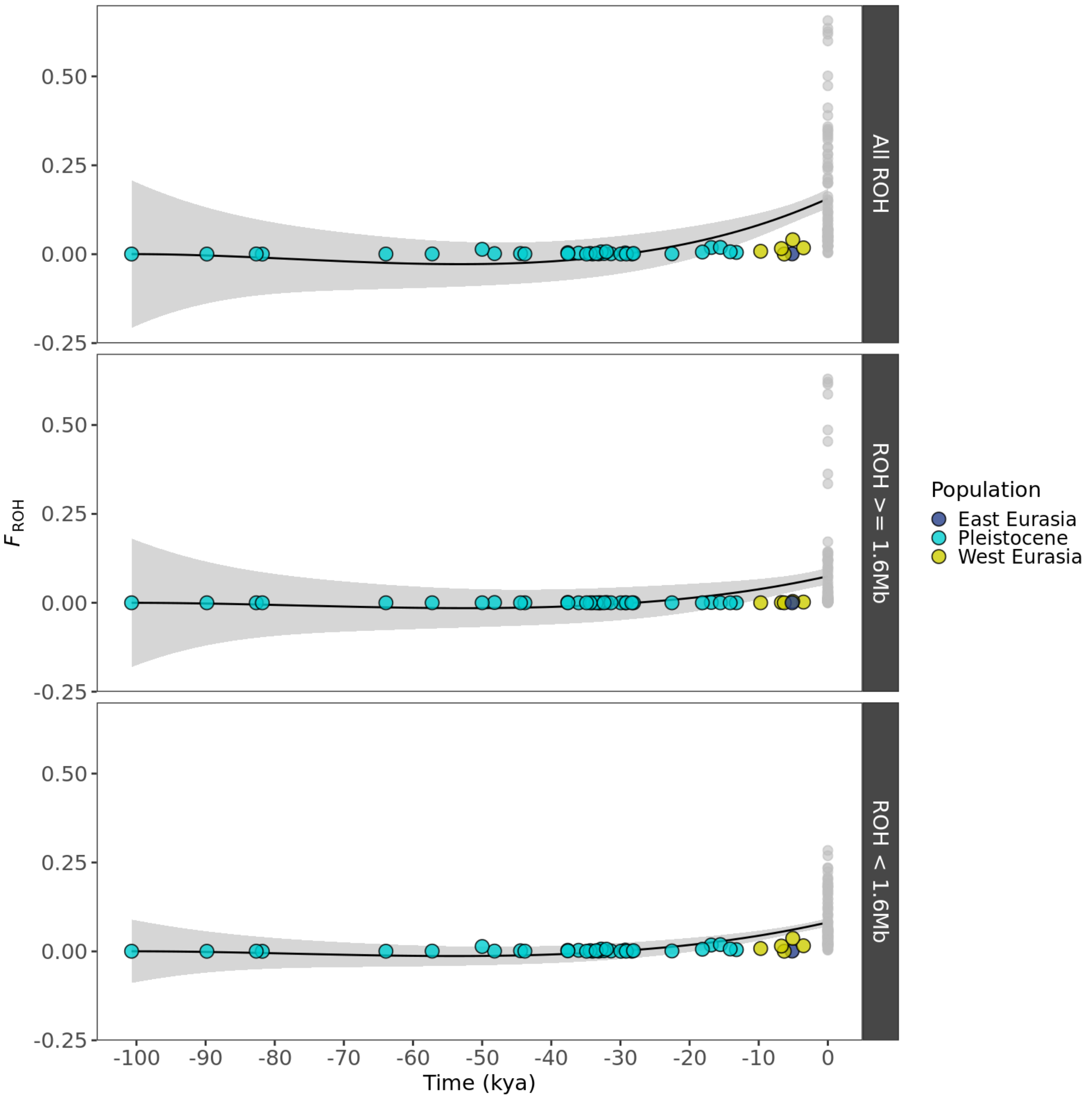

Fig. S60: *Genomic inbreeding coefficient (FROH) of imputed and modern wolves plotted as a function of time for all ROH, ROH ≥ 1.6Mb and ROH < 1.6Mb. Imputed samples are coloured based on their geographic grouping, while modern samples are coloured in grey.*

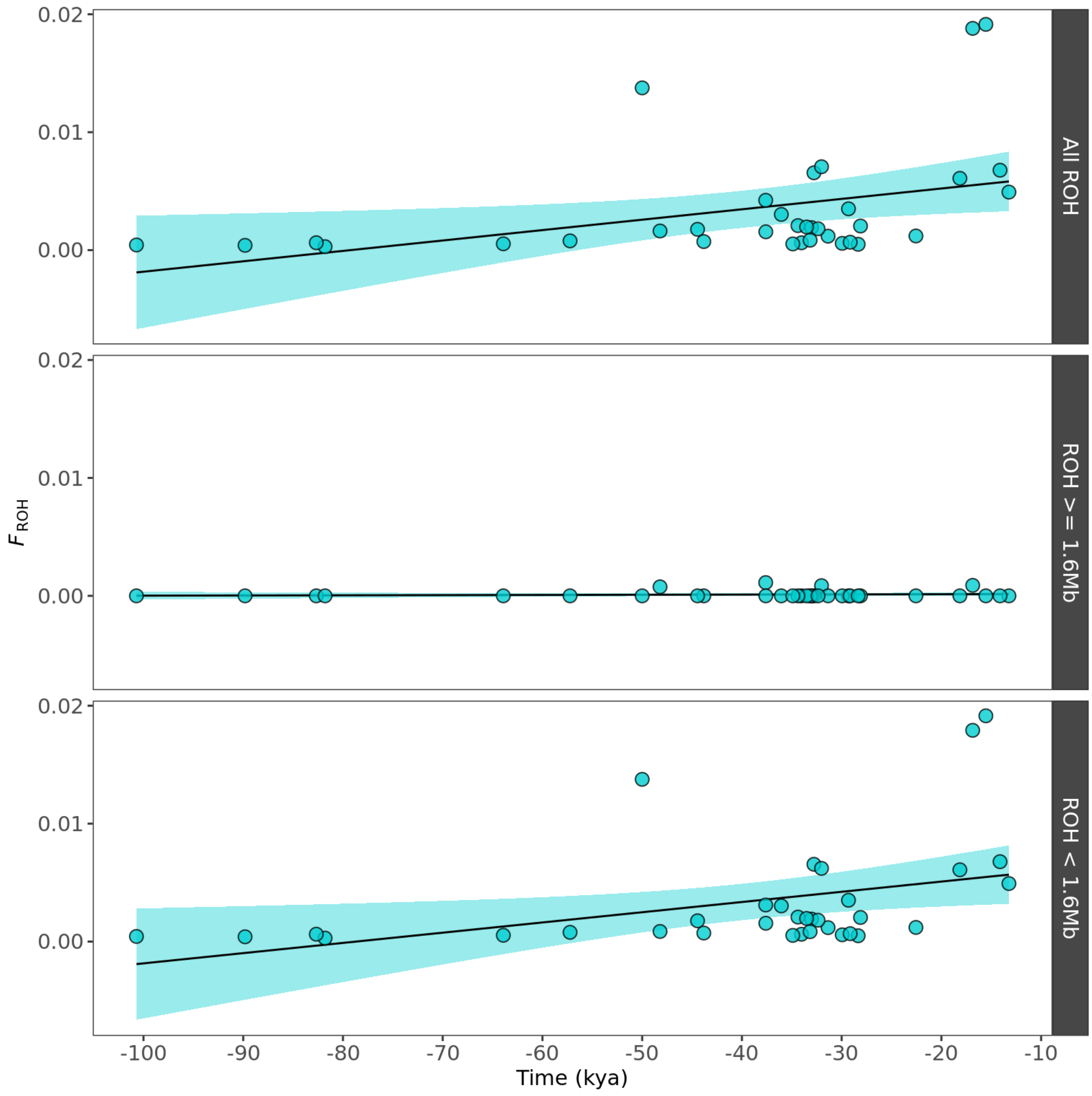

Fig. S61: *Genomic inbreeding coefficient (FROH) of imputed Pleistocene wolves plotted as a function of time for i) all ROH, ii) ROH ≥ 1.6Mb and iii) ROH < 1.6Mb.*

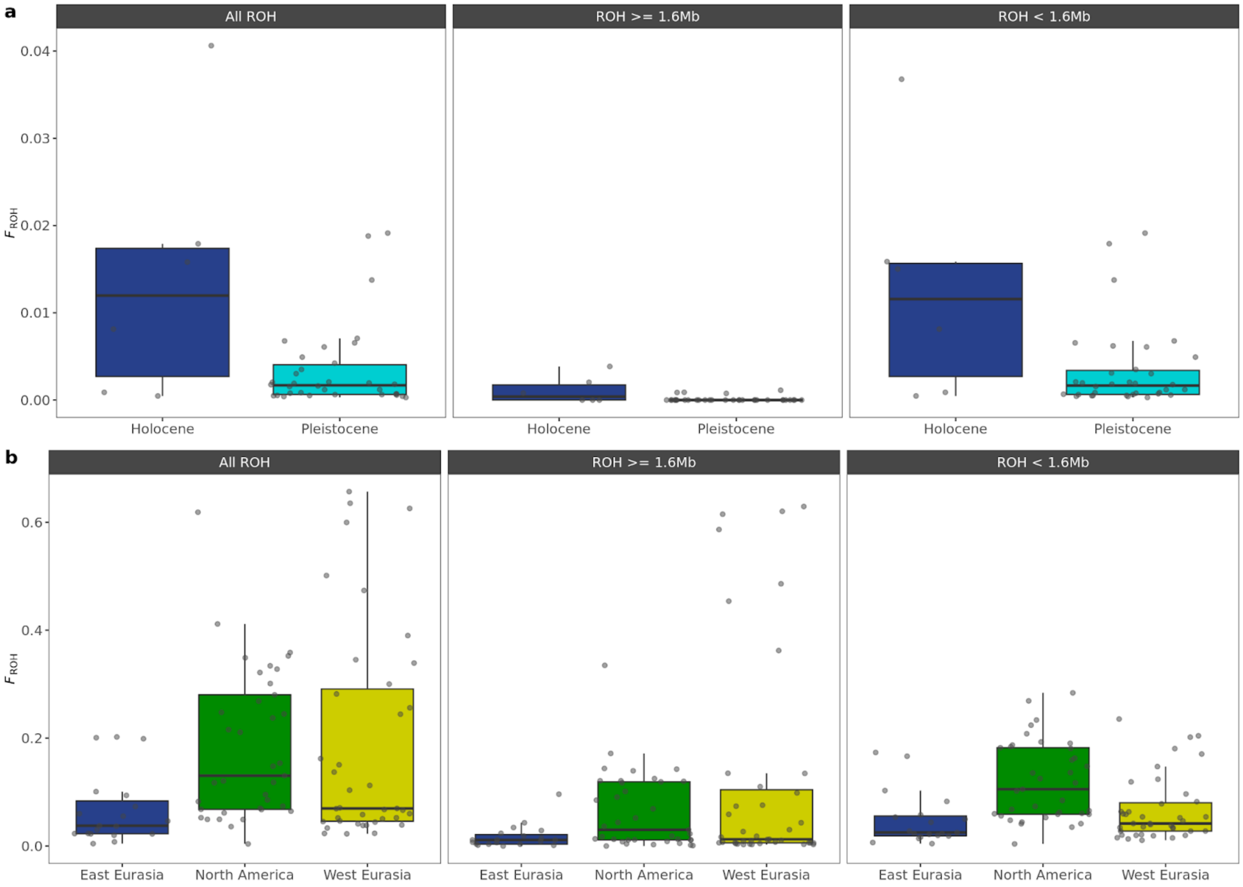

Fig. S62: *Boxplots of genomic inbreeding coefficients (FROH) from a) ancient and b) present-day wolves for all ROH, ROH ≥ 1.6Mb and ROH < 1.6Mb. Horizontal lines represent the median.*

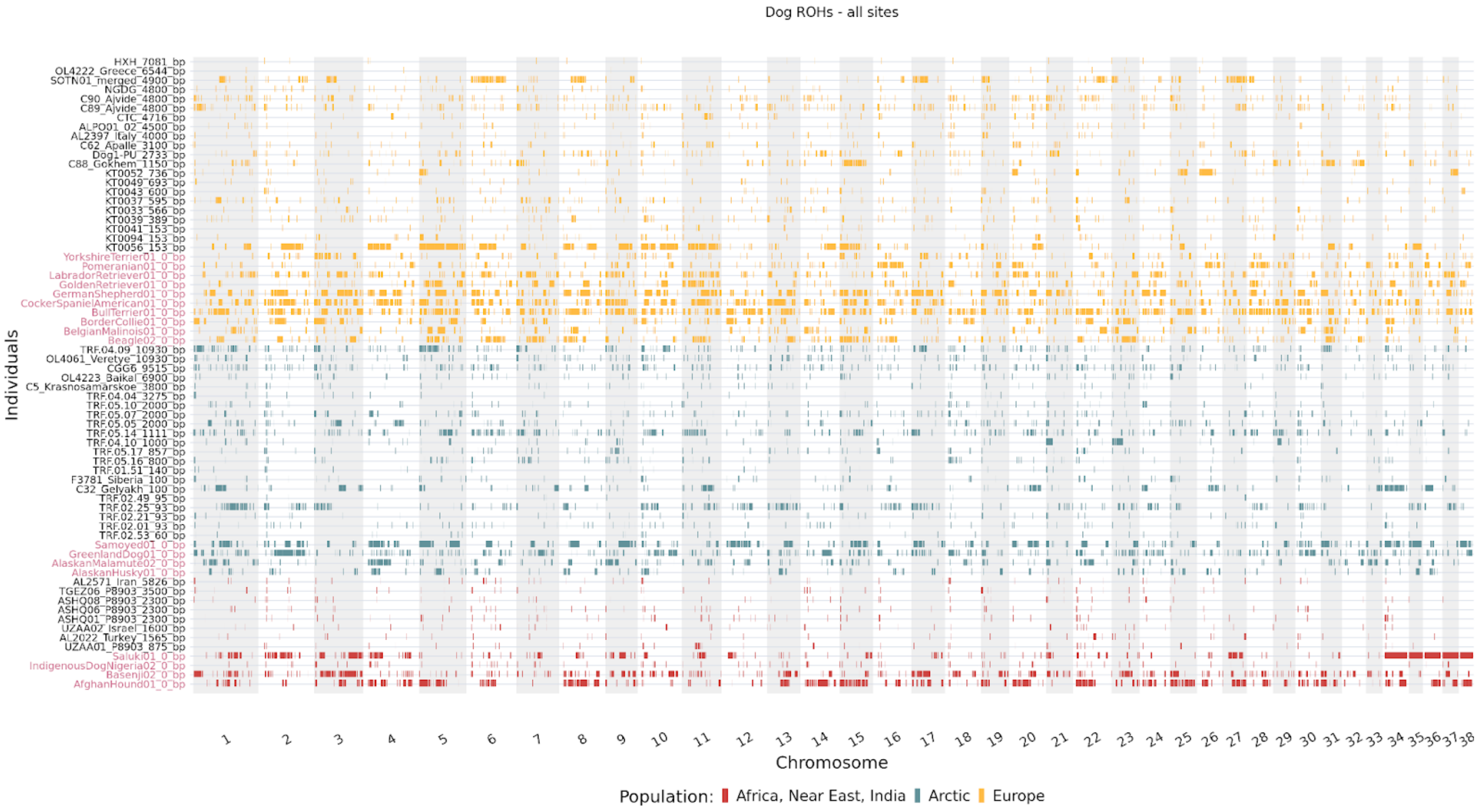

Fig. S63: *ROH across the genome for each imputed dog sample estimated based on transversions and transitions. ROH bands are coloured based on geographic origin and ordered based on age within each geographic region. ROH for a subset of modern samples are added for comparison and highlighted with red text.*

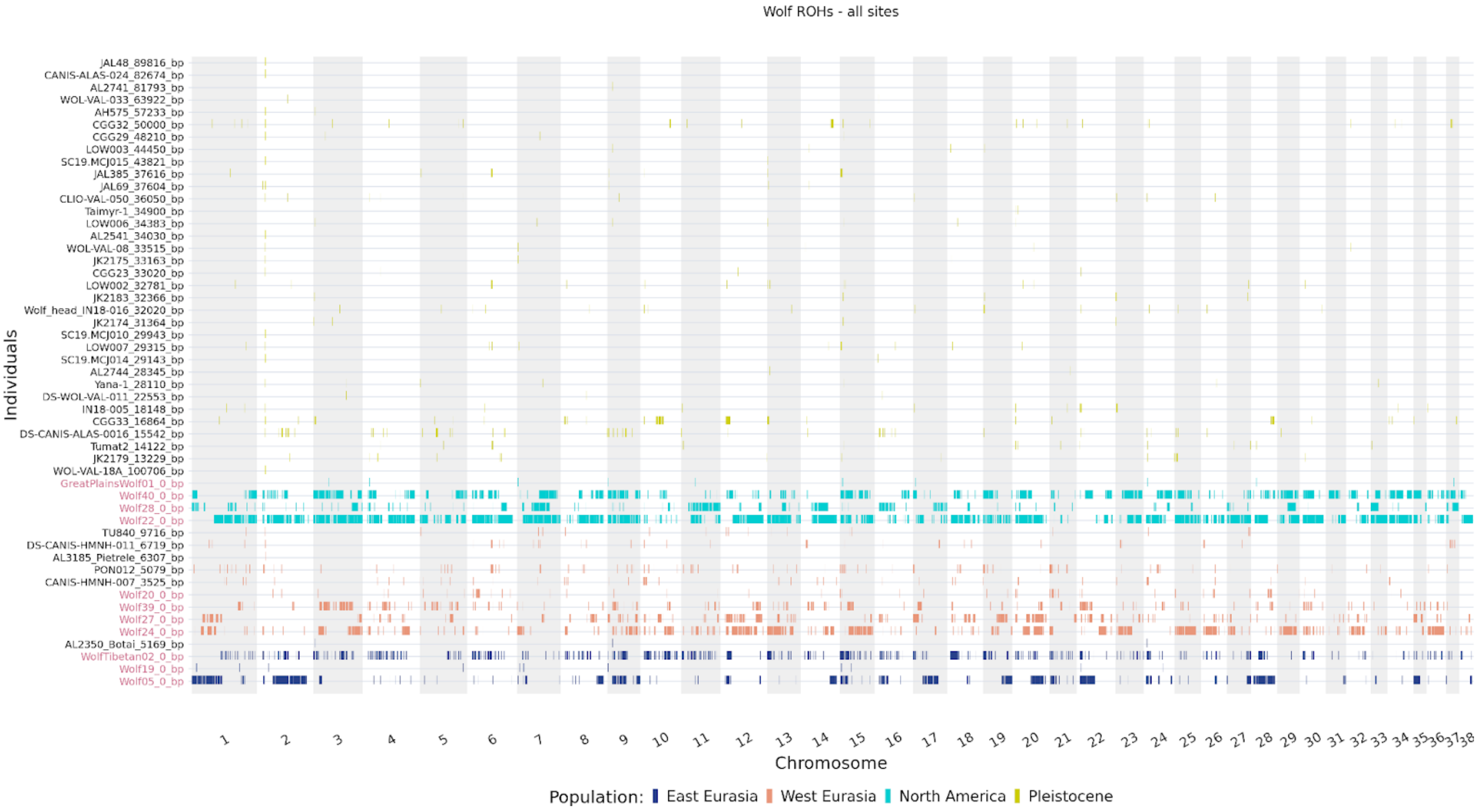

Fig. S64: *ROH across the genome for each imputed wolf sample estimated based on transversions and transitions. ROH bands are coloured based on geographic origin and ordered based on age within each geographic region. ROH for a subset of modern samples are also added for comparison and highlighted with red text.*

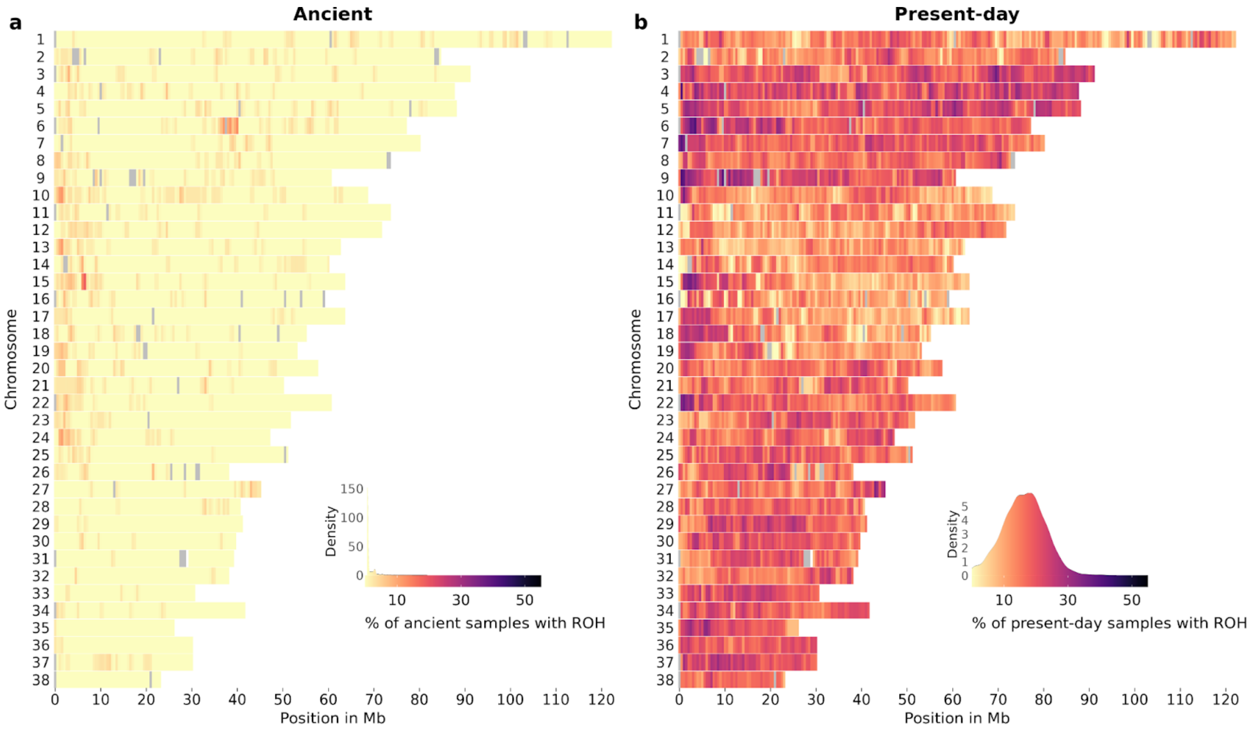

Fig. S65: *ROH across all chromosomes of a) ancient wolves and b) present-day wolves. The colour legend represents the % of samples which have an ROH at each genomic position, with more yellow regions representing ROH deserts and more purple regions representing ROH islands. Grey coloured regions indicate windows with an average depth of coverage estimated from all ancient dog samples above or below the mean ± 2*std.*

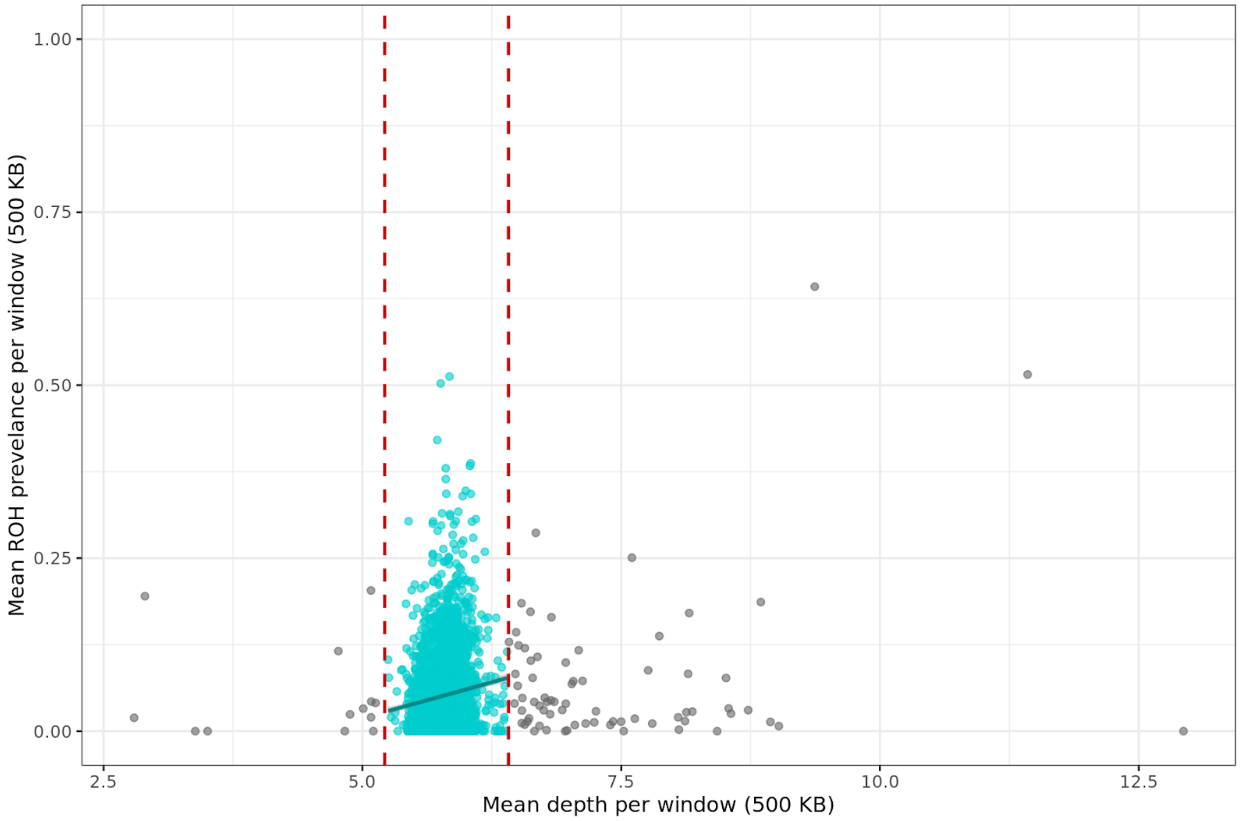

Fig. S66: *Mean ROH prevalence per 500Kb genomic window against the mean depth of each window estimated from 50 ancient dogs. Windows with depth above or below the mean ± 2*std were not included in estimating ROH prevalence across the genome of ancient and modern dogs.*

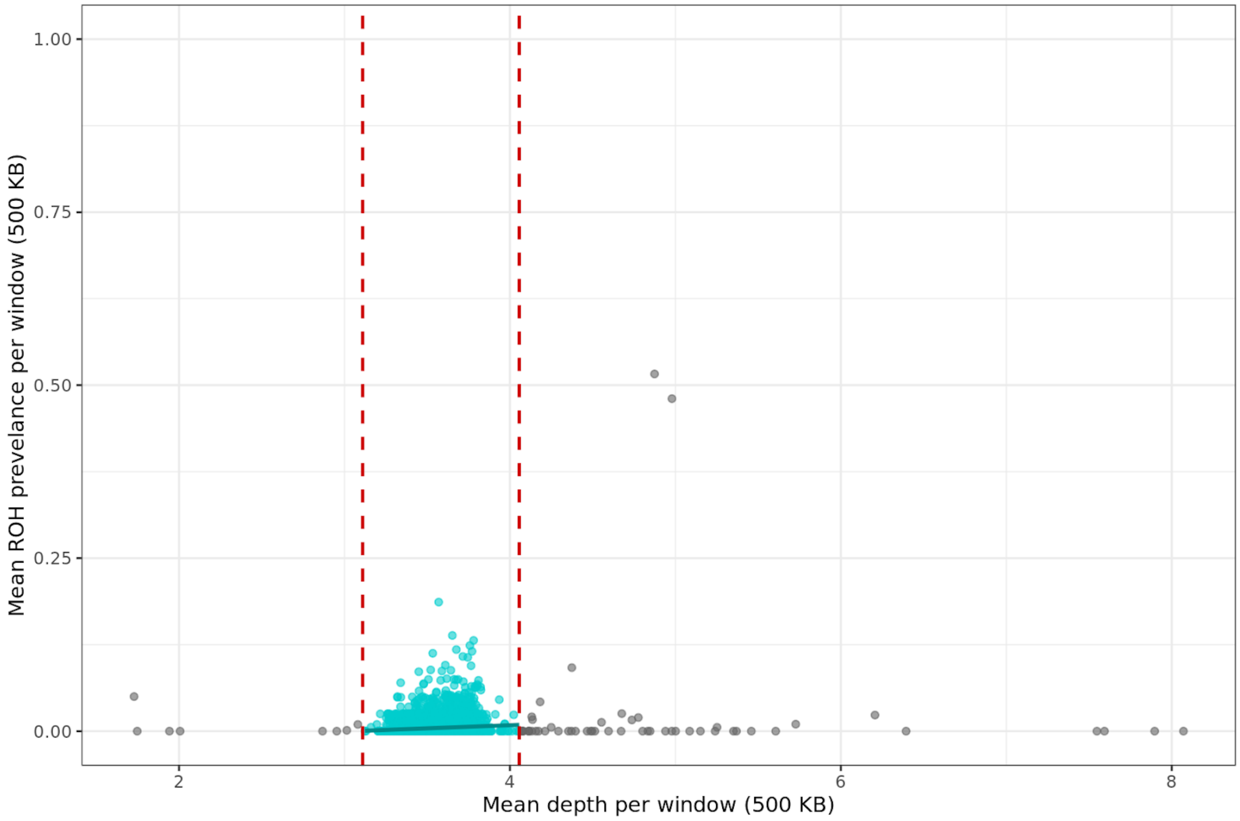

Fig. S67: *Mean ROH prevalence per 500Kb genomic window against the mean depth of each window estimated from 40 ancient wolves. Windows with depth above or below the mean ± 2*std were not included in estimating ROH prevalence across the genome of ancient and modern wolves.*

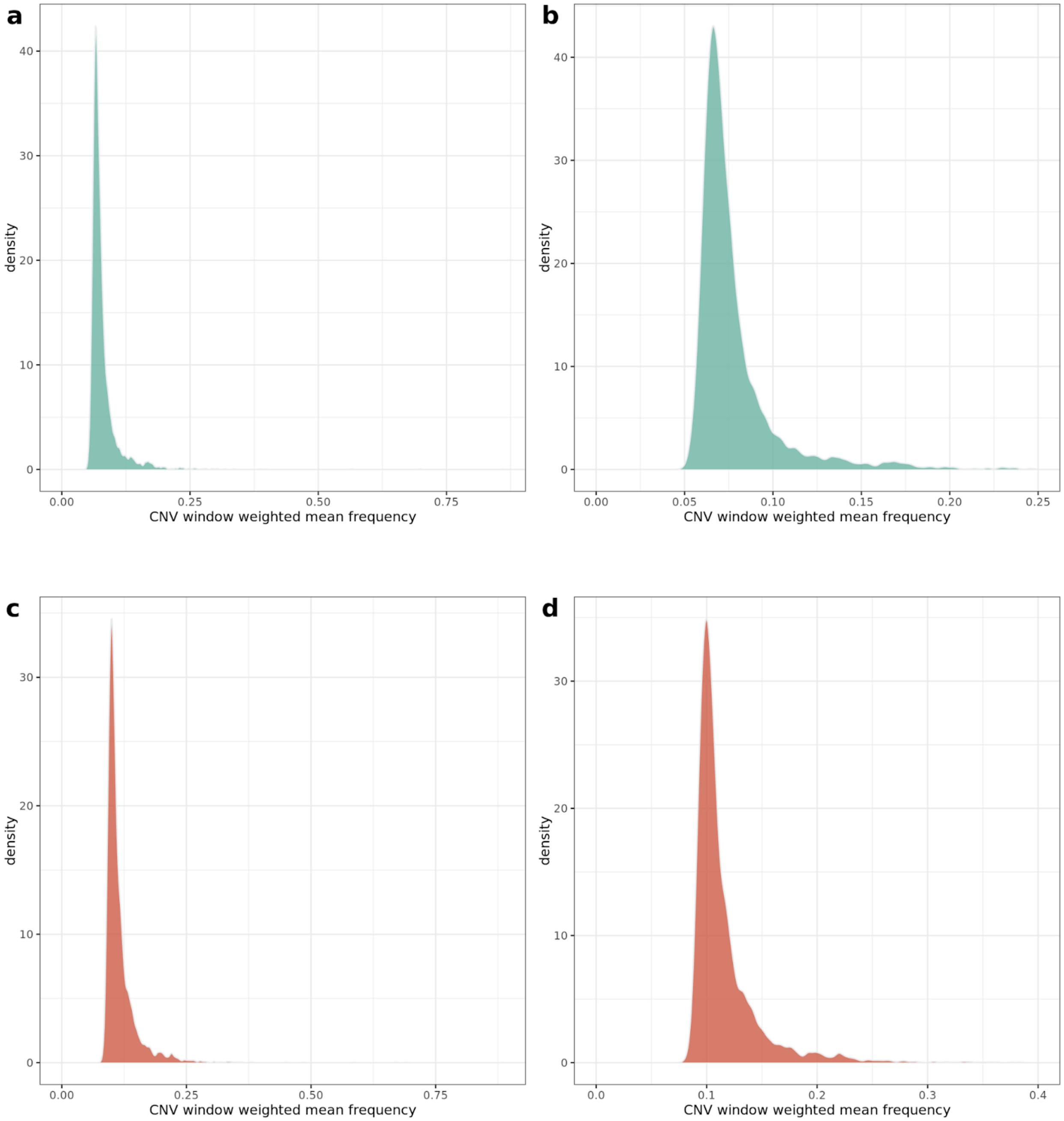

Fig. S68: *Density plots of the weighted mean frequency of CNV windows overlapping the 4,385 500Kb autosomal windows used in the ROH analysis for a, b) dogs and c, d) wolves. The two left panels (a,c) show the full distribution, whereas the two right panels (b,d)) show the distribution with an x-axis limit of 0.25 and 0.4 respectively.*

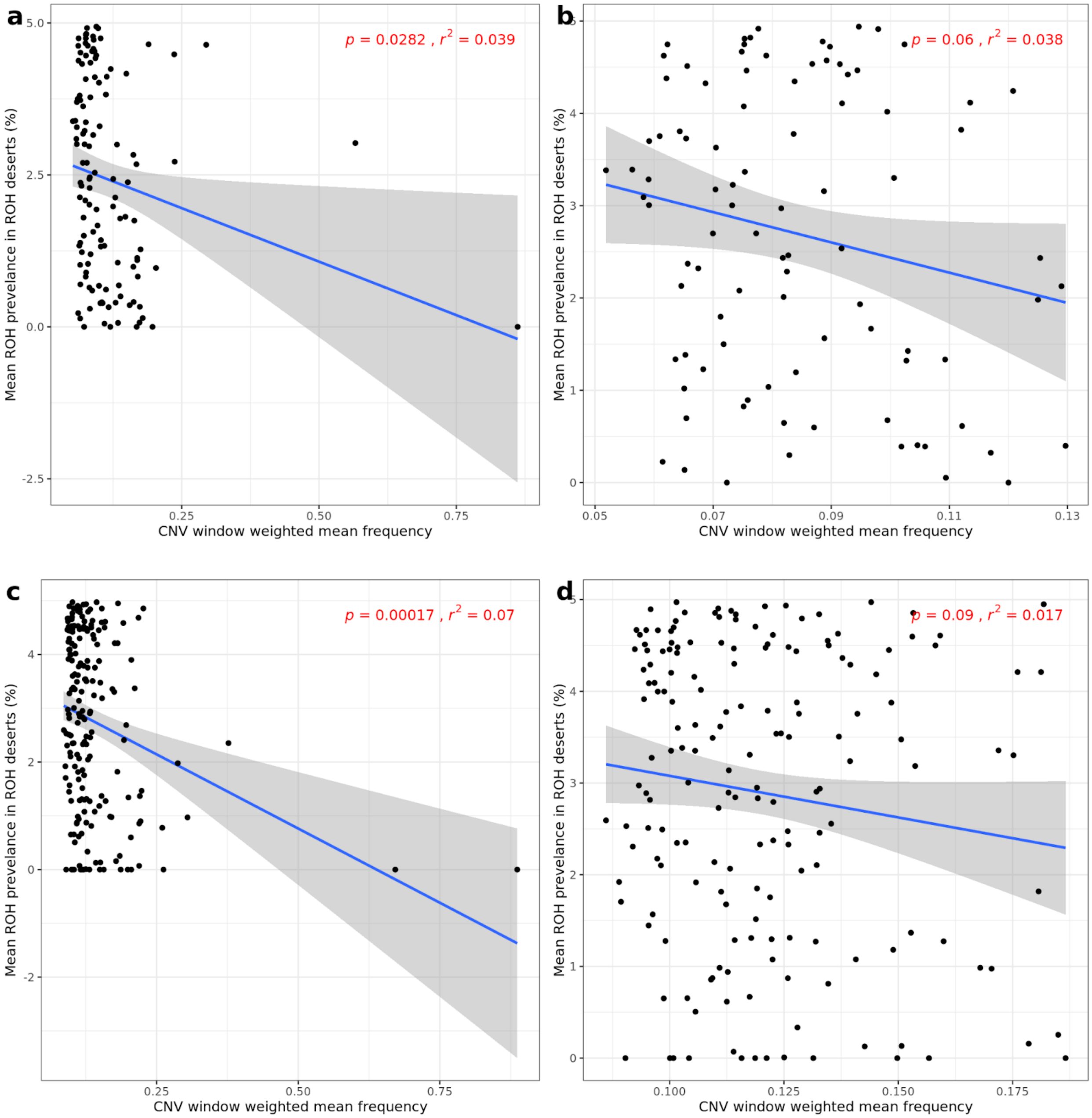

Fig. S69: *Correlation between mean ROH prevalence and the weighted mean frequency of CNV windows across our ROH deserts for a,b) modern dogs and c,d) modern wolves. The two left panels show a significant correlation when using all a) 124 and c) 209 identified ROH deserts for dogs and wolves. The two right panels show an insignificant correlation after removing ROH deserts above a CNV window weighted mean frequency cutoff of b) 0.13 for dogs and c) 0.19 for wolves. This resulted in a final set of 87 ROH desert windows for dogs and 171 for wolves.*

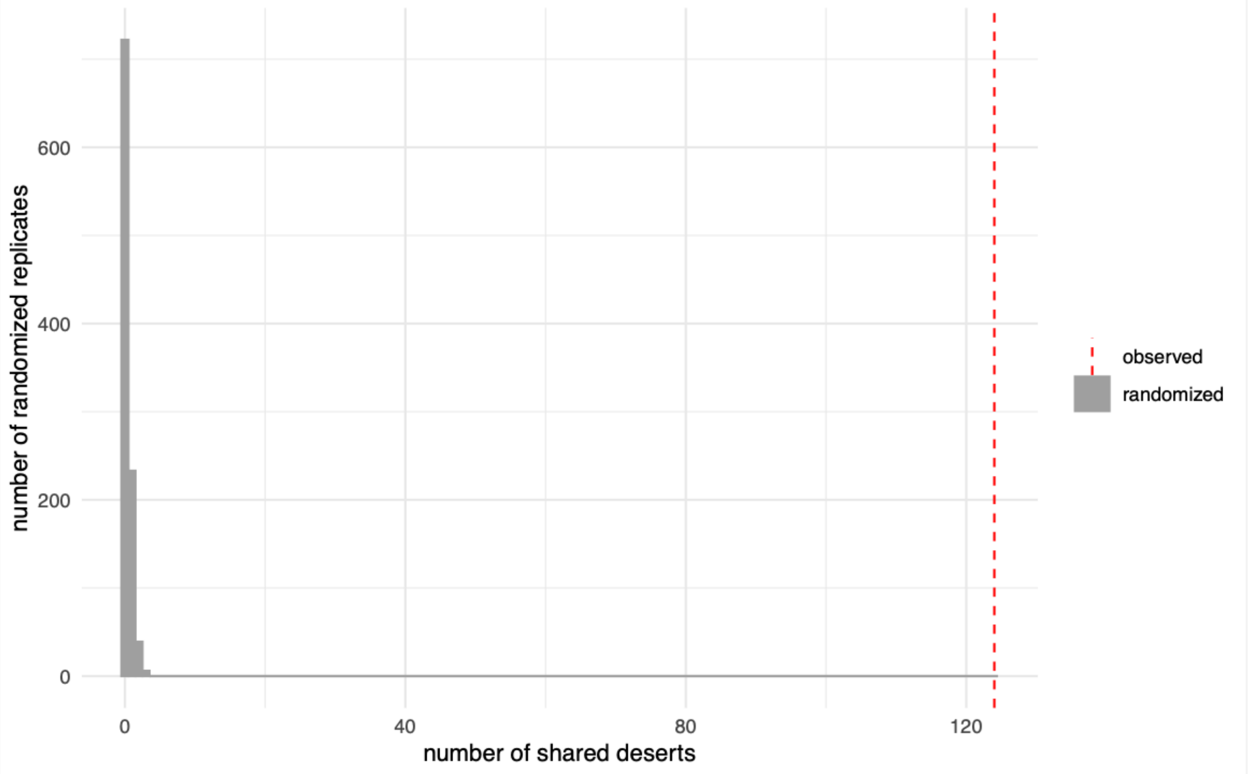

Fig. S70: *Distribution of the number of ROH desert windows shared between 52 ancient and 502 present-day dogs across 1,000 randomized replicates (grey histogram), with the observed count from the empirical data (vertical dashed red line). The observed number of shared ROH deserts exceeds that of all replicates, resulting in an empirical one-sided p-value effectively equal to zero.*

Dataset S1 (separate file). Metadata for the published and newly sequenced ancient samples used in this study.

Dataset S2 (separate file). Metadata for the samples included in the reference panel.

Dataset S3 (separate file). Metadata for the high coverage dog and wolf samples used for imputation benchmarking.

Dataset S4 (separate file). Mann–Whitney U tests for significant ROH differences between dog populations and time periods.

Dataset S5 (separate file). ROH estimates and inbreeding coefficients based on all ROHs, short ROHs and long ROHs for ancient and modern dogs and wolves.

Dataset S6 (separate file). (A) Gene ontology terms from the gene enrichment analysis of ROH deserts in dogs. (B) Gene ontology terms from the gene enrichment analysis of ROH deserts in dogs masking the DLA region.

Dataset S7 (separate file). (A) Gene ontology terms from the gene enrichment analysis of ROH deserts in wolves. (B) Gene ontology terms from the gene enrichment analysis of ROH deserts in wolves masking the DLA region.

Dataset S8 (separate file). Candidate genes from gene enrichment analysis.

Dataset S9 (separate file). Software and R packages used in the study.

SI References

1. P. Skoglund, E. Ersmark, E. Palkopoulou, L. Dalén, Ancient wolf genome reveals an early divergence of domestic dog ancestors and admixture into high-latitude breeds. *Curr. Biol.* **25**, 1515–1519 (2015).

2. L. A. F. Frantz, *et al.*, Genomic and archaeological evidence suggests a dual origin of domestic dogs. *Science* **352**, 1228–1231 (2016).

3. L. R. Botigué, *et al.*, Ancient European dog genomes reveal continuity since the Early Neolithic. *Nat. Commun.* **8** (2017).

4. M. Ní Leathlobhair, *et al.*, The evolutionary history of dogs in the Americas. *Science* **361**, 81–85 (2018).

5. A. Bergström, *et al.*, Origins and genetic legacy of prehistoric dogs. *Science* **370**, 557–564 (2020).

6. A. Bergström, *et al.*, Grey wolf genomic history reveals a dual ancestry of dogs. *Nat. 2022* **7** (2022).

7. M. S. Sinding, *et al.*, Arctic-adapted dogs emerged at the Pleistocene – Holocene transition. *Science* 1495–1499 (2020).

8. T. R. Feuerborn, *et al.*, Modern Siberian dog ancestry was shaped by several thousand years of Eurasian-wide trade and human dispersal. *Proc. Natl. Acad. Sci.* **118**, e2100338118 (2021).

9. J. Ramos-Madrigal, *et al.*, Genomes of Pleistocene Siberian Wolves Uncover Multiple Extinct Wolf Lineages. *Curr. Biol.* **31**, 198-206.e8 (2021).

10. P. Blaževičius, *et al.*, *Vilniaus pilių fauna nuo kepsnio iki draugo* (Vilniaus universiteto leidykla, 2018).

11. G. Piličiauskienė, P. Blaževičius, T. Zarankaitė-Margienė, *Šunys Lietuvoje XIII–XVIII amžiuje* (Vilniaus universiteto leidykla, 2023).

12. Lūsēns, "Ģertrūdes baznīcas kapsētā Rīgā. Arheologu pētījumi Latvijā 2006. un 2007" in (Latvijas Vēstures institūta, 2008), pp. 143–151.

13. J. Dabney, *et al.*, Complete mitochondrial genome sequence of a Middle Pleistocene cave bear reconstructed from ultrashort DNA fragments. *Proc. Natl. Acad. Sci.* **110**, 15758–15763 (2013).

14. C. Carøe, *et al.*, Single-tube library preparation for degraded DNA. *Methods Ecol. Evol.* **9**, 410–419 (2018).

15. M. Kircher, S. Sawyer, M. Meyer, Double indexing overcomes inaccuracies in multiplex sequencing on the Illumina platform. *Nucleic Acids Res.* **40**, e3 (2012).

16. M. Schubert, S. Lindgreen, L. Orlando, AdapterRemoval v2: Rapid adapter trimming, identification, and read merging. *BMC Res. Notes* **9**, 1–7 (2016).

17. H. Li, R. Durbin, Fast and accurate short read alignment with Burrows–Wheeler transform. *Bioinformatics* **25**, 1754–1760 (2009).

18. H. Li, Aligning sequence reads, clone sequences and assembly contigs with BWA-MEM. *ArXiv Prepr. ArXiv13033997* (2013).

19. K. Lindblad-Toh, *et al.*, Genome sequence, comparative analysis and haplotype structure of the domestic dog. *Nature* **438**, 803–819 (2005).

20. M. Kircher, "Analysis of High-Throughput Ancient DNA Sequencing Data" in *Ancient DNA:*

Methods and Protocols, Methods in Molecular Biology., B. Shapiro, M. Hofreiter, Eds. (Humana Press, 2012), pp. 197–228.

21. S. Rubinacci, D. M. Ribeiro, R. J. Hofmeister, O. Delaneau, Efficient phasing and imputation of low-coverage sequencing data using large reference panels. *Nat. Genet.* **53**, 120–126 (2021).

22. B. Sousa Da Mota, *et al.*, Imputation of ancient human genomes. *Nat. Commun.* **14**, 3660 (2023).

23. P. Danecek, *et al.*, Twelve years of SAMtools and BCFtools. *GigaScience* **10**, giab008 (2021).

24. G. A. van der Van der Auwera, Brian D. O'Connor, Genomics in the cloud : using docker, GATK, and WDL in terra. *No Title* (2020).

25. R. Poplin, *et al.*, Scaling accurate genetic variant discovery to tens of thousands of samples. [Preprint] (2018). Available at: https://www.biorxiv.org/content/10.1101/201178v3 [Accessed 20 November 2023].

26. J. Plassais, *et al.*, Whole genome sequencing of canids reveals genomic regions under selection and variants influencing morphology. *Nat. Commun.* **10**, 1489 (2019).

27. R. J. Hofmeister, D. M. Ribeiro, S. Rubinacci, O. Delaneau, Accurate rare variant phasing of whole-genome and whole-exome sequencing data in the UK Biobank. *Nat. Genet.* **55**, 1243–1249 (2023).

28. C. L. Campbell, C. Bhérer, B. E. Morrow, A. R. Boyko, A. Auton, A pedigree-based map of recombination in the domestic dog genome. *G3 Genes Genomes Genet.* **6**, 3517–3524 (2016).

29. T. S. Korneliussen, A. Albrechtsen, R. Nielsen, ANGSD: Analysis of Next Generation Sequencing Data. *BMC Bioinformatics* **15**, 1–13 (2014).

30. N. Patterson, A. L. Price, D. Reich, Population Structure and Eigenanalysis. *PLoS Genet.* **2**, e190 (2006).

31. C. C. Chang, *et al.*, Second-generation PLINK: rising to the challenge of larger and richer datasets. *GigaScience* **4**, 7 (2015).

32. F. C. Ceballos, *et al.*, Human inbreeding has decreased in time through the Holocene. *Curr. Biol.* **31**, 3925-3934.e8 (2021).

33. H. Schroeder, *et al.*, Origins and genetic legacies of the Caribbean Taino. *Proc. Natl. Acad. Sci.* **115**, 2341–2346 (2018).

34. D. W. Clark, *et al.*, Associations of autozygosity with a broad range of human phenotypes. *Nat. Commun.* **10**, 4957 (2019).

35. O. Aramburu, *et al.*, Genomic Signatures After Five Generations of Intensive Selective Breeding: Runs of Homozygosity and Genetic Diversity in Representative Domestic and Wild Populations of Turbot (Scophthalmus maximus). *Front. Genet.* **11** (2020).

36. E. Lavanchy, J. Goudet, Effect of reduced genomic representation on using runs of homozygosity for inbreeding characterization. *Mol. Ecol. Resour.* **23**, 787–802 (2023).

37. G. Renaud, K. Hanghøj, T. S. Korneliussen, E. Willerslev, L. Orlando, Joint Estimates of Heterozygosity and Runs of Homozygosity for Modern and Ancient Samples. *Genetics* **212**, 587–614 (2019).

38. M. Lawrence, *et al.*, Software for Computing and Annotating Genomic Ranges. *PLOS Comput. Biol.* **9**, e1003118 (2013).

39. D. Chicco, G. Jurman, The advantages of the Matthews correlation coefficient (MCC) over F1

score and accuracy in binary classification evaluation. *BMC Genomics* **21**, 6 (2020).

40. Ben Gorman, mltools: Machine Learning Tools. (2018). Deposited 2018.

41. F. C. Ceballos, P. K. Joshi, D. W. Clark, M. Ramsay, J. F. Wilson, Runs of homozygosity: Windows into population history and trait architecture. *Nat. Rev. Genet.* **19**, 220–234 (2018).

42. M. A. Stoffel, S. E. Johnston, J. G. Pilkington, J. M. Pemberton, Genetic architecture and lifetime dynamics of inbreeding depression in a wild mammal. *Nat. Commun.* **12**, 2972 (2021).

43. J. R. S. Meadows, *et al.*, Genome sequencing of 2000 canids by the Dog10K consortium advances the understanding of demography, genome function and architecture. *Genome Biol.* **24**, 187 (2023).

44. S. Grote, GOfuncR: Gene ontology enrichment using FUNC. (2023). Deposited 2023.

45. C. Wang, *et al.*, A novel canine reference genome resolves genomic architecture and uncovers transcript complexity. *Commun. Biol.* **4**, 1–11 (2021).
